# Stabilization toward air and structure determination of pyrophoric ZnR_2_ compounds via supramolecular encapsulation

**DOI:** 10.1126/sciadv.adt7372

**Published:** 2025-04-25

**Authors:** Kamil Sokołowski, Iwona Justyniak, Michał Terlecki, David Fairen-Jimenez, Wojciech Bury, Krzysztof Budny-Godlewski, Jan Nawrocki, Marek Kościelski, Janusz Lewiński

**Affiliations:** ^1^ Institute of Physical Chemistry, Polish Academy of Sciences, Kasprzaka 44/52, 01-224 Warsaw, Poland.; ^2^ Faculty of Chemistry, Warsaw University of Technology, Noakowskiego 3, 00-664 Warsaw, Poland.; ^3^ Department of Chemical Engineering and Biotechnology, University of Cambridge, Pembroke Street, Cambridge CB2 3RA, UK.

## Abstract

Dialkylzincs (ZnR_2_, R = Me or Et) are widely used reagents in organic synthesis and materials chemistry. However, at standard conditions, they exist as pyrophoric liquids reacting violently with water and dioxygen, thus being dangerous and difficult to use in daily laboratory work. Here, we show that these zinc dialkyls can be efficiently stabilized toward air by supramolecular encapsulation within a host system based on heteroleptic alkylzinc complexes. The noncovalent immobilization of ZnR_2_ molecules within the resultant crystalline networks allows their structural characterization in a new confined environment. The great potential of the reported assemblies is demonstrated by efficient separation of ZnMe_2_ from a mixture of ZnMe_2_/ZnEt_2_. The reported approach paves the way for original supramolecular systems for capture, stabilization, and storage of dangerous reagents.

## Teaser

Efficient encapsulation of pyrophoric ZnR_2_ compounds paves the way for their selective capture, air-handling, and storage.

## INTRODUCTION

The microenvironment of subnanometric confined spaces determines the properties of incorporated chemical species. The design of encapsulating environments is an extremely fast developing area of research (*1*–*4*), which covers a range of host molecular structures including organic cavitands (*5*–*7*), supramolecular organic capsules assembled via covalent or hydrogen bonds (*8*–*10*), discrete coordination cages (*11*–*14*), and, more recently, hydrogen-bonded organic frameworks (*15*–*17*), porous metal-organic frameworks (*18*, *19*), as well as matrices of molecular crystals (*20*–*24*). In analogy to natural enzymes, supramolecular encapsulation leads to unusual behavior of guest molecules within well-defined inner cavities of a host material (*4*) facilitating stoichiometric and catalytic transformations (*25*, *26*), enabling (enantio)separation of small organic molecules (*20*, *27*–*30*), and stabilizing unusual reaction intermediates (*22*, *31*, *32*). Furthermore, the entrapment of molecules within porous frameworks has also been found as a promising tool for structure determination of noncrystalline, volatile substances or labile, elusive species (*22*, *31*, *33*). Currently, many efforts are mounting to the development of frameworks enabling the encapsulation of reactive agents (*34*–*37*) and the pacification of dangerous chemicals, such as chemical warfare agents (*38*, *39*). Similar interest applies to sensitive organometallic reagents, and this demand has recently driven the development of efficient stabilization environments, which for organolithium and other air-sensitive reagents were realized through incorporation within bulky organogels or paraffin shells (*40*–*42*). The enormity of this issue is obvious, yet an encouraging start has been made.

Homoleptic diorganozinc compounds (ZnR_2_) are a staple of organometallic chemistry since Frankland’s pioneering discoveries (*43*), with constantly increasing importance from modern catalysis and synthetic organic chemistry (*44*–*48*) to nanoscience (*49*–*52*). The lowest ZnR_2_ homologs, dimethyl and diethylzinc (ZnMe_2_ and ZnEt_2_, respectively) at standard conditions exist as volatile pyrophoric liquids [ZnMe_2_: melting point (mp) −42°C, boiling point (bp) 46°C; ZnEt_2_: mp −28°C, bp 118°C], which react violently with water and dioxygen, ignite readily upon contact with air, and therefore need to be handled in an inert atmosphere. These features represent a serious drawback for their practical use in daily laboratory work as well as on an industrial scale. For organozincs, to date, reasonable stability of substantially less reactive heteroleptic RZn(L)–type complexes has been only achieved via their incorporation within heterometallic solid salt–stabilized system allowing for air-handling from minutes to several hours (*53*, *54*). However, the systems enabling efficient stabilization in the air of highly pyrophoric homoleptic diorganozinc ZnR_2_ compounds are still lacking. Herein, we demonstrate a strategy for efficient encapsulation of ZnMe_2_ and ZnEt_2_ within a dyad of self-assembling simple heteroleptic alkylzinc molecules. The high-quality crystals of the resultant supramolecular capsules grow rapidly (minutes) with a high yield from neat dialkylzincs. This supramolecular confinement of homoleptic zinc alkyls within the crystalline matrices enables (i) air stabilization of pyrophoric ZnR_2_ compounds, (ii) efficient separation of a ZnMe_2_/ZnEt_2_ mixture, and (iii) full-structural characterization of volatile ZnR_2_ compounds confined in subnanometric spaces. Furthermore, we show that the immobilized ZnR_2_ molecules can be readily released from the host framework by both mild heating or dissolution in an organic solvent.

## RESULTS AND DISCUSSION

### Synthesis and characterization

**Fig. 1.**
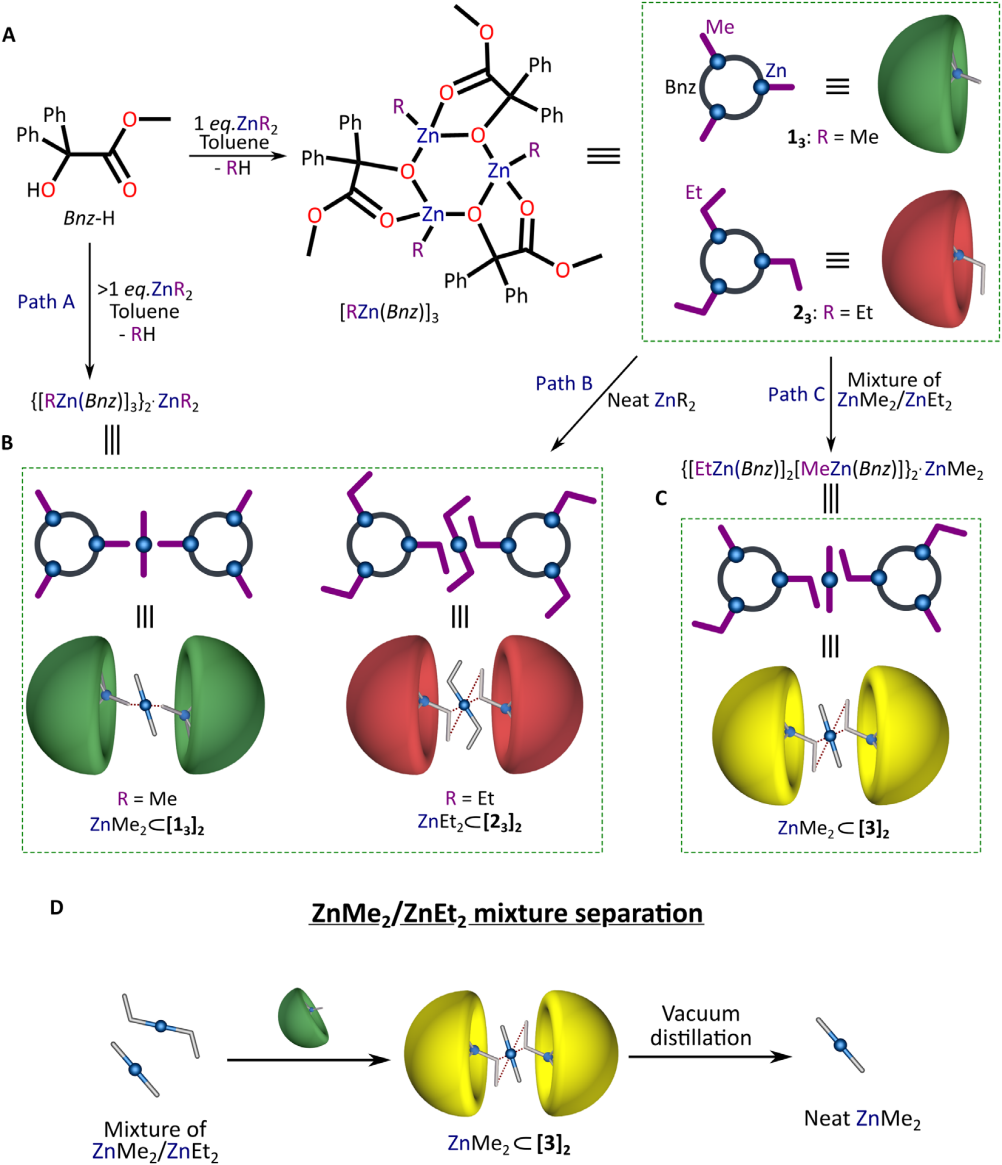
Synthetic pathways leading to supramolecular encapsulation of ZnR_2_ molecules. (**A**) Synthesis and isolation of **1**_**3**_ and **2**_**3**_ from toluene solutions. (**B**) Two pathways (A and B) for the isolation of supramolecular host-guest systems ZnMe_2_ ⊂ [**1**_**3**_]_2_ and ZnEt_2_ ⊂ [**2**_**3**_]_2_. (**C**) Formation of supramolecular host-guest system ZnMe_2_ ⊂ [**3**]_2_ by the selective encapsulation of ZnMe_2_ from a mixture of ZnMe_2_/ZnEt_2_ (path C). (**D**) Schematic representation of the concept of ZnMe_2_/ZnEt_2_ mixture separation using ZnMe_2_ ⊂ [**3**]_2_ host-guest system.

Our long-standing interest both in the design of molecular entities based on achiral (*55*–*57*) and chiral (*20*, *58*, *59*) organometallic complexes prone to self-assemble in desired supramolecular systems, and in search for model zinc alkoxide systems fulfilling the Kagan’s theoretical criteria for the nonlinear effects in asymmetric synthesis (*60*) involving the donor-functionalized-alcohol promoted addition of dialkylzinc reagents to carbonyl compounds (*45*, *61*), we turned our attention to alkylzinc systems supported by benzilate ligands incorporating a Ph_2_C unit, dubbed “magic” on the account of its peculiar auxiliary character in catalysis (*62*, *63*). Initially, in an equimolar reaction between ZnR_2_ and methyl benzilate (*Bnz*-H) in toluene, we prepared methyl- and ethylzinc [RZn(*Bnz*)]_n_–type complexes, which in solution exist in dynamic equilibrium between dimeric [RZn(*Bnz*)]_2_ and trimeric [RZn(*Bnz*)]_3_ aggregates as indicated by diffusion-ordered nuclear magnetic resonance spectroscopy (see section S1.2). Trimeric complexes [RZn(*Bnz*)]_3_ [where R = Me (**1**_**3**_) and Et (**2**_**3**_); Fig. 1A] were isolated quantitatively upon crystallization from the post-reaction mixture at −20°C as solvates incorporating toluene molecules, **1**_**3**_·3*Me*Ph and **2**_**3**_·0.5*Me*Ph (figs. S14 to S17).

### Molecular recognition

We found that the addition of ZnMe_2_ or ZnEt_2_ (i.e., > 0.25 equiv.) to a toluene solution of **1**_**3**_ or **2**_**3**_, respectively, led to self-assembly–driven encapsulation of the respective ZnR_2_ molecules by supramolecular dyads of the corresponding trimeric alkylzinc benzilate aggregates (Fig. 1B, path A). Upon crystallization, {[MeZn(*Bnz*)]_3_}_2_·ZnMe_2_ (ZnMe_2_ ⊂ [**1**_**3**_]_2_) and {[EtZn(*Bnz*)]_3_}_2_·ZnEt_2_ (ZnEt_2_ ⊂ [**2**_**3**_]_2_) supramolecular host-guest systems were isolated and structurally characterized (figs. S18 to S25). Another unique feature of **1**_**3**_ and **2**_**3**_ host systems is their ability to entrap ZnR_2_ molecules directly from their respective neat forms. Complexes **1**_**3**_ and **2**_**3**_ can be readily dissolved in neat ZnR_2_ followed by an instantaneous crystallization of ZnMe_2_ ⊂ [**1**_**3**_]_2_ and ZnEt_2_ ⊂ [**2**_**3**_]_2_ (Fig. 1B, path B). The visible crystals appear within tens of seconds leading to high-quality single-crystalline material (fig. S22). In turn, the dissolution of **1**_**3**_ or **2**_**3**_ in an equimolar mixture of neat ZnMe_2_ and ZnEt_2_ is followed by rapid formation of a peculiar host-guest supramolecular system {[MeZn(*Bnz*)][EtZn(*Bnz*)]_2_}·ZnMe_2_ (ZnMe_2_ ⊂ [**3**]_2_) (Fig. 1C, path C) incorporating selectively the smaller ZnMe_2_ analog as the guest entity (figs. S24 and S25). This ability of complex **3** to selectively intercept a pretty volatile ZnMe_2_ from a mixture of both homologs could be a competitive separation alternative to their fractional distillation (Fig. 1D). The trimeric host **3** is composed of one methyl- and two ethyl-zinc moieties, which indicates that its formation is preceded by the alkyl group exchange between the homoleptic and heteroleptic species [such observation is uncommon and previously we were able to describe the transalkylation among Al and Zn metal centers (*59*)]. The composition and structure of the complexes **1**_**3**_ and **2**_**3**_ as well as the ZnMe_2_ ⊂ [**1**_**3**_]_**2**_, ZnEt_2_ ⊂ [**2**_**3**_]_2_, and ZnMe_2_ ⊂ [**3**]_2_ supramolecular systems were confirmed by nuclear magnetic resonance (NMR) and Fourier transform infrared (FTIR) spectroscopy, elemental analysis, and single-crystal x-ray diffraction. The ^1^H NMR ([D_8_]-toluene) spectra of **1**_**3**_, **2**_**3**_, ZnMe_2_ ⊂ [**1**_**3**_]_2_ and ZnEt_2_ ⊂ [**2**_**3**_]_2_, and ZnMe_2_ ⊂ [**3**]_2_ were fully consistent with the anticipated formulas (see section S1).

### Factors controlling molecular recognition and self-assembly processes

**Fig. 2.**
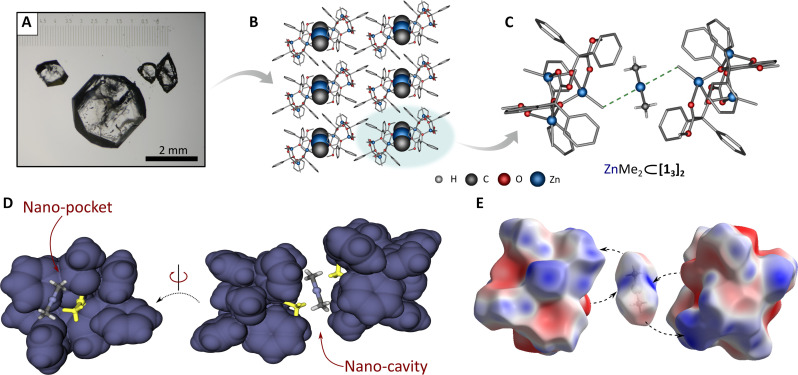
The formation and composition of supramolecular capsules ZnMe_2_ ⊂ [1_3_]_2_. (**A**) Optical microscope photo of the crystals. (**B**) Arrangement of the capsules in the crystal lattice (view along *a* crystal axis, guest molecules are in a space-filling model). (**C**) Structure of the single supramolecular capsule; (**D**) composition of nano-cavity between molecules of **1**_**3**_. (**E**) Hirshfeld surfaces of the host-guest system components mapped with electrostatic potential [from −0.05 au (red) to +0.05 au (blue); the individual components have been moved apart to show contact between the surfaces].

Molecular and crystal structure analysis reveals unique structural features of the **1**_**3**_, **2**_**3**_, or **3** host molecular system supporting the molecular recognition of ZnR_2_ species and self-assembly of the final supramolecular matrices. The basic molecular entities of these host systems comprise three [MeZn(*Bnz*)] and/or [EtZn(*Bnz*)] units bridged up by the alkoxide oxygen atoms of *Bnz* ligands forming central six-membered [Zn(μ_2_-O)]_3_ rings (see section S2). The resulting [Zn(μ_2_-O)]_3_ ring constitutes the base of a nest surrounded by the organic ligands’ backbones. The overall shape of the nest is quasi-spherical with three rounded hollow sides of the baseball glove–like fashion (dubbed further as nano-pockets), decorated with phenyl and ester groups from the *Bnz* ligands, and centrally directed Zn-bonded alkyl groups (Fig. 2D). Complexes **1**_**3**_ and **2**_**3**_ crystallize from toluene as **1**_**3**_ 3*Me*Ph and **2**_**3**_ 0.5*Me*Ph solvates, respectively, that differ in the arrangement of solvating molecules in the respective crystal lattices (figs. S15 and S17); we note that toluene molecules do not exhibit directional interactions with **1**_**3**_ and **2**_**3**_ clusters. When an excess of ZnR_2_ in the parent solution is present, the crystallization renders the respective pair of nests as the highly selective host system toward efficient encapsulation of ZnR_2_ molecules (Fig. 1B, path A). Thus, every molecule of **1**_**3**_, **2**_**3**_, or **3** uses one of its nano-pockets for molecular recognition–driven interactions with a ZnR_2_ molecule and the resulting nano-pocket dyads act as supramolecular containers with subnanometer cavities (Fig. 2). The nano-cavities comprise two Zn-R functionalities, each from the neighbor nano-pocket, directed toward the central part of the construct, which function as a type of anchored antennas for the molecular recognition of ZnR_2_ guests; note that two other nano-pockets of the host molecules located on the outward of the capsule do not participate in encapsulation events. Strikingly, despite the presence of accessible Lewis base centers in the [RZn(*Bnz*)] units (ester and alkoxy groups), the excess ZnR_2_ molecules do not form any dative bonds with the host molecules (*61*). Instead, the encapsulated dialkylzinc molecule is involved in intermolecular noncovalent CH_alkyl_···Zn interactions between its Zn center and the Zn-R antenna moieties in the inner sphere of the nano-cavity (Fig. 2C and figs. S19, S21, and S25). Analysis of the Hirshfeld surfaces displays good shape and electrostatic complementarity between the inner cavity of the capsules and guest ZnR_2_ molecules, which likely drive the formation of the studied host-guest systems (Fig. 2E; for details, see section S3). The resulting ZnMe_2_ ⊂ [**1**_**3**_]_2_, ZnEt_2_ ⊂ [**2**_**3**_]_2_, and ZnMe_2_ ⊂ [**3**]_2_ supramolecular capsules self-organize further by cooperative noncovalent forces to form isostructural closed-packed three-dimensional lattices in which ZnMe_2_ or ZnEt_2_ molecules are located in the *ab* planes separated from each other by ~17.4 to 17.8 Å (Fig. 2B and figs. S19, S21, and S25).

The revealed encapsulation system is a robust crystalline sponge driven by noncovalent interaction, which provides the opportunity to analyze the effect of a solid environment on the molecular structures of the entrapped ZnR_2_ compounds. In the case of the entrapped ZnMe_2_ molecules, the character of the solid environment affects Zn-C bond lengths, which are slightly longer in ZnMe_2_ ⊂ [**3**]_2_ (1.979 Å) than in ZnMe_2_ ⊂ [**1**_**3**_]_2_ (1.952 Å) (fig. S31). Moreover, both these values are slightly longer than that observed in the previously reported crystal structure of neat ZnMe_2_ (1.927 Å) (*64*) and the corresponding values obtained from our ab initio calculations (1.930 Å) (see section S11). In turn, within ZnEt_2_ ⊂ [**2**_**3**_]_2_, the entrapped ZnEt_2_ molecules exhibit similar Zn-C bond lengths (1.944 Å) to that observed in the crystal structure of neat ZnEt_2_ (1.948 Å) (*64*) and obtained from our ab initio calculations (1.950 Å). Notably, CH_alkyl_···Zn intermolecular interactions between the antenna Zn-R moieties in the interior of supramolecular containers and the Zn atoms of the encapsulated ZnR_2_ molecule resemble the crystal environment of ZnR_2_ molecules in their condensed forms (fig. S31).

On the basis of the crystallographic data, we performed classical molecular simulation for binding energy to gain more in-depth insight into the interaction between the dialkylzinc guest molecules and the hollow hosts (for details, see section S11). The molecular simulations suggest the existence of important steric effects that influence the overall stability of the supramolecular capsules and possibly trigger the specific uptake of ZnMe_2_ by **3** from the ZnMe_2_/ZnEt_2_ mixture. The binding energy of ZnMe_2_ ⊂ [**3**]_2_ (−99.1 kJ/mol) falls between that found for ZnEt_2_ ⊂ [**2**_**3**_]_2_ (strongest, −114.7 kJ/mol) and ZnMe_2_ ⊂ [**1**_**3**_]_2_ (weakest, −97.6 kJ/mol). However, a detailed examination of the energy contributions (i.e., van der Waals and electrostatic, see table S13) shows the existence of essential electrostatic repulsions in the quest-captured ZnEt_2_ ⊂ [**2**_**3**_]_2_ system, a situation that does not arise for ZnMe_2_ ⊂ [**1**_**3**_]_2_ and ZnMe_2_ ⊂ [**3**]_2_. Therefore, the selective separation of ZnMe_2_ from the mixture of ZnMe_2_/ZnEt_2_ and the formation of ZnMe_2_ ⊂ [**3**]_2_ can be regarded as a result of specific host-guest interactions between the ZnMe_2_ guest and the **3** hosts (Fig. 1D).

### Solid supramolecular stabilization of ZnR_2_ compounds toward air

**Fig. 3.**
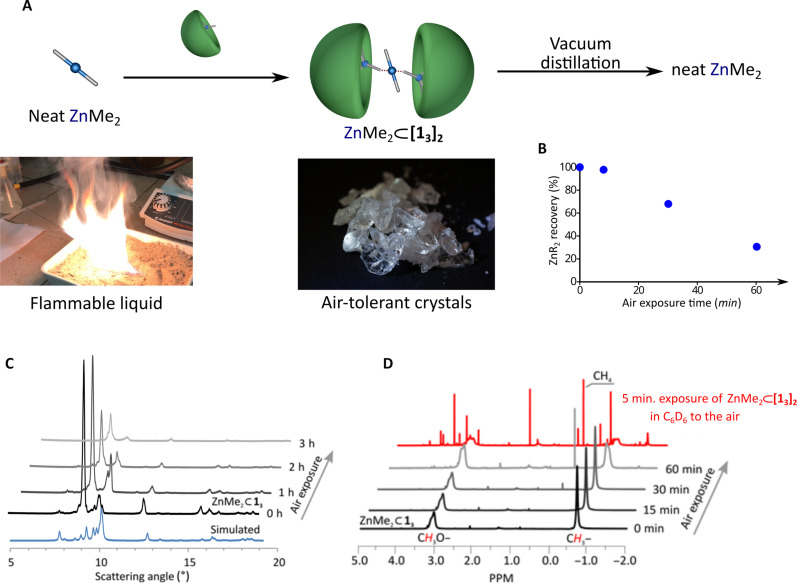
Stability of supramolecular capsules toward air. (**A**) Schematic representation of the supramolecular stabilization of ZnMe_2_ toward air within [**1**_**3**_]_2_ capsules. (**B**) Dependence of the extracted amount of ZnR_2_ on the air exposure time of the capsules. (**C**) Time-dependent in situ PXRD patterns of ZnMe_2_ ⊂ [**1**_**3**_]_**2**_ upon exposition to air. h, hours. (**D**) ^1^H NMR spectra in C_6_D_6_ collected after dissolution of the crystalline samples of ZnMe_2_ ⊂ [**1**_**3**_]_2_ stored in air as well as after the exposition of C_6_D_6_ solution of ZnMe_2_ ⊂ [**1**_**3**_]_2_ in air for 5 min.

The described supramolecular containers [**1**_**3**_]_2_, [**2**_**3**_]_2_, and [**3**]_2_ also provide efficient stabilization toward air of highly pyrophoric ZnMe_2_ and ZnEt_2_ compounds, as evidenced by in situ powder x-ray diffraction (PXRD) and spectroscopic studies (see section S5). The PXRD patterns showed no change in the crystal structure of ZnMe_2_ ⊂ [**1**_**3**_]_2_ and ZnEt_2_ ⊂ [**2**_**3**_]_2_ during the exposition of their crystals to air at room temperature up to 1 hour (Fig. 3C and fig. S32). Similarly, the ^1^H NMR spectra in C_6_D_6_ collected after dissolution of the guest-captured ZnMe_2_ ⊂ [**1**_**3**_]_2_ and ZnEt_2_ ⊂ [**2**_**3**_]_2_ samples stored in air by up to 1 hour show signals from alkyl groups of ZnR_2_ compounds (Fig. 3D and fig. S33); note that the exposition of ZnMe_2_ ⊂ [**1**_**3**_]_2_ and ZnEt_2_ ⊂ [**2**_**3**_]_2_ in C_6_D_6_ solution causes the vanishing of the respective signals after only 5 min. This observation demonstrates that the protected encapsulated ZnR_2_ molecules can be readily released by simple dissolution of ZnR_2_-filled capsules. Moreover, thermogravimetry analysis (TGA) of ZnMe_2_ ⊂ [**1**_**3**_]_2_ and ZnEt_2_ ⊂ [**2**_**3**_]_2_ samples indicates that pure ZnMe_2_ and ZnEt_2_ compounds can be extracted from the respective supramolecular containers by heating at ~120°C (figs. S37 and S38). This was further confirmed experimentally by the thermal vacuum–assisted evacuation of ZnEt_2_ form ZnEt_2_ ⊂ [**2**_**3**_]_2_ (120°C, 20 min), yielding an almost quantitative recovery of the encapsulated organometallic guest with high purity, as evidenced by ^1^H NMR (see section S7). Notably, the separated **2**_**3**_ host retains its molecular composition despite forming a new unsolvated crystal phase and can be reused to encapsulate another batch of the respective dialkyzinc compounds. Exposure of ZnEt_2_⊂[**2**_**3**_]_2_ crystals to air for up to 10 min did not substantially affect the yield of extracted ZnEt_2_ (98% recovery; see section S7). However, prolonged exposure gradually reduced the recovery yield, allowing the extraction of 67 and 32% of the initial encapsulated organometallic compound after 30 and 60 min of exposure, respectively (Fig. 3C). Notably, ^1^H NMR spectra analysis shows no indication of the formation of alkoxide OR groups upon the interaction of ZnR_2_-loaded containers with air but only the evolution of respective alkane (fig. S34). This suggests that the guest molecules are relatively well protected from O_2_ and that the slow degradation of the capsules is primarily due to their reaction with moisture from the air. The gradual penetration of water into the material allows the crystal structure of the initial capsules to be roughly preserved, as evidenced by PXRD analysis.

The efficient shielding against the air of ZnR_2_ molecules in the studied containers was also examined by computational analysis of the pore network accessibility and pore size distribution (PSD) (see section S10). The use of probe molecules with different sizes revealed a very narrow PSD in the empty host material centered at 3.5 Å, which is completely saturated when encapsulating the ZnR_2_ guest molecules (fig. S51). The close encapsulation of organozinc molecules within [**1**_**3**_]_2_, [**2**_**3**_]_2_, and [**3**]_2_ capsules are also manifested by the tight shape of Hirshfeld surfaces, calculated for guest molecules, with no long-range contact regions (figs. S18, S19, and S20). The perfect fit of the guest molecules in the host material impeding the access of dioxygen or water molecules to the porosity appears as the main factor for the neutralization of ZnR_2_ molecules.

### Toward supramolecular containers as functional materials

**Fig. 4.**
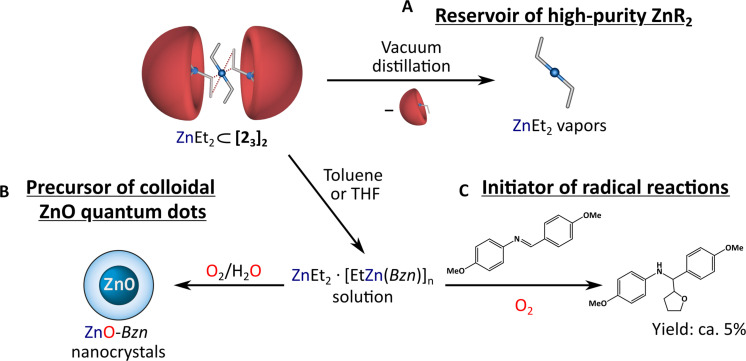
Emerging potential applications of ZnEt_2_ ⊂ [2_3_]_2_. (**A**) Schematic representation of the vacuum-assisted releasing of ZnEt_2_. (**B**) Utilization of ZnEt₂ ⊂ **[2**_**3**_**]₂** as the efficient air-resistant precursor of the high-quality *Bzn*-capped ZnO NCs. (**C**) Limited activity of ZnR_2_ compounds released from ZnEt₂ ⊂ **[2**_**3**_**]₂** as an initiator of the model radical reaction.

The great potential of the reported supramolecular encapsulation system is evident in its demonstrated above ability to selectively capture, stabilize, and store homoleptic zinc alkyls. The documented vacuum-assisted evacuations of ZnEt_2_ from the ZnEt_2_ ⊂ [**2**_**3**_]_2_ material appears to be another promising application of the title supramolecular containers as easy-to-handle prospective reservoirs of high-quality ZnR_2_ reagents for atomic layer deposition and metal-organic chemical vapor deposition techniques (Fig. 4A), where vapors of ZnR_2_ compounds are commonly used as Zn sources (*50*, *65*). Fascinating characteristics of these supramolecular containers further expand their applications into other areas of materials science. For example, in recent years, low-temperature wet-organometallic approaches based on homoleptic (*49*, *51*, *52*) or heteroleptic (*66*–*68*) organozinc precursors have been developed for the preparation of colloidal ZnO nanocrystals (NCs). In our case, we hypothesized that the supramolecular containers containing both homoleptic and heteroleptic organozincs could serve as an original type of precursor for ZnO NCs. This prediction was subsequently verified through an experiment using the general one-pot self-supporting organometallic method (OSSOM) developed by our group (*66*, *67*). Accordingly, a sample of ZnEt_2_ ⊂ [**2**_**3**_]_2_ was dissolved in tetrahydrofuran (THF) and stirred under air at room temperature for 4 days, ultimately leading to the formation of quantum-sized ZnO NCs capped by *Bnz* ligands (Fig. 4B; see section S9). The resulting NCs, with an average diameter of 6.6 ± 1.8 nm, form stable colloidal solutions in dimethyl sulfoxide (DMSO) and MeOH and exhibit yellow luminescence under ultraviolet (UV) light [λ_em_= 575 nm, photoluminescence quantum yields (PLQY) = 9.2%]. Furthermore, they exhibit relatively long PL charge recombination with average decay time of 2.0 μs, which supports high-quality organic-inorganic interface characteristic for OSSOM-derived ZnO NCs (*52*, *69*).

Recently, we have provided a fresh look at organic radical chemistry mediated by homoleptic and heteroleptic zinc alkyls in the presence of dry air (*47*). Hence, in another control experiment, we also tested ZnEt₂ ⊂ [**2**_**3**_]_2_ capsules as a radical initiator in a model reaction of radical addition of THF to imines. However, the benchmark experiment demonstrated its limited activity, yielding only 5% of the THF-imine adduct (Fig. 4C; for details, see section S8). This can be explained by the fact that upon dissolution of ZnEt₂ ⊂ [**2**_**3**_]_2_ capsules, the liberated ZnEt_2_ molecules may form Lewis acid–base adducts with the simultaneously introduced heteroleptic alkylzinc benzylate [EtZn(*Bnz*)] moieties, modifying the reactivity of the dialkylzinc compounds (*61*, *70*). These processes likely bring the phenyl rings of the benzylate ligands into close proximity with Zn-R species, which may substantially influence the oxygenation reaction (*71*), promoting the evolution of R* radicals over OR*, thereby reducing the effectiveness of ZnEt₂ ⊂ [**2**_**3**_]_2_ as radical initiators (*47*). The described examples clearly demonstrate that the reported supramolecular containers could potentially find applications in various fields of materials and synthetic chemistry.

In conclusion, we have demonstrated an effective strategy for encapsulating pyrophoric homoleptic dialkylzinc compounds. The presented supramolecular system consists of dyads based on simple heteroleptic alkylzinc complexes, which efficiently encapsulate ZnR₂ reagents, providing effective shielding against air and allowing their structure determination in a peculiar solid environment. The great potential of the reported assemblies is further demonstrated by efficient separation of ZnMe_2_ from a mixture of ZnMe_2_/ZnEt_2_. The encapsulated ZnR_2_ molecules can be readily released from the assemblies through simple temperature treatment or dissolution in an organic solvent. This approach paves the way for the development of supramolecular systems for the capture, stabilization of hazardous reagents within molecular matrices. Their potential was explored through initial experiments on the vacuum-assisted recovery of the ZnR_2_ guests from ZnEt_2_-loaded capsules as well as utilization of the capsules as original type of precursor for the preparation of quantum-sized ZnO NCs and as initiator in radical-mediated organic reactions.

## MATERIALS AND METHODS

### General remarks

Unless stated otherwise, all manipulations were conducted under a nitrogen atmosphere by using standard Schlenk techniques. All reagents were purchased from commercial vendors: ZnMe_2_ (ABCR) and ZnEt_2_ (ABCR), methyl benzilate (ABCR) and used as received. Solvents were purified and dried using MBraun Solvent Purification System or distillation before use.

### Synthesis of 1_3_·3PhMe

ZnMe_2_ (0.096 g, 1.00 mmol) was added to a solution of *Bnz*-H (0.242 g, 1.00 mmol) in toluene (4.0 mL) at −78°C. The reaction mixture was allowed to warm to room temperature, and the resulting solution was stirred vigorously for a further 6 hours. Colorless crystals were obtained from concentrated toluene solution at −20°C (0.460 g, isolated yield 77%). Elemental analysis (%) calcd for C_69_H_72_O_9_Zn_3_ (1241.37 g mol^−1^): C 66.74, H 5.80, O 11.64, Zn 15.82 (%). Found: C 66.81, H 5.82, O 11.66 (%); ^1^H NMR ([D8]-toluene): δ = 7.65 [br s, 4H, C*H*_(Ar)_], 7.22 [br s, 4H, C*H*_(Ar)_], 7.12 [br s, 2H, C*H*_(Ar)_], 7.10 [s, 0.33H, C*H*_(Ar-Tol)_], 7.10 [s, 0.33H, C*H*_(Ar-Tol)_], 7.02 [s, 0.16H, C*H*_(Ar-Tol)_], 6.98 [s, 0.33H, C*H*_(Ar-Tol)_], 3.16 [br s, 3H, OC*H*_3_], 2.10 [m, 0.5H, C*H*_3(Tol)_], −0.79 [s, 3H, C*H*_3_]; IR: ν = 1682 s, 1600 w, 1493 m, 1446 s, 1436 s, 1377 w, 1371 w, 1272 s, 1210 m, 1165 s, 1092 s, 1061 s, 1032 m, 994 m, 954 w, 913 w, 812 m, 759 s, 729 s, 697 s, 680 s, 643 s, 621 m, 604 s cm^−1^.

### Synthesis of 2_3_·0.5PhMe

ZnEt_2_ (0.124 g, 1.00 mmol) was added to a solution of *Bnz*-H (0.242 g, 1.00 mmol) in toluene (4.0 ml) at −78°C. The reaction mixture was allowed to warm to room temperature and the resulting solution was stirred vigorously for a further 6 hours. Colorless crystals were obtained from concentrated toluene solution at −20°C (0.304 g, isolated yield 71%). Elemental analysis (%) calcd for C_109_H_116_O_18_Zn_6_ (2106.23 g mol^−1^): C 52.10, H 5.50, O 13.67, Zn 18.63 (%). Found: C 52.23, H 5.55, O 13.70 (%); ^1^H NMR ([D8]-toluene): δ = 7.61 [br s, 4H, C*H*_(Ar)_], 7.22 [br s, 4H, C*H*_(Ar)_], 7.12 [br s, 2H, C*H*_(Ar)_], 7.10 [s, 0.33H, C*H*_(Ar-Tol)_], 7.10 [s, 0.33H, C*H*_(Ar-Tol)_], 7.02 [s, 0.16H, C*H*_(Ar-Tol)_], 6.98 [s, 0.33H, C*H*_(Ar-Tol)_], 3.24 [br s, 3H, OC*H*_3_], 2.10 [m, 0.5H, C*H*_3(Tol)_], 0.99 [t, 3H, CH_2_C*H*_3_], 0.01 [q, 2H, C*H*_2_CH_3_] parts per million (ppm); IR: ν = 1686 s, 1599 w, 1492 m, 1446 s, 1436 s, 1377 w, 1371 w, 1270 s, 1210 m, 1165 s, 1092 s, 1060 s, 1032 m, 994 m, 953 w, 911 w, 810 m, 759 s, 728 s, 697 s, 681 s, 634 s, 620 m, 606 s cm^−1^.

### Synthesis of ZnMe_2_ ⊂ [1_3_]_2_

#### Method A

From toluene solution: ZnMe_2_ (0.120 g, 1.25 mmol) was added to a solution of *Bnz*-H (0.242 g, 1.00 mmol) in toluene (4.0 ml) at −78°C. The reaction mixture was allowed to warm to room temperature, and the resulting solution was stirred vigorously for a further 6 hours. Colorless crystals were obtained from slightly concentrated toluene solution at 25°C (0.134 g, isolated yield 29% with reference to entrapped excess of ZnMe_2_).

#### Method B

Recrystallization of **1**_**3**_·3PhMe in neat ZnMe_2_: Crystals of **1**_**3**_·3PhMe (0.300 g, 0.24 mmol) were dissolved in ZnMe_2_ (0.7 ml). After 10 min, colorless crystals of ZnMe_2_ ⊂ [1_3_]_2_ were obtained from the prepared solution at 25°C (0.225 g, isolated yield 92%). The resulting material can be readily dissolved in toluene, benzene, chloroform, dichloroethane, and THF. Elemental analysis (%) calcd for C_98_H_102_O_18_Zn_7_ (2025.38 g mol^−1^): C 58.08, H 5.04, O 14.22, Zn 22.66 (%). Found: C 58.19, H 5.06, O 14.25 (%); ^1^H NMR ([D8]-toluene): δ = 7.64 [br s, 4H, C*H*_(Ar)_], 7.22 [br s, 4H, C*H*_(Ar)_], 7.12 [br s, 2H, C*H*_(Ar)_], 7.10 [s, 0.33H, C*H*_(Ar-Tol)_], 7.10 [s, 0.33H, C*H*_(Ar-Tol)_], 7.02 [s, 0.16H, C*H*_(Ar-Tol)_], 6.98 [s, 0.33H, C*H*_(Ar-Tol)_], 3.15 [br s, 3H, OC*H*_3_], 2.10 [m, 0.5H, C*H*_3(Tol)_], −0.78 [s, 4H, C*H*_3_]; CP-MAS ^13^C NMR: δ = 182.70 [*C*OOMe], 146.15 [*C*H_(Ar)_], 144.94 [*C*H_(Ar)_]; 143.86 [*C*H_(Ar)_]; 142.00 [*C*H_(Ar)_]; 141.12 [*C*H_(Ar)_]; 138.32 [*C*H_(Ar)_]; 127.34 [*C*H_(Ar)_]; 84.77 [O*C*H_3_]; 84.10 [O*C*H_3_]; 83.56 [O*C*H_3_]; 54.64 [*C*(Ph)_2_(OH)(C)]; 52.94 [*C*(Ph)_2_(OH)(C)]; 51.94 [*C*(Ph)_2_(OH)(C)]; −5.93 [H_3_*C*Zn]; −10.65 [H_3_*C*Zn]; −12.62 [H_3_*C*Zn]; −15.00 [H_3_*C*Zn]; IR: ν = 1686 s, 1601 s, 1494 s, 1446 s, 1436 s, 1320 s, 1273 s, 1216 m, 1159 m, 1093 s, 1063 s, 1033 m, 998 s, 958 m, 912 m, 809 s, 661 s, 697 s, 642 m, 597 m, 546 s, 463 s cm^−1^.

### Synthesis of ZnEt_2_ ⊂ [2_3_]_2_

#### Method A

From toluene solution: ZnEt_2_ (0.155 g, 1.25 mmol) was added to a solution of *Bnz*-H (0.242 g, 1.00 mmol) in toluene (4.0 ml) at −78°C. The reaction mixture was allowed to warm to room temperature, and the resulting solution was stirred vigorously for a further 6 hours. Colorless crystals were obtained from slightly concentrated toluene solution at 25°C (0.193 g, isolated yield 36% with reference to entrapped excess of ZnEt_2_).

#### Method B

Recrystallization of **2**_**3**_·0.5PhMe in neat ZnMe_2_: Crystals of **2**_**3**_·0.5PhMe (0.300 g, 0.29 mmol) were dissolved in ZnEt_2_ (1.2 ml). After 10 min, colorless crystals of ZnEt_2_ ⊂ 2_3_ were deposited from the prepared solution at 25°C (0.286 g, isolated yield 94%). The resulting material can be readily dissolved in toluene, benzene, chloroform, dichloroethane, and THF. Elemental analysis (%) calcd for C_106_H_118_O_18_Zn_7_ (2137.59 g mol^−1^): C 59.52, H 5.52, O 13.47, Zn 21.49 (%). Found: C 59.59, H 5.54, O 13.51 (%); ^1^H NMR ([D8]-toluene): δ = 7.63 [br s, 4H, C*H*_(Ar)_], 7.23 [br s, 4H, C*H*_(Ar)_], 7.11 [br s, 2H, C*H*_(Ar)_], 7.10 [s, 0.33H, C*H*_(Ar-Tol)_], 7.10 [s, 0.33H, C*H*_(Ar-Tol)_], 7.02 [s, 0.16H, C*H*_(Ar-Tol)_], 6.98 [s, 0.33H, C*H*_(Ar-Tol)_], 3.24 [br s, 3H, OC*H*_3_], 2.10 [m, 0.5H, C*H*_3(Tol)_], 1.03 [t, 4H, CH_2_C*H*_3_], 0.04 [q, 3H, C*H*_2_CH_3_] ppm; CP-MAS ^13^C NMR: δ = 182.62 [*C*OOMe], 181.67 [*C*OOMe]; 146.15 [*C*H_(Ar)_], 145.39 [*C*H_(Ar)_]; 144.72 [*C*H_(Ar)_]; 143.90 [*C*H_(Ar)_]; 143.52 [*C*H_(Ar)_]; 142.61 [*C*H_(Ar)_]; 127.94 [*C*H_(Ar)_]; 126.74 [*C*H_(Ar)_]; 85.50 [O*C*H_3_]; 84.14 [O*C*H_3_]; 83.56 [O*C*H_3_]; 53.87 [*C*(Ph)_2_(OH)(C)]; 13.63 [ZnCH_2_*C*H_3_]; 11.56 [ZnCH_2_*C*H_3_]; 11.25 [ZnCH_2_*C*H_3_]; 9.05 [ZnCH_2_*C*H_3_]; 3.03 [Zn*C*H_2_CH_3_]; 2.27 [Zn*C*H_2_CH_3_]; −2.22 [Zn*C*H_2_CH_3_]; IR: ν = 1684 s, 1599 s, 1497 s, 1443 s, 1439 s, 1319 s, 1280 s, 1212 m, 1161 m, 1091 s, 1061 s, 1035 m, 992 s, 953 m, 910 m, 812 s, 659 s, 692 s, 641 m, 599 m, 542 s, 461 s cm^−1^.

### Synthesis of ZnMe_2_ ⊂ [3]_2_

Recrystallization of **1**_**3**_·3PhMe or **2**_**3**_·0.5PhMe in a mixture of neat ZnMe_2_/ZnEt_2_: Crystals of **1**_**3**_·3PhMe (0.300 g, 0.24 mmol) or **2**_**3**_·0.5PhMe (0.300 g, 0.29 mmol) were dissolved in the equimolar mixture of ZnMe_2_ and ZnEt_2_ (0.8 mL). After 10 min, colorless crystals of ZnEt_2_ ⊂ [**3**]_2_ were deposited from prepared solution at 25°C [0.203 g, isolated yield 83% (in the case of **1**_**3**_·3PhMe) and 0.261 g, isolated yield 87% (for **2**_**3**_·0.5PhMe)]. The resulting material can be readily dissolved in toluene, benzene, chloroform, dichloroethane, and THF. Elemental analysis (%) calcd for C_102_H_110_O_18_Zn_7_ (2081.48 g mol^−1^): C 58.82, H 5.28, O 13.84, Zn 22.06 (%). Found: C 59.00, H 5.32, O 13.91 (%); ^1^H NMR ([D8]-toluene): δ = 7.64 [br s, 12H, C*H*_(Ar)_], 7.23 [br s, 12H, C*H*_(Ar)_], 7.13 [br s, 6H, C*H*_(Ar)_], 7.10 [s, 0.33H, C*H*_(Ar-Tol)_], 7.10 [s, 0.33H, C*H*_(Ar-Tol)_], 7.02 [s, 0.16H, C*H*_(Ar-Tol)_], 6.98 [s, 0.33H, C*H*_(Ar-Tol)_], 3.15 [br s, 9H, OC*H*_3_], 2.10 [m, 0.5H, C*H*_3(Tol)_], 1.03 [t, 9H, CH_2_C*H*_3_], 0.05 [q, 4H, C*H*_2_CH_3_], −0,79 [s, 6H, *CH_3_*] ppm; CP-MAS ^13^C NMR: δ = 182.72 [*C*OOMe], 181.56 [*C*OOMe]; 146.10 [*C*H_(Ar)_], 145.48 [*C*H_(Ar)_]; 144.80 [*C*H_(Ar)_]; 143.91 [*C*H_(Ar)_]; 143.55 [*C*H_(Ar)_]; 142.78 [*C*H_(Ar)_]; 127.95 [*C*H_(Ar)_]; 126.79 [*C*H_(Ar)_]; 85.54 [O*C*H_3_]; 84.18 [O*C*H_3_]; 83.59 [O*C*H_3_]; 53.87 [*C*(Ph)_2_(OH)(C)]; 13.67 [ZnCH_2_*C*H_3_]; 11.59 [ZnCH_2_*C*H_3_]; 11.35 [ZnCH_2_*C*H_3_]; 9.02 [ZnCH_2_*C*H_3_]; 3.03 [Zn*C*H_2_CH_3_]; 2.32 [Zn*C*H_2_CH_3_]; −2.25 [Zn*C*H_2_CH_3_]; −5.99 [H_3_*C*Zn]; −10.74 [H_3_*C*Zn]; −12.59 [H_3_*C*Zn]; −15.03 [H_3_*C*Zn]; IR: ν = 1686 s, 1599 w, 1494 m, 1446 s, 1436 s, 1376 w, 1315 w, 1271 s, 1214 m, 1185 w, 1162 s, 1092 s, 1062 s, 1032 s, 997 s, 954 m, 910 m, 809 m, 761 s, 726 s, 697 s, 680 s, 640 s, 607 s, 545 s cm^−1^.

### Single-crystal x-ray diffraction

Single crystals of **1**_**3**_, **2**_**3**_, ZnMe_2_ ⊂ [**1**_**3**_]_2_, ZnEt_2_ ⊂ [**2**_**3**_]_2_, and ZnMe_2_ -⊂ [**3**]_2_ were selected under Paratone-N oil, mounted on the nylon loops, and positioned in the cold stream on the diffractometer. Crystal data were collected at 100(2) K with subsequent φ and ω scans on a Nonius Kappa charge-coupled device (CCD) diffractometer with graphite monochromated Mo*K*α radiation (λ = 0.71073 Å) using diffractometer control program “Collect” (*72*). Unit cell parameters and data reduction were processed with Denzo and Scalepak (*73*). The structures were solved by direct methods using SHELXS-97 (*74*). Full-matrix least-squares refinement method against *F*^2^ values was carried out by using the SHELXL-97 program (*75*). All non-hydrogen atoms throughout all five structures were refined with anisotropic displacement parameters. All the hydrogen atoms were placed in calculated positions and refined using a riding model.

Crystallographic data (excluding structure factors) for the structure reported here have been deposited with the Cambridge Crystallographic Data Centre as a supplementary publication. CCDC: 2377894 (**1**_**3**_), 2377895 (**2**_**3**_), 2377897 (ZnMe_2_ ⊂ [**1**_3_]_2_), 2377898 (ZnEt_2_ ⊂ [**2**_**3**_]_2_), 2377899 (ZnMe_2_ ⊂ [**3**]_2_).

### Powder x-ray diffraction

PXRD data were collected with an Empyrean diffractometer (PANalytical) by using Ni-filtered CuKa radiation from a copper sealed tube charged with 40 kV voltage and 40-mA current in Bragg-Brentano geometry with a beam divergence of 1° in the scattering plane.

### TGA analysis

TGA was carried out with a TA Instruments Q600 instrument under an Ar or air flow (flow rate of 100 ml min^−1^) to 600°C at a heating rate of 5°C min^−1^. Open alumina crucibles of 5 mm in diameter were used.

### NMR spectroscopy

The NMR data were obtained on a Varian Inova 500-MHz spectrometer. For details on the diffusion ordered spectroscopy (DOSY) experiments, see section S1.

### FTIR spectroscopy

The infrared spectra were recorded on an FTIR Bruker Tensor II spectrometer using ATR technique.

### High-resolution transmission electron microscope

Nanoparticle samples were drop-cast from MeOH solution onto 300-mesh, holey carbon-coated copper grids (Quantifoil). Afterward, the excess solvent evaporated at room temperature. Nanoparticle samples were imaged using a Cs-corrected scanning transmission electron microscope (HITACHI HD2700, 200 kV). The average size of nanoparticles was calculated using image analyses of a population of 100 particles.

### UV-Vis spectroscopy

Optical absorption (UV-Vis) spectra for ZnO QDs colloidal solution in DMSO were collected on the Hitachi U-2910 spectrophotometer. A standard 3.5-ml quartz cell (Hellma) with a 10-mm path length and transparent on all four sides was used. The PL measurements were carried out with HITACHI Fluorescence Spectrophotometer F-7000. The PLQY for the solid samples were determined with Quantaurus- QY spectrometer (C11347, Hamamatsu Photonics). The PL decays for the solid samples were recorded using Quantaurus-Tau fluorescence lifetime measurement system (C11367, Hamamatsu Photonics) equipped with the LED light source and photon-counting measurement system. The samples were excited at 340 nm, and PL decays were collected at wavelengths corresponding to emission collected on the instrument. The spectra were collected with 10,000 counts at the peak. The data were analyzed by a least squares reconvolution procedure using the software package provided by Hamamatsu. The goodness of fit (fitting order) was determined by χ^2^ value and residuals distribution. When lower than 1.3, the χ^2^ values were taken as appropriate for fitting.

## Supplementary Materials

### This PDF file includes:

Supplementary text S1 to S11 Figs. S1 to S51 Tables S1 to S13 References
