# Supplementary material for: Stabilization toward air and structure determination of pyrophoric ZnR2 compounds via supramolecular encapsulation
Source: Sci Adv. 2025 Apr 25;11(17):eadt7372. doi: 10.1126/sciadv.adt7372 (PMC12024635; doi:10.1126/sciadv.adt7372)
Supplement: Supplementary file 1 — Supplementary text S1 to S11 Figs. S1 to S51 Tables S1 to S13 References [file sciadv.adt7372_sm.pdf]

Science Advances
AAAS

Supplementary Materials for

Stabilization toward air and structure determination of pyrophoric ZnR_2 compounds via supramolecular encapsulation

Kamil Sokołowski *et al.*

Corresponding author: Janusz Lewiński, janusz.lewinski@pw.edu.pl

Sci. Adv. **11**, eadt7372 (2025)
DOI: 10.1126/sciadv.adt7372

This PDF file includes:

Supplementary text S1 to S11
Figs. S1 to S51
Tables S1 to S13
References

Supplementary text

S1. NMR characterization

DOSY NMR analysis

^{1}H DOSY NMR spectra were acquired on a Varian Mercury (300 MHz) spectrometer. The molecular weights (*MW*) of analyzed compounds were estimated utilizing an external calibration curve (ECC) approach with normalized diffusion coefficients exploiting 1,2,3,4-tetraphenylnaphtalene (TPhN) as an internal reference.*(76)* The molecular masses calculated for considered alkylzinc complexes were corrected by a correction factor χ_{cor} for molecules with a high van-der Waals density (MD_w).*(77)* Note, that due to the lack of reference data for [D_8]-toluene, χ_{cor} calculated for C_6D_6 where used instead.

[MeZn(Bnz)]$_x$: Analysis of DOSY ^{1}H NMR spectrum of **1$_3$** in [D_8]-toluene at room temperature indicates the presence of [MeZn(*Bnz*)]$_x$ aggregates with an estimated mass of 664 g·mol^{-1} (figure S4 and table S2). This mass is slightly less than the expected corrected molecular weight (MW_{cor}) for trimeric aggregates (table S1). Furthermore, the signals in the ^{1}H NMR spectrum are significantly broadened, which may indicate an equilibrium between dimeric and trimeric forms. Thus, we perform a similar analysis at -20°C. At the low temperature, the signals split into two sets associated with various forms of complexes with estimated molar masses of 700 and 592 g·mol^{-1}, which fits quite well with the calculated MW_{cor} of trimeric and dimeric aggregates, respectively (figure S6 and table S3). Some deviations may be due to the dynamic equilibria, the presence of other aggregates, as well as the signals overlapping. The estimated ratio of trimeric to dimeric aggregate based on OMe signals is about 5:1.

[EtZn(Bnz)]$_x$: DOSY ^{1}H NMR spectrum of **2$_3$** in [D_8]-toluene at room temperature indicates the presence of [EtZn(*Bnz*)]$_x$ aggregates with an estimated mass of 673 g·mol^{-1} (figure S8 and table S5), which is between calculated MW_{cor} for trimeric and dimeric forms (table S4). Spectra at -20°C shows the presence of two sets of signals associated with various aggregates with estimated molar masses of 805 and 505 g·mol^{-1}, which fits quite well with the calculated MW_{cor} of trimeric and dimeric aggregates, respectively (figure S9 and table S6). Some deviations may be due to the dynamic equilibria, the presence of other aggregates, or the signals overlapping. The estimated ratio of trimeric to dimeric aggregate based on OMe signals is about 2:1.

S2. Crystal structure characterization

Crystal structure description

The common feature of the obtained crystalline materials is the presence of isostructural trinuclear alkylzinc molecules. In the case of complexes **1$_3$** and **2$_3$** they are composed of three [MeZn(*Bnz*)] or [EtZn(*Bnz*)] units, respectively (figures S14 and S16), while complex **3** comprises one [MeZn(*Bnz*)] and two [EtZn(*Bnz*)] moieties (figure S24). In all of the molecular structures, the monomeric units are bridged up by alkoxide oxygen atoms of the chelating *Bnz* ligands resulting in the formation of central six-membered [Zn(μ_2-O)]$_3$ rings adopting a pseudo-boat conformation. The alkyl groups and *Bnz* backbones in monomeric units are located on opposite sides of the central ring and in each structure one of the ligands is on the opposite side of the Zn_3O_3 ring than two others. The overall shape of the alkylzinc aggregates is quasi-spherical with three rounded hollow sides of the baseball glove-like fashion (named further as nano-pockets, see main text), which contain alkylzinc moieties surrounded by phenyl and ester groups from *Bnz* ligands.

1$_3$ and **2$_3$** crystalized from toluene solution as solvates **1$_3$**·3PhMe and **2$_3$**·0.5PhMe, respectively, with different packing of the trimeric molecules. The entrapped solvent molecules are localized in a nonspecific manner in closed voids between [RZn(*Bnz*)]$_3$ molecules (figures

S15 and S17). In contrast, the crystal packing of the supramolecular capsules in $ZnMe_2\subset[\mathbf{1}_3]_2$, $ZnEt_2\subset[\mathbf{2}_3]_2$, and $ZnMe_2\subset[\mathbf{3}]_2$ is almost identical (figures S19, S21, and S25). The capsules form a 3D lattice in which $ZnMe_2$ or $ZnEt_2$ molecules are located in the *ab* planes separated from each other by *ca.* 17.4 Å, 17.8 Å, and 17.4 Å, respectively. The comparison between the crystal structure of $ZnMe_2\subset[\mathbf{1}_3]_2$, $ZnEt_2\subset[\mathbf{2}_3]_2$ and $ZnMe_2\subset[\mathbf{3}]_2$ revealed that the character of alkyl group in both the host molecules and the dialkylzinc guests did not significantly affect the molecular and supramolecular architecture of the resulting crystals (*i.e.* only small changes of cell lengths and volume are observed, and the *ab* planes are comparably spaced) (figure S23).

S3. Hirshfeld surface analysis

Hirshfeld surface analysis was carried out for the guest and host molecules of the crystal structures of $ZnMe_2\subset[\mathbf{1}_3]_2$, $ZnEt_2\subset[\mathbf{2}_3]_2$, and $ZnMe_2\subset[\mathbf{3}]_2$ using the program CrystalExplorer version 17.5.*(78)* Distances d_e and d_i are defined as the distances from the Hirshfeld surface to the nearest nucleus outside and inside the surface, respectively. The colors red, green, and blue on the surfaces mapped by d_e and d_i represent short, intermediate, and long distance regions, respectively.*(79)* Electrostatic potential (ESP) maps were calculated using Tonto software implemented into CrystalExplorer at the B3LYP/6-31G(d,p) level based on molecular geometries directly from the crystal structures of $ZnMe_2\subset[1_3]_2$, $ZnEt_2\subset[2_3]_2$, and $ZnMe_2\subset[3]_2$. The molecular ESP is mapped on Hirshfeld surfaces over the range -0.05 au (red), through 0 (white), to +0.05 au (blue); 1 au = 2625.5 kJ·mol^{-1} per unit charge.*(80)*

The fragment patch analysis demonstrates that the environment of guest ZnR_2 molecules in the host-guest systems is dominated by the dyad of encapsulating trimeric host molecules; the respective intermolecular contacts constitute 77%, 73%, and 83% of the guest's Hirshfeld surface in $ZnMe_2\subset[\mathbf{1}_3]_2$, $ZnEt_2\subset[\mathbf{2}_3]_2$, and $ZnMe_2\subset[\mathbf{3}]_2$ supramolecular systems, respectively (figure S29). The d_e maps show relatively close C-H···Zn contacts between the alkyl groups of host trimers and the Zn atoms of encapsulated ZnR_2 molecules (figures S26-S28). Notably, the areas corresponding to these interactions are larger in the case of $ZnMe_2$ in comparison to $ZnEt_2$ guests (11.9%, 11.6%, and 6.0% of the Hirshfeld surface for $ZnMe_2\subset[\mathbf{3}]_2$, $ZnMe_2\subset[\mathbf{1}_3]_2$ and $ZnEt_2\subset[\mathbf{2}_3]_2$, respectively). The maps of ESP on Hirshfeld surfaces show that host-guest systems are formed with electrostatic complementarity (figure S30). The guest ZnR_2 molecules accumulate considerable positive ESP (reaching 0.06-0.08 au) around Zn centers and weaker negative ESP on the polarized alkyl groups (ca. -0.01 to -0.02 au). In turn, the positive ESP on host molecules is localized around the ester O-bonded Me groups (ca. 0.05 au) while the negative ESP is associated with the polarized Zn-R groups of the antenna moieties (ca. -0.05 au). The supramolecular capsules are assembled that match the electropositive regions around the Zn center of ZnR_2 molecules with electronegative regions of R-Zn groups within the nano-pockets of the hosts (Figure 2E), which likely act as a specific recognition system imitating the environment observed in the crystal structure of neat ZnR_2. Furthermore, the electronegative regions around the alkyl groups of ZnR_2 molecules are directed toward the electropositive OMe groups within the nano-cavity environment.

S4. Analysis of the structures of ZnR_2 molecules within confinement space

The entrapped $ZnMe_2$ molecules in $ZnMe_2$⊂[**1₃**]₂ and $ZnMe_2$⊂[**3**]$_2$ are entirely linear and their Zn-C bond lengths are significantly longer (1.952 Å and 1.979 Å, respectively) in comparison to the corresponding value obtained from the *ab initio* calculations (1.930 Å) (figure S31). In the case of $ZnEt_2$⊂[$\mathbf{2_3}$]$_2$, both ethyl groups are in a *trans* position, which is in good agreement with *ab initio* studies in the gas phase. This is however in sharp contrast to the solid-state structure of $ZnEt_2$, where molecules slightly deviate from linearity (C-Zn-C angle, 176.2(4)°) and the ethyl groups of $ZnEt_2$ have *cis* conformation (Zn-C-C 116.7(6)°). The bond length observed for the encapsulated $ZnEt_2$ (1.944 Å) is much closer to the calculated value (1.944 Å) in comparison to its methyl analogues. Within the nano-cavities the entrapped ZnR_2 molecules interact with host trimers by symmetrical C-H•••Zn non-covalent interactions formed between the antenna Zn-R moieties in the interior of supramolecular containers and the Zn atoms of the encapsulated ZnR_2 molecule resemble the crystal environment of ZnR_2 molecules in their condensed forms.

S5. Studies on the stability of ZnR_2-filled capsules toward air

Analysis of the decomposition of encapsulated ZnR_2 molecules over the course of air exposure of the capsules utilizing ^1H NMR spectroscopy is hindered by the overlap of the Zn-R signals from ZnR_2 and [RZn(Bnz)]$_n$ species. ^1H NMR spectra collected for $ZnEt_2$⊂[$\mathbf{2_3}$]$_2$ after prolonged exposure to air of the solid-state sample reveal a slow decrease in the intensity of Zn-CH_2 resonances over time (figure S34). The decrease is initially faster, with the intensity ratio between OMe and Zn–CH_2 signals reaching the value calculated for the pure complex $\mathbf{2_3}$ within 60–90 min. Thereafter, the decline slows significantly, and the OMe and Zn–CH_2 signal ratio oscillates slightly below the value calculated for the pure complex $\mathbf{2_3}$. The spectra indicate the presence of Zn–Et groups even after 4 hours of exposure.

S6. TGA analysis

The TGA profiles for $ZnMe_2$⊂[$\mathbf{1_3}$]$_2$ and $ZnEt_2$⊂[$\mathbf{2_3}$]$_2$ show mass decreases of 4.8% and 5.9%, respectively, below 150°C (figures S37 and S38). These values align well with the calculated theoretical mass content of the encapsulated ZnR_2 guests within the samples, which are 4.7% and 5.8%, respectively.

S7. Temperature-controlled release experiments

A crystalline sample of $ZnEt_2$⊂$\mathbf{2_3}$ (1g) was loaded under air into a distillation setup and heated to 120°C under vacuum. The colourless liquid was collected within 20 min in a Schlenk tube cooled in a liquid nitrogen bath (yield 57 mg, 99%). The purity of the released $ZnEt_2$ was confirmed by ^1H NMR spectroscopy (figure S39). The residual white powder was identified as pure $\mathbf{2_3}$ in a new crystal phase; corroborated by ^1H NMR, PXRD, FTIR, and elemental analysis (figures S40 and S41).

Recovery of $ZnEt_2$ after air exposure. In three separate experiments, crystalline samples of $ZnEt_2$⊂$\mathbf{2_3}$ (200 mg) were exposed to air for 10, 30 and 60 min (22°C, RH 15%), respectively. Then, the samples were loaded into a distillation setup and heated to 120°C under vacuum. The colourless liquid was collected within 20 min in a Schlenk tube cooled in a liquid nitrogen bath. The amount of recovered $ZnEt_2$ was determined by ^1H NMR spectroscopy using 1,2,3,4-tetraphenylnaphtalene (TPhN, 4 mg/ml in C_6D_6, 0.6 ml) as an internal standard, yielding 11.7 mg (98%), 8.0 mg (67%) and 3.8 mg (32%) after 10-, 30- and 60-min exposition, respectively (figure S42).

S8. Experiments on the application of the $ZnEt_2$-loaded capsules as initiator for the radical addition of THF to imines.

In a control experiment, the effectiveness of $ZnEt_2\subset[\mathbf{2_3}]_2$ as an initiator for the radical addition of THF to imines was compared with a very efficient model radical initiator $[EtZn(OOCPh)]_n$, recently developed by our group.(*67*) 4-methoxy-N-(4-methoxybenzylidene)aniline was selected as the model imine. After 16 h of the reaction, conversion of the imine to the respective THF adduct, was 80% for $[EtZn(OOCPh)]_n$ and only 5% for $ZnEt_2\subset[\mathbf{2_3}]_2$, based on ^{1}H NMR analysis (figure S43).

A control experiment involving [$ZnEt_2\subset[\mathbf{2_3}]_2$: $ZnEt_2\subset[\mathbf{2_3}]_2$ (205 mg, 0.1 mmol) was dissolved in 4 ml of dry THF An excess of dry air was introduced to a stirred THF (4 mL) solution of and 4-methoxy-N-(4-methoxybenzylidene)aniline (6 mg, 0.025 mmol) at 20°C. The reaction was stirred 16 h, followed by hydrolysis with a saturated solution of $NaHCO_3$. Products were extracted with ethyl acetate (3 x 3 ml), dried over anhydrous $MgSO_4$, and characterized using ^{1}H NMR spectroscopy.

A control experiment involving $[EtZn(OOCPh)]_n$: In a Schlenk flask under inert N_2 atmosphere, benzoic acid (12.2 mg, 0.1 mmol) was dissolved in 4 ml of dry THF. Then, a solution of $ZnEt_2$ (0.1 ml, 1M in hexane) was added at -78°C. The reaction mixture was allowed to warm to room temperature and the resulting solution was stirred vigorously for a further 4 h. 4-methoxy-N-(4-methoxybenzylidene)aniline (6 mg, 0.025 mmol) was added to the as prepared THF solution of $[EtZn(OOCPh)]_n$ radical initiator followed by the introduction of dry air. The reaction was stirred 16 h, followed by hydrolysis with a saturated solution of $NaHCO_3$. Products were extracted with ethyl acetate (3 x 3 ml), dried over anhydrous $MgSO_4$, and characterized using ^{1}H NMR spectroscopy.

S9. Experiments on the utilization of the $ZnEt_2$-loaded capsules as precursors of ZnO NCs

A solution of $ZnEt_2\subset[\mathbf{2_3}]_2$ (615 mg, 0.3 mmol) in THF (5 ml) was exposed to air and stirred at room temperature for 4 days. Then, 2 ml of hexane was added, and the resulting suspension was separated in a centrifuge, followed by two washes with a THF/hexane (v/v 5:2) mixture. The final product was dried under vacuum, leading to stable ZnO quantum dots capped by *Bnz* ligands (ZnO-*Bnz*), which form stable colloidal solutions in MeOH and DMSO. The new nanomaterial was characterized by PXRD, HR-TEM, TGA, FTIR, and UV/Vis spectroscopy.

The HRTEM images show an irregular shape of the obtained nanocrystals, with an average size of 6.6±1.8 nm (figure S45). This value is consistent with the average size of 6.5 nm estimated from powder pattern analysis (figure S44). The TGA-DSC plot reveals a two-step mass loss, occurring in the temperature ranges of 90–210°C (9.7%) and 260–500°C (12.5%) (figure S46). This observation suggests the presence of bimodal stabilization of the surface of NCs, likely involving monoanionic X-type *Bnz* and neutral L-type *Bnz*-H ligands. The total weight loss attributed to organic components of ZnO-*Bnz* was 22.2%. The FTIR spectrum of ZnO-*Bnz* (figure S47) displays an intense band with a maximum at about 405 cm^{-1} associated with the ZnO core, along with a series of signals related to the organic ligands on its surface. In particular, there are several signals in the range between 1515-1640 cm^{-1} merging into a broad band, likely associated with the C=O vibrations of various forms of ligands. These bands are significantly bathochromicaly shifted compared to the corresponding signals in both $ZnEt_2\subset[\mathbf{2_3}]$ capsules (1685 cm^{-1}) and *Bnz*-H proligand (1721 cm^{-1}). This shift suggests a different coordination mode of *Bnz* ligands on the ZnO surface than that observed in trimeric molecules of $\mathbf{2_3}$. Furthermore, the FTIR spectrum of ZnO-*Bnz* shows an extensive band in the range of 3100-3600 cm^{-1}, which may indicate the presence of various OH groups on the surface

of nanocrystals likely associated with protonated *Bnz*-H and/or water molecules interacting with the surface via coordination or hydrogen bonds. The ZnO-*Bnz* quantum dots exhibit yellow luminescence under UV light (figure S48), showing a broad emission peak with a maximum at λ_{em} = 575 nm (λ_{ex} = 310 nm) (figure S49). The photoluminescence quantum yield was 9.2%. The *Bnz*-coated ZnO NCs exhibit relatively long PL charge recombination with four lifetime components (fitted with multiexponential function) (figure S50). The major contribution are three components with a decay time of about 37.0 ns (34.9%), 349.5 ns (30.0%), and 1.4 µs (31.0%) and the fourth minor component is 5.3 µs (4.1%).

S10. Pore size distribution analysis

The pore size distributions (PSDs) were calculated using the method of Gelb and Gubbins,*(81)* where the largest sphere that can fit in a random point within a structure without overlapping the van der Waals surface of the framework is recorded for a large number of random points. PSD of [$\mathbf{1}_3$], [$\mathbf{2}_3$]$_2$, and [**3**]$_2$ containers reveal cavity sizes of 3.55, 3.45, and 3.65 Å, respectively (figure S51). When encapsulating a ZnR_2 guest molecule, these cavities are saturated.

S11. Binding energy calculation

We estimated the binding energies (BE) of $ZnMe_2$ and $ZnEt_2$ on [$\mathbf{1}_3$]$_2$, [$\mathbf{2}_3$]$_2$, and [**3**]$_2$ (table S12 and S13 shows the total energy values) according to equation [1]:

$$BE = E(ZnR_2 \subset Host) - E(ZnR_2) - E(Host) \quad [1]$$

At this point, it is important to highlight that BE were obtained using the periodic boundary condition, which takes into account the interaction between the capsules in the crystal lattice. Molecular simulations using the Forcite module of Materials Studio*(82)* were used to calculate the total and binding energy of the molecules and the systems. Structures for the simulations were obtained from experimental, X-ray data without any further optimization. The bonded and the short range (van der Waals) interactions between the atoms were modelled using the Universal Force Field (UFF),*(83)* with a cut-off distance of 18.5 Å. Coulombic interactions were modelled by placing partial charges on the framework atoms. The partial charges were calculated using a charge equilibration method included in Materials Studio, and the long-range electrostatic interactions were calculated using the Ewald summation method.

Classical molecular simulation shows no significant difference between cis conformation obtained from the crystalline structure of pure $ZnEt_2$ and the trans configuration obtained from the adsorbed molecules (table S12).

Supplementary figures

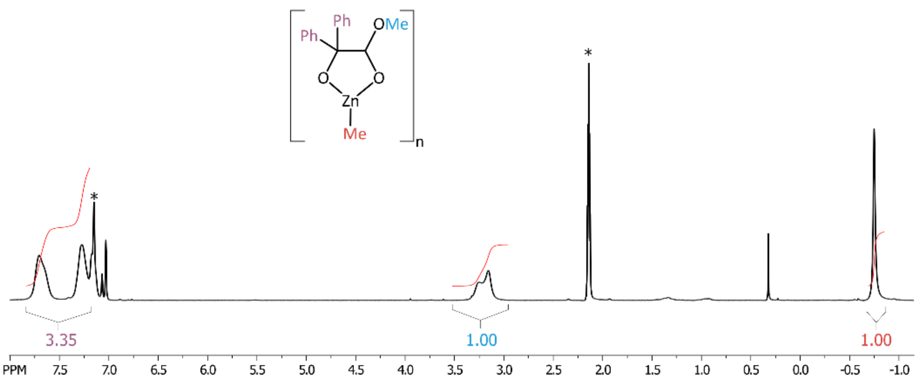

Fig. S1. NMR spectrum of $\mathbf{1}_3$. ^{1}H NMR spectrum of $\mathbf{1}_3$·3MePh in [D_8]-toluene. The asterisks denote signals from [D_8]-toluene.

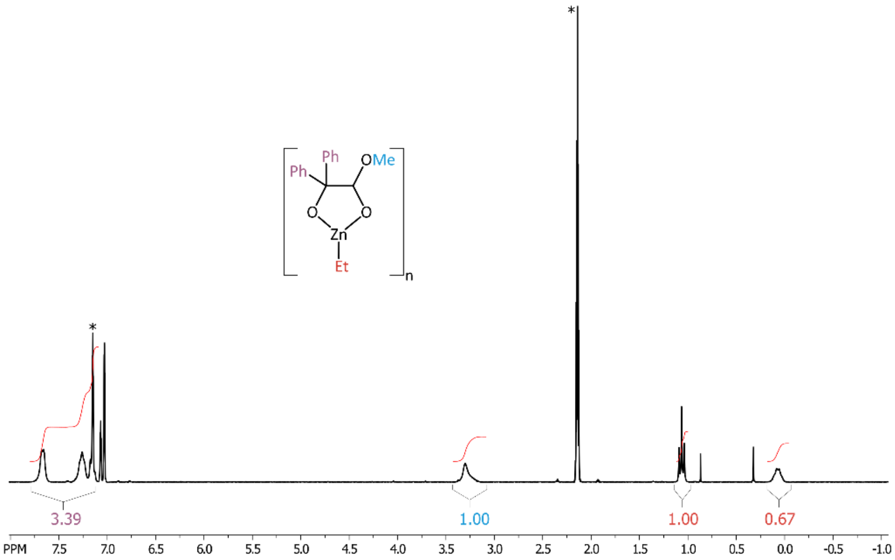

Fig. S2. NMR spectrum of $\mathbf{2_3}$. ^{1}H NMR spectrum of $\mathbf{2_3}$·0.5MePh in [D$_8$]-toluene. The asterisks denote signals from [D$_8$]-toluene.

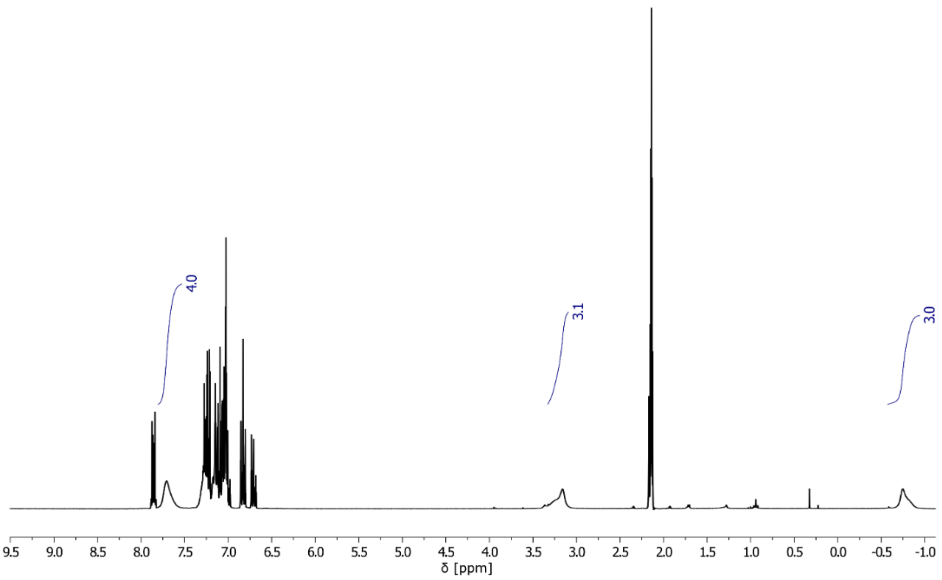

Fig. S3. Room temperature NMR spectrum of $\mathbf{1_3}$ with an internal standard. ^{1}H NMR spectrum of $\mathbf{1_3}$ with the addition of TPhN in [D$_8$]-toluene at 25°C.

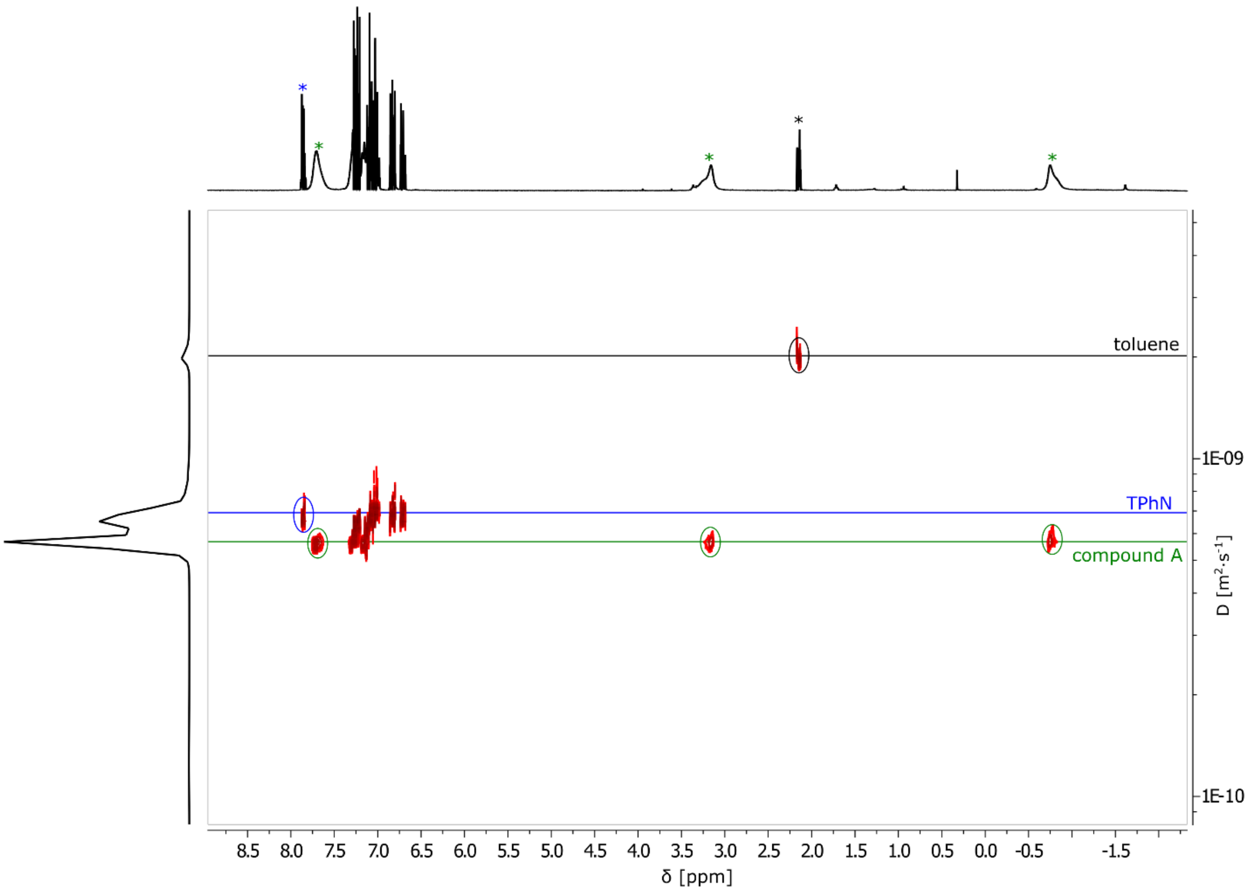

Fig. S4. Room temperature DOSY NMR spectrum of 1$_3$ with an internal standard. 2D DOSY ^{1}H NMR spectrum of **1$_3$** with the addition of TPhN in [D$_8$]-toluene at 25°C (signals used to estimate *MW* are marked with asterisks).

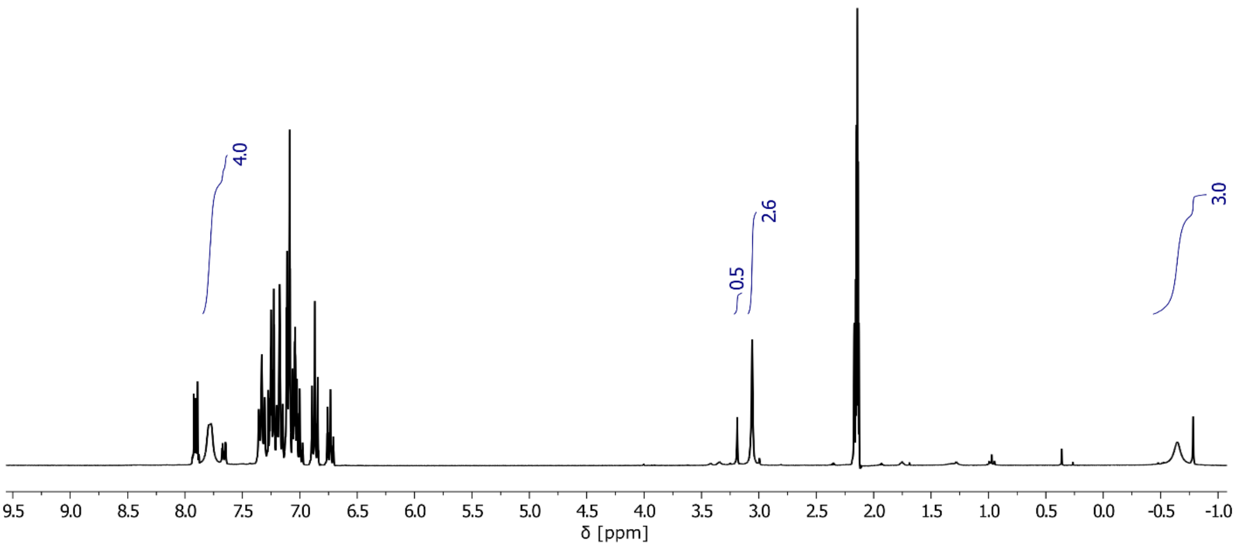

Fig. S5. Low temperature NMR spectrum of 1$_3$ with an internal standard. ^{1}H NMR spectrum of **1$_3$** with the addition of TPhN in [D$_8$]-toluene at -20°C.

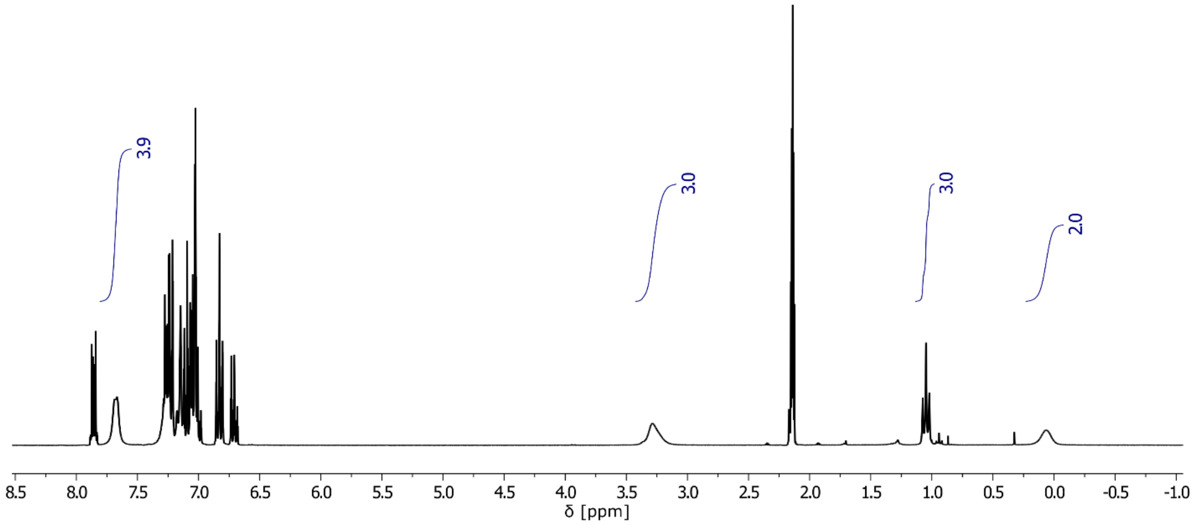

Fig. S7. Room temperature NMR spectrum of 1$_3$ with an internal standard. ^{1}H NMR spectrum of **2**$_3$ with the addition of TPhN in [D$_8$]-toluene at 25°C.

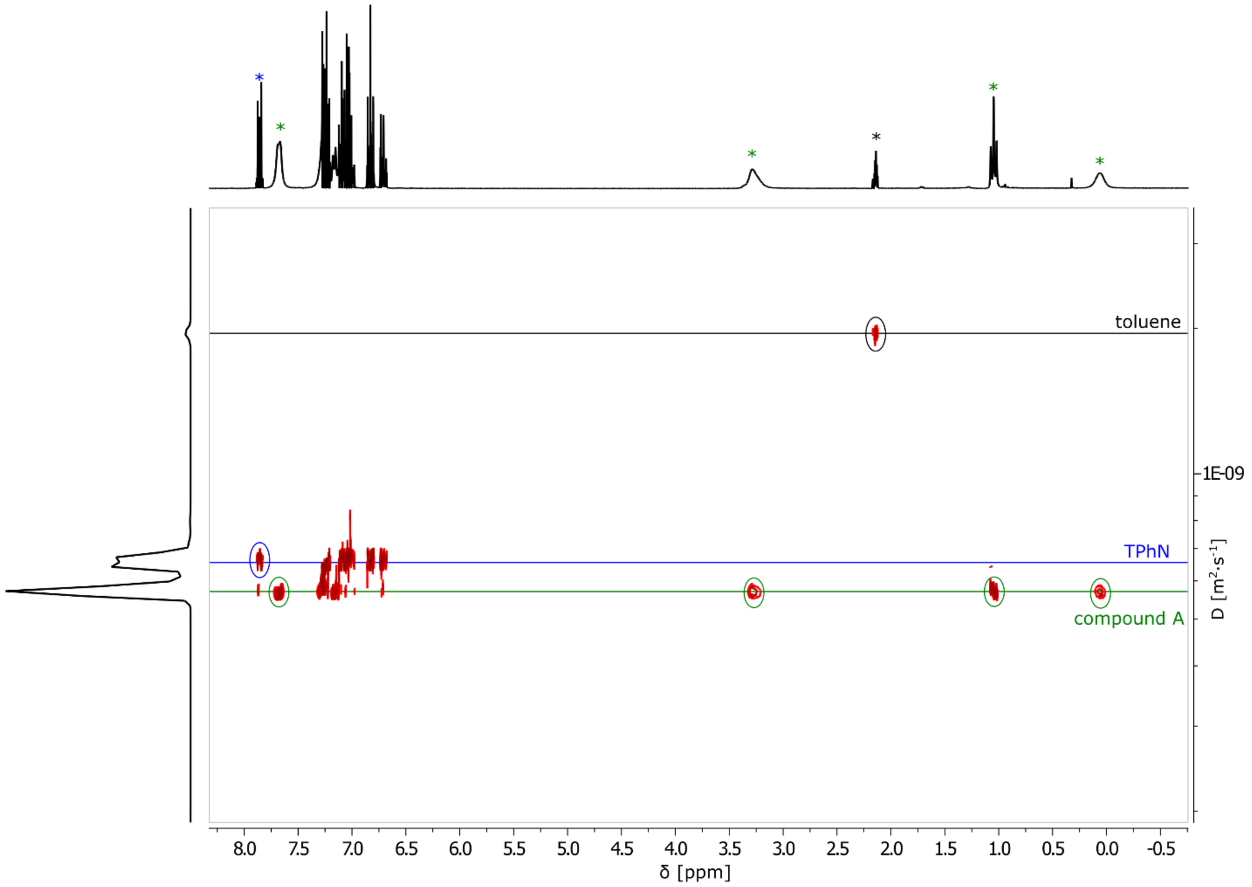

Fig. S8. Room temperature DOSY NMR spectrum of 2$_3$ with an internal standard. 2D DOSY ^{1}H NMR spectrum of **2**$_3$ with the addition of TPhN in [D$_8$]-toluene at 25°C (signals used to estimate *MW* are marked with asterisks).

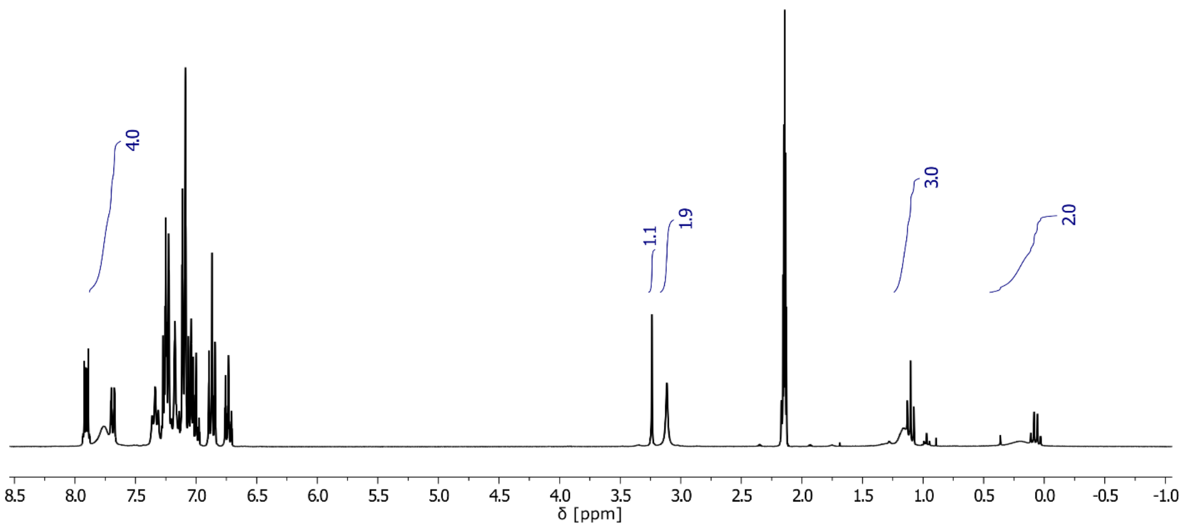

Fig. S9. Low temperature NMR spectrum of $\mathbf{1_3}$ with an internal standard. ^{1}H NMR spectrum of $\mathbf{2_3}$ with the addition of TPhN in [D$_8$]-toluene at -20°C.

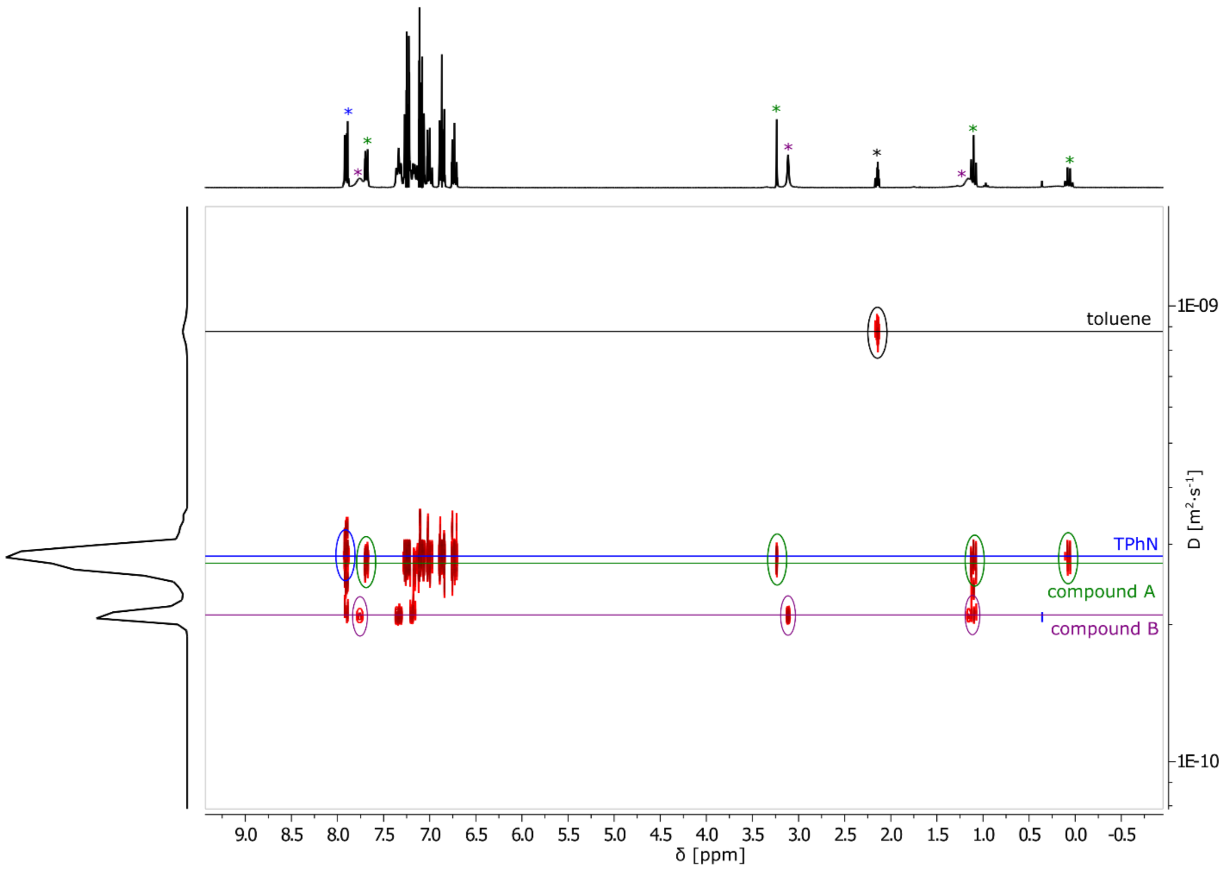

Fig. S10. Low temperature DOSY NMR spectrum of $\mathbf{1_3}$ with an internal standard. 2D DOSY ^{1}H NMR spectrum of $\mathbf{2_3}$ with the addition of TPhN in [D$_8$]-toluene at -20°C (signals used to estimate *MW* are marked with asterisks).

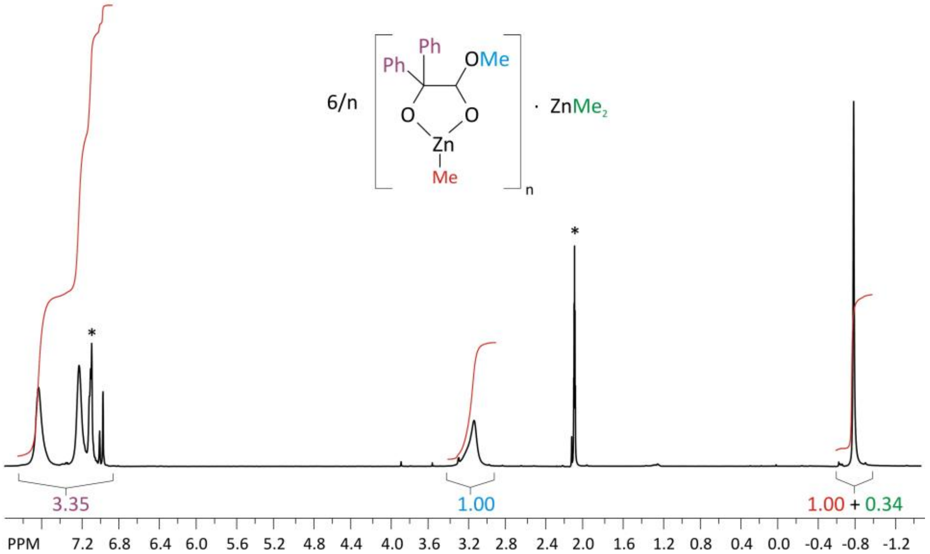

Fig. S11. NMR spectrum of $ZnMe_2$⊂[1_3]$_2$. ^{1}H NMR spectrum of $ZnMe_2$⊂[**1**$_3$]$_2$ in [D_8]-toluene. The asterisks denote signals from [D_8]-toluene.

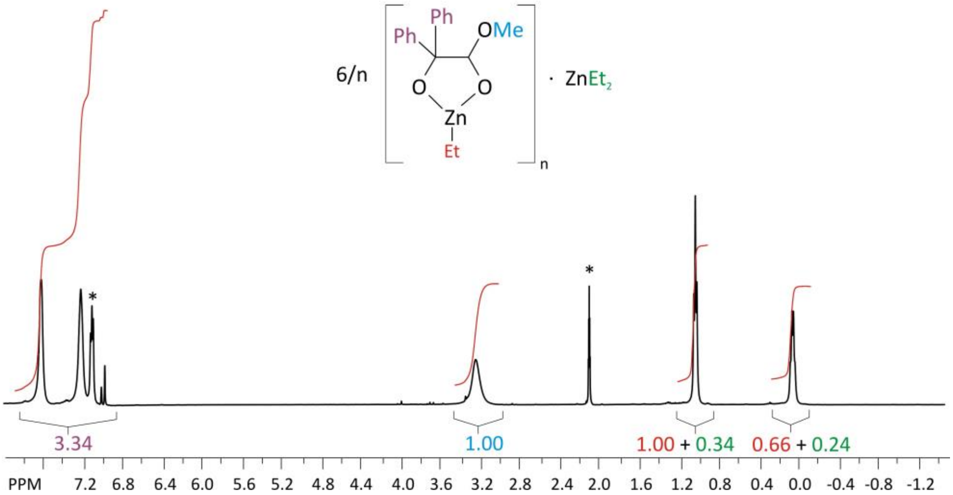

Fig. S12. NMR spectrum of $ZnEt_2$⊂[2_3]$_2$. ^{1}H NMR spectrum of $ZnEt_2$⊂[**2**$_3$]$_2$ in [D_8]-toluene. The asterisks denote signals from [D_8]-toluene.

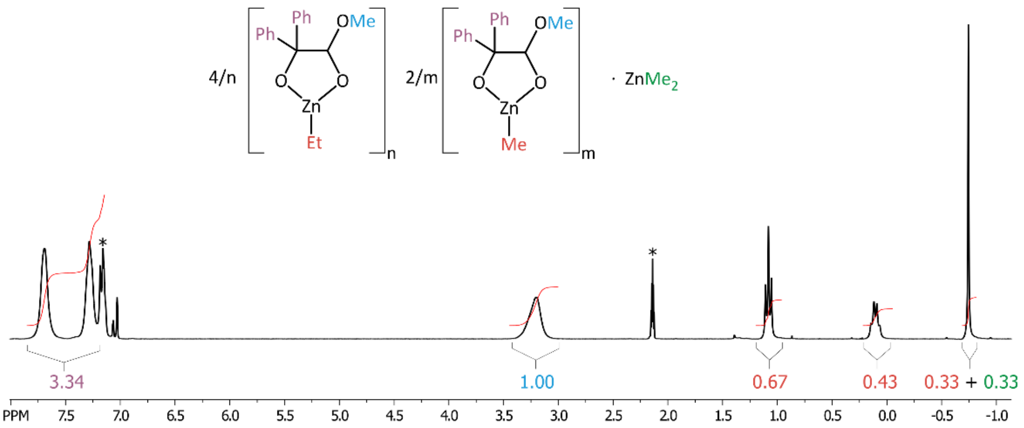

Fig. S13. NMR spectrum of $ZnMe_2 \subset [3]_2$. ^1H NMR spectrum of $ZnMe_2 \subset [\mathbf{3}]_2$ in $[D_8]$-toluene. The asterisks denote signals from $[D_8]$-toluene.

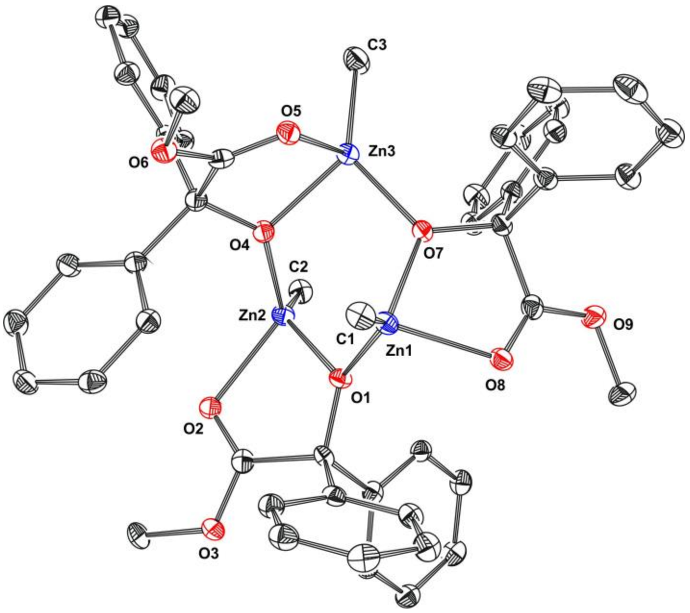

Fig. S14. Molecular structure of $\mathbf{1}_3$. Molecular structure of $\mathbf{1}_3$ with thermal ellipsoids set at 30% probability. Hydrogen atoms have been omitted for clarity.

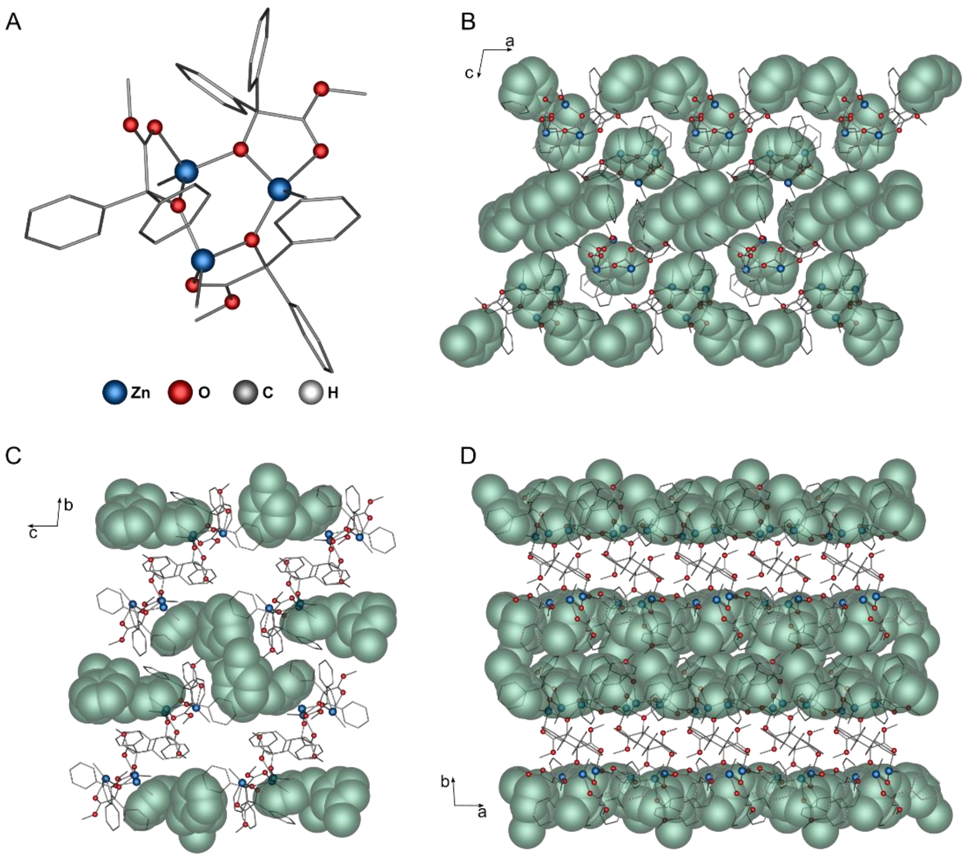

Fig. S15. Crystal structure of 1_3·3PhMe. Molecular structure of 1_3 (A) and supramolecular structure of 1_3·3PhMe with solvent molecules marked on green (space-filling model); view along *b*, *a*, *c* crystal axis for B, C, D, respectively (the hydrogen atoms are omitted for clarity).

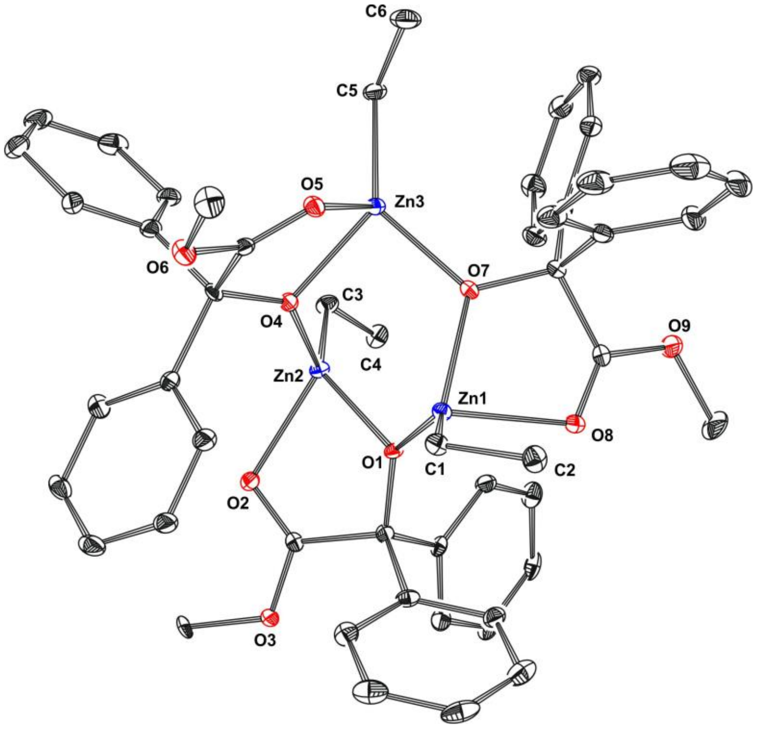

Fig. S16. Molecular structure of $\mathbf{2_3}$. Molecular structure of $\mathbf{2_3}$ with thermal ellipsoids set at 30% probability. Hydrogen atoms have been omitted for clarity.

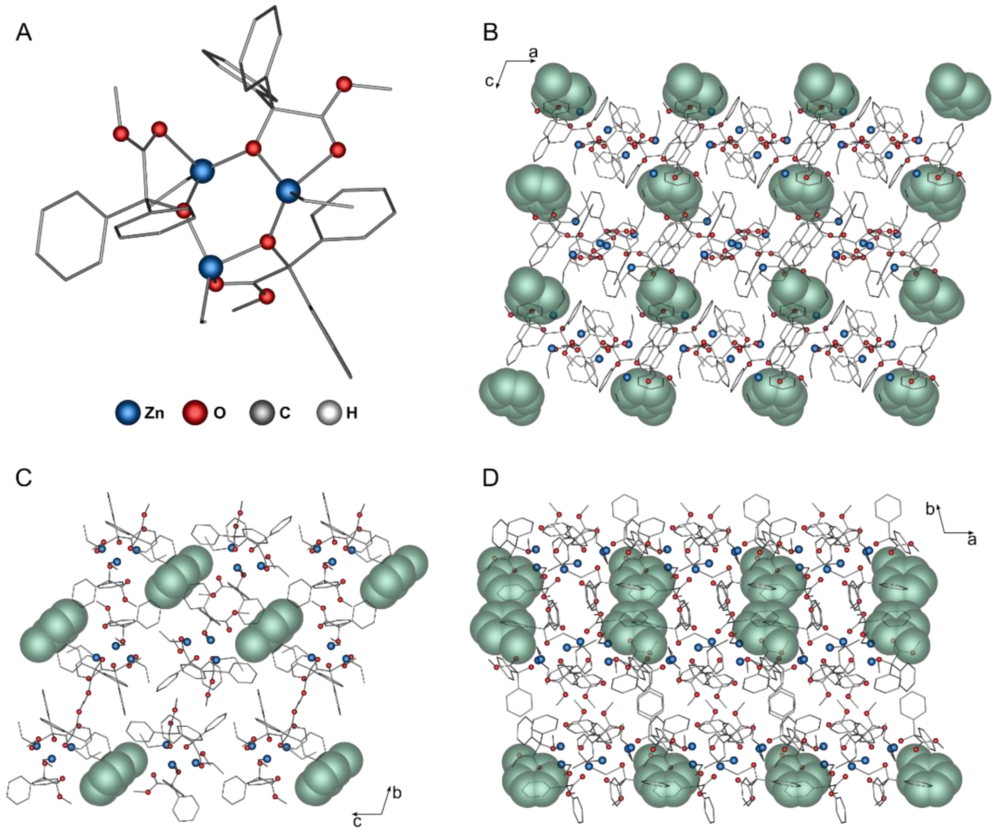

Fig. S17. Crystal structure of 2_3·0.5PhMe. Molecular structure of **2_3** (A) and supramolecular structure of **2_3**·0.5PhMe with solvent molecules marked on green (space-filling model); view along *b*, *a*, *c* crystal axis for B, C, D, respectively (the hydrogen atoms are omitted for clarity).

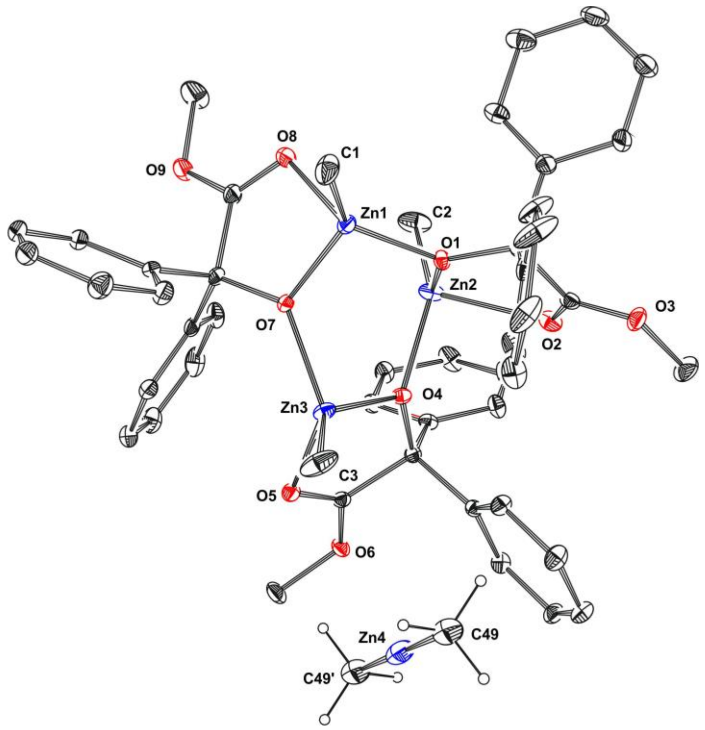

Fig. S18. Molecular structure of ZnMe$_2$⊂[1_3]$_2$. Molecular structure of ZnMe$_2$⊂[**1_3**]$_2$ with thermal ellipsoids set at 30% probability. Hydrogen atoms have been omitted for clarity. Operators for generated equivalent atoms: (x+2,-y+2,-z+1).

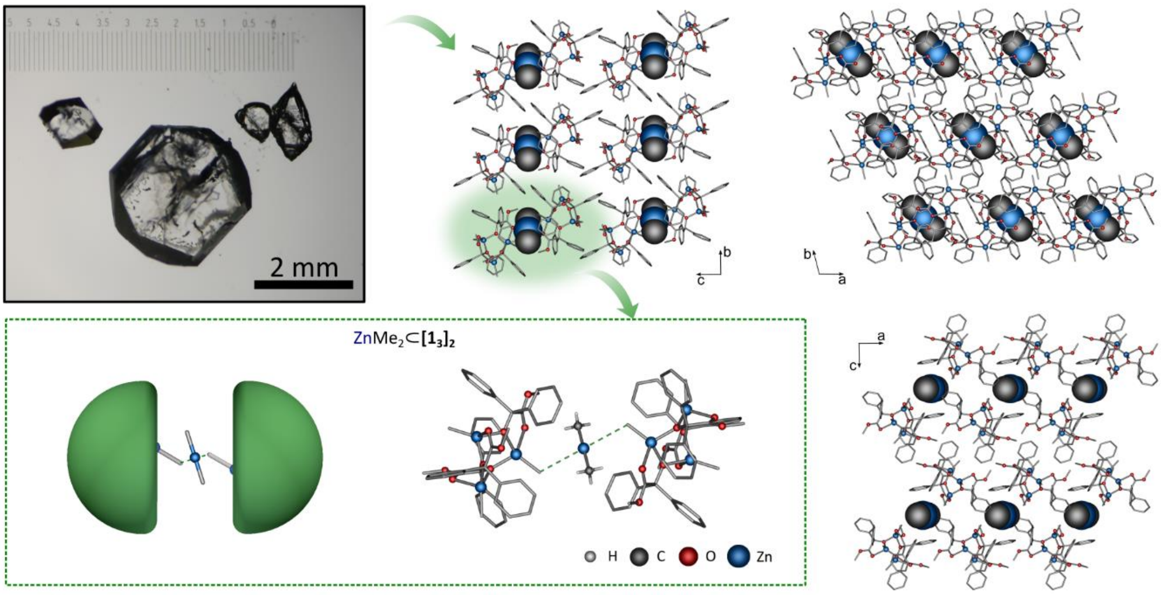

Fig. S19. Crystal structure of $ZnMe_2$⊂[1_3]$_2$. Image of $ZnMe_2$⊂[1_3]$_2$ crystals and structure and supramolecular packing of capsule $ZnMe_2$⊂[1_3]$_2$ (E).

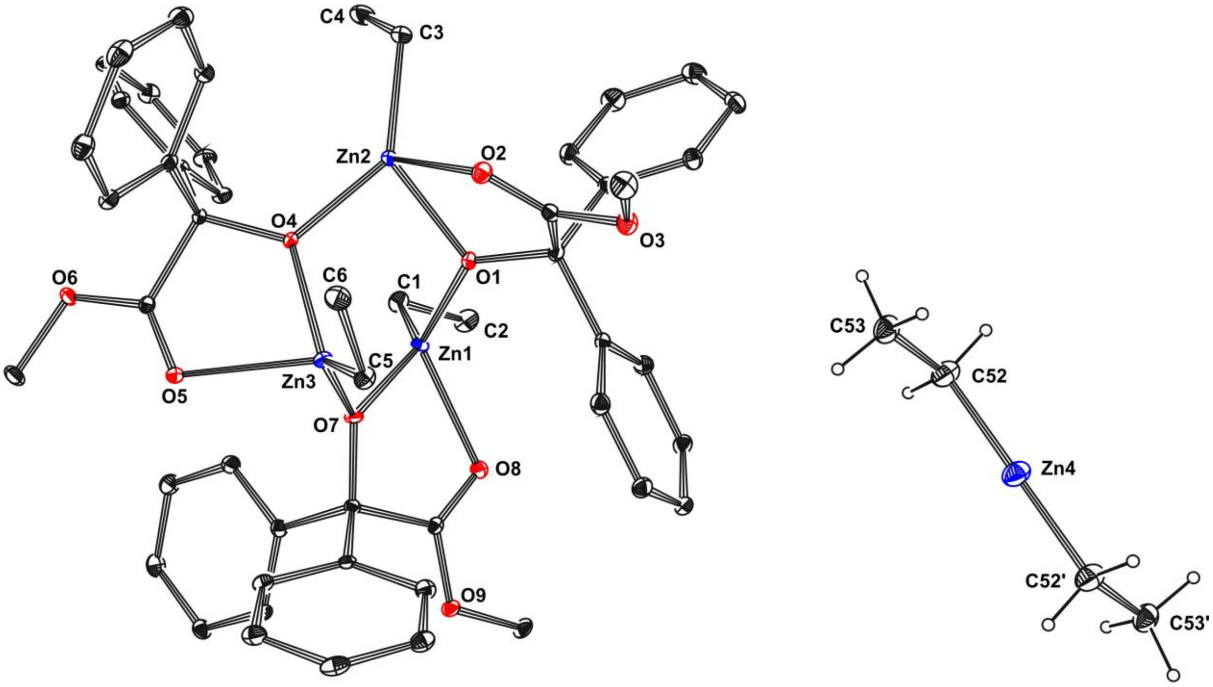

Fig. S20. Molecular structure of $ZnEt_2$⊂[2_3]$_2$. Molecular structure of $ZnEt_2$⊂[2_3]$_2$ with thermal ellipsoids set at 30% probability. Hydrogen atoms have been omitted for clarity. Operators for generated equivalent atoms: (-x,-y+2,-z).

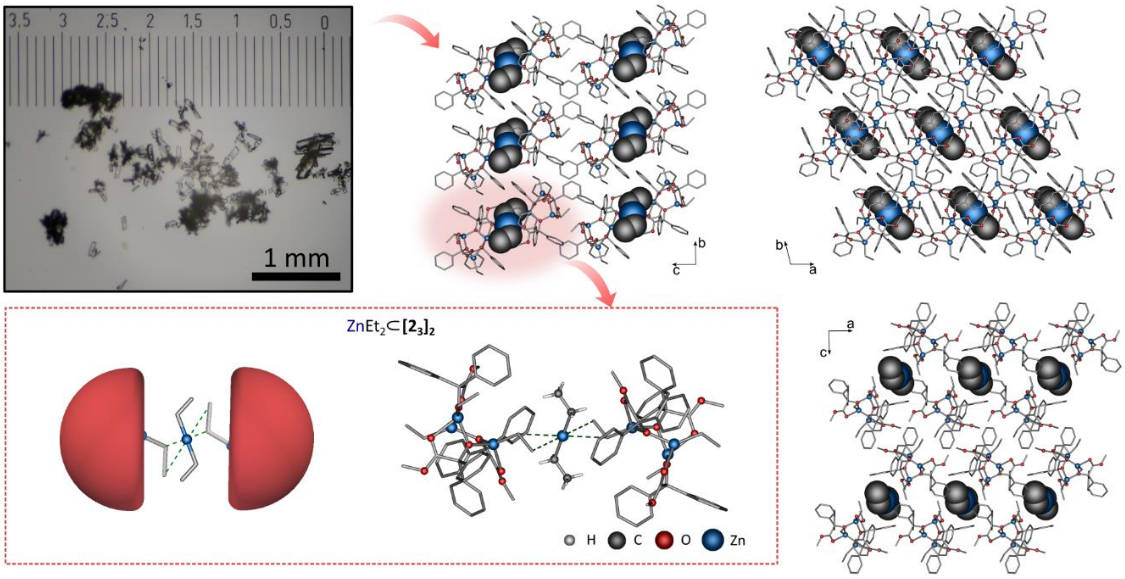

Fig. S21. Crystal structure of $ZnEt_2$⊂[2_3]$_2$. Image of $ZnEt_2$⊂[**2_3**]$_2$ crystals and structure and supramolecular packing of capsule $ZnEt_2$⊂[**2_3**]$_2$ (E).

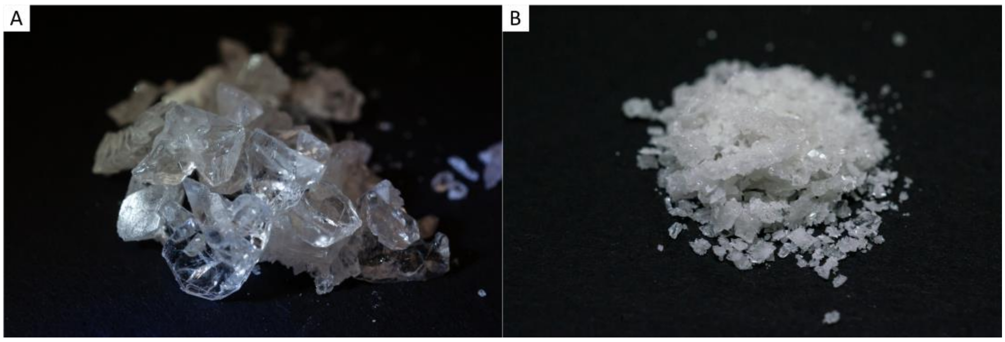

Fig. S22. Crystals of ZnR_2-filled capsules. Photography of bulk samples of $ZnMe_2$⊂[**1_3**]$_2$ (A) and $ZnEt_2$⊂[**2_3**]$_2$ (B) under air.

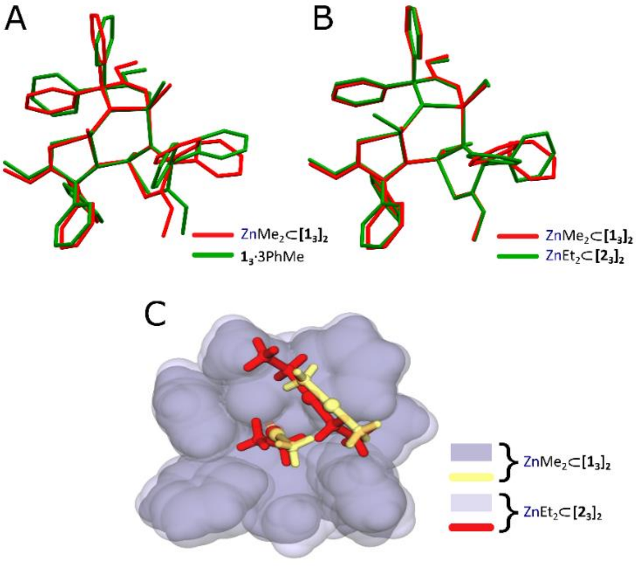

Fig. S23. Conformation of encapsulating components and guest molecules. Structural comparison between: molecules of **1_3** in **1_3**·3PhMe and $ZnMe_2$⊂[**1_3**]$_2$ (A), host molecules in $ZnMe_2$⊂[**1_3**]$_2$ and $ZnEt_2$⊂[**2_3**]$_2$ (B), and nano-pockets in $ZnMe_2$⊂[**1_3**]$_2$ and in $ZnEt_2$⊂[**2_3**]$_2$ (C).

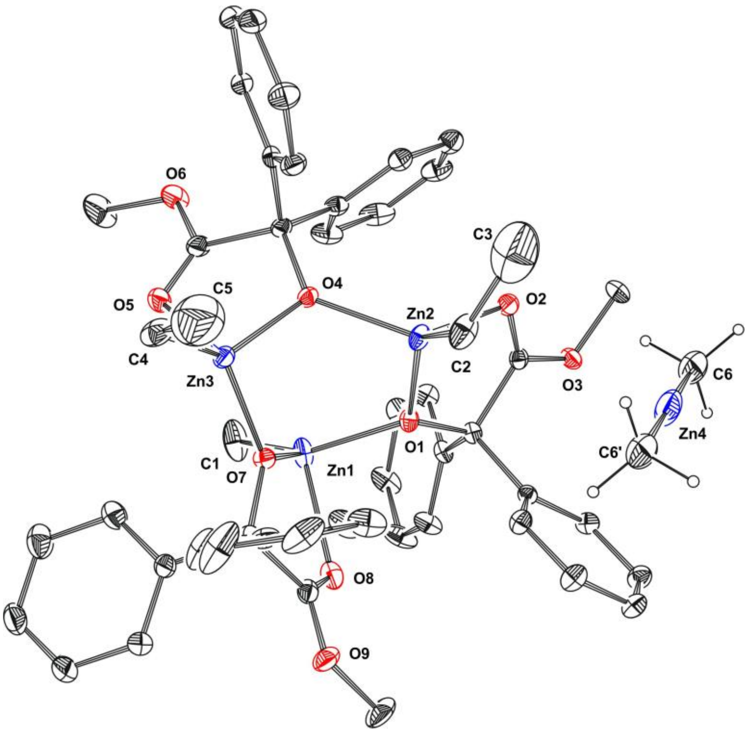

Fig. S24. Molecular structure of $ZnMe_2⊂[3]_2$. Molecular structure of $ZnMe_2⊂[\mathbf{3}]_2$ with thermal ellipsoids set at 30% probability. Hydrogen atoms have been omitted for clarity. Operators for generated equivalent atoms: (-x,-y,-z+2).

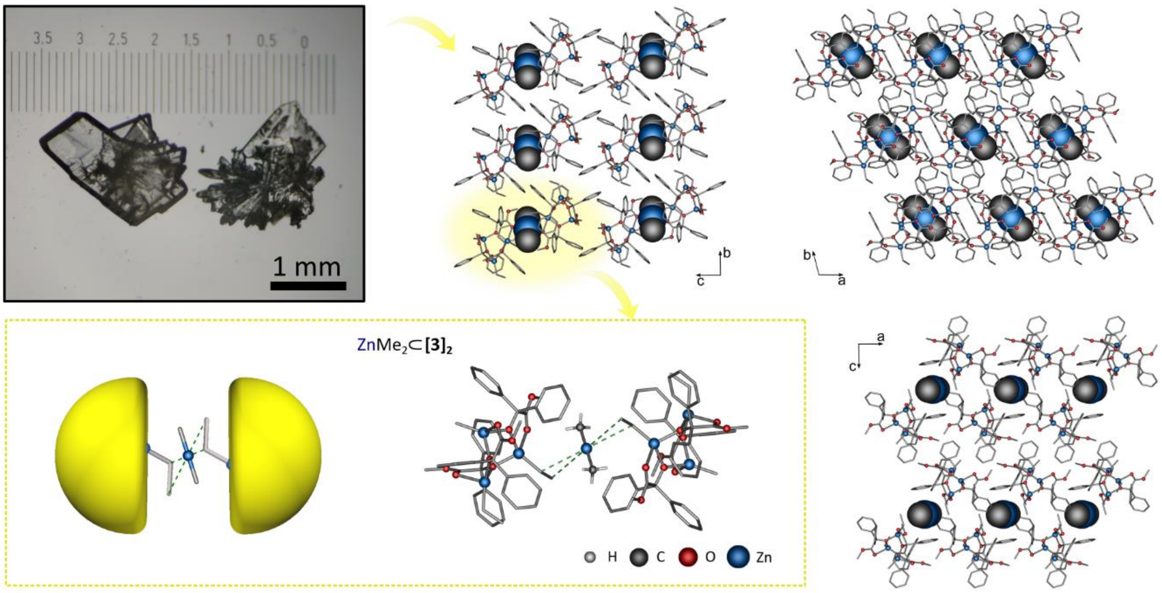

Fig. S25. Crystal structure of $ZnMe_2⊂[3]_2$. Image of $ZnMe_2⊂[\mathbf{3}]_2$ crystals and structure and supramolecular packing of capsule $ZnMe_2⊂[\mathbf{3}]_2$.

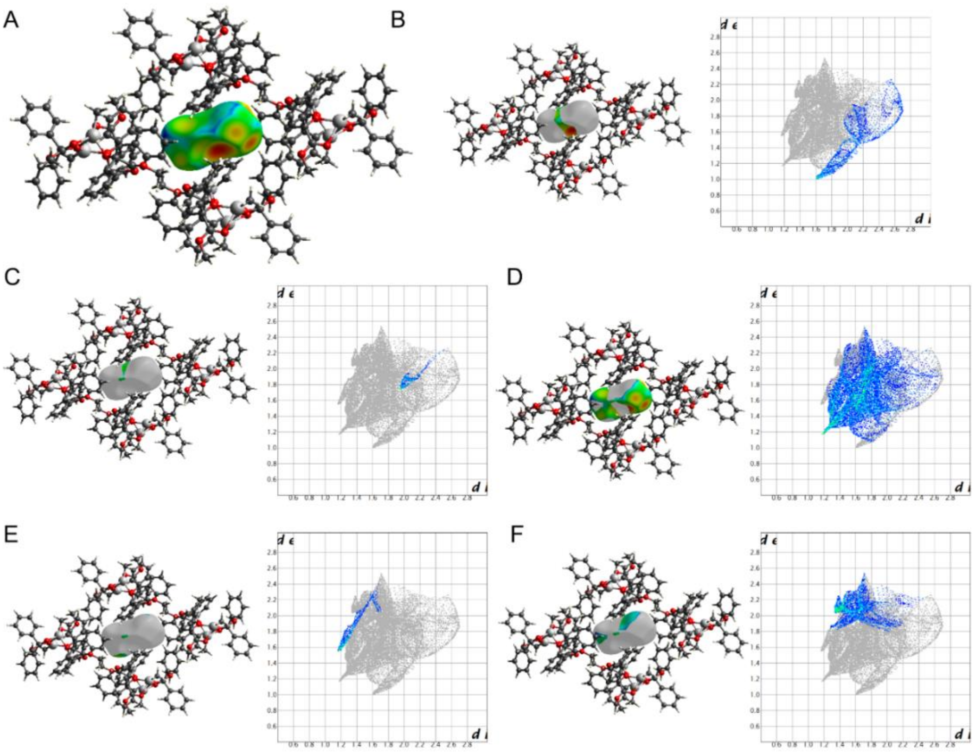

Fig. S26. Hirshfeld surface analysis of $ZnMe_2\subset[\mathbf{1}_3]_2$. Hirshfeld surface for dimethylzinc molecule in $ZnMe_2\subset[\mathbf{1}_3]_2$ (A); Fragments of HS and fingerprints plots for Zn-H (11.6% of the surface) (B), Zn-C (2.6% of the surface) (C), H-H (63.8% of the surface) (D), H-O (4.5% of the surface) (E), and H-C (17.5% of the surface) (F) contacts, respectively.

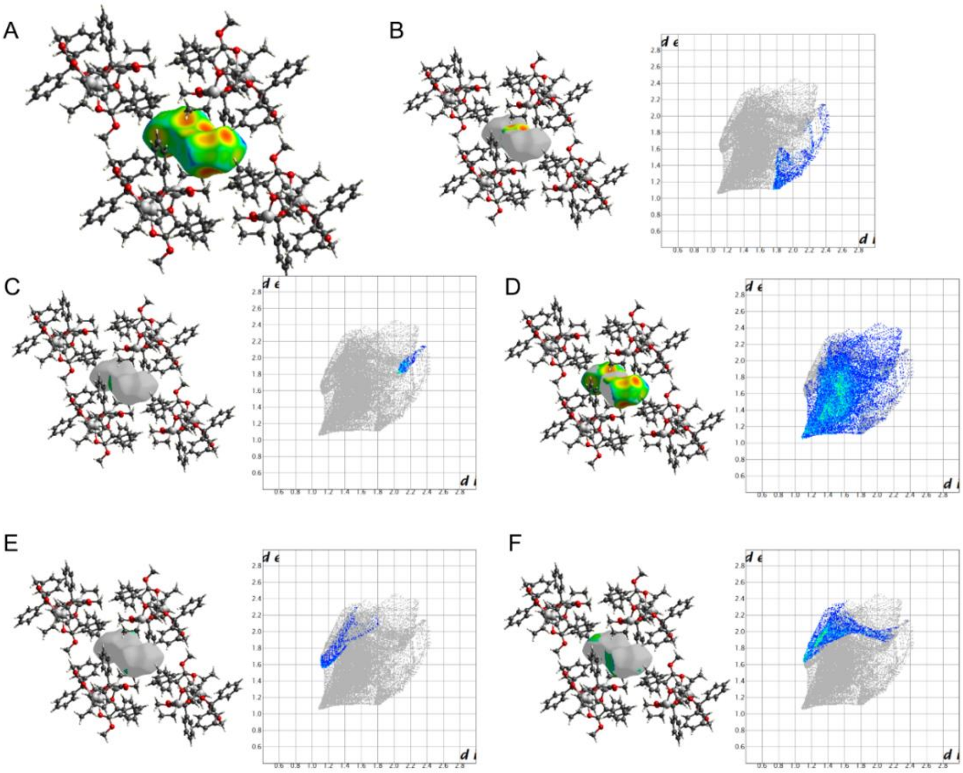

Fig. S27. Hirshfeld surface analysis of $ZnEt_2\subset[\mathbf{2}_3]_2$. Hirshfeld surface for diethylzinc molecule in $Et_2Zn\subset[2_3]_2$ (A); Fragments of HS and fingerprints plots for Zn-H (6.0% of the surface) (B), Zn-C (2.5% of the surface) (C), H-H (72.4% of the surface) (D), H-O (2.7% of the surface) (E), and H-C (16.4% of the surface) (F) contacts, respectively.

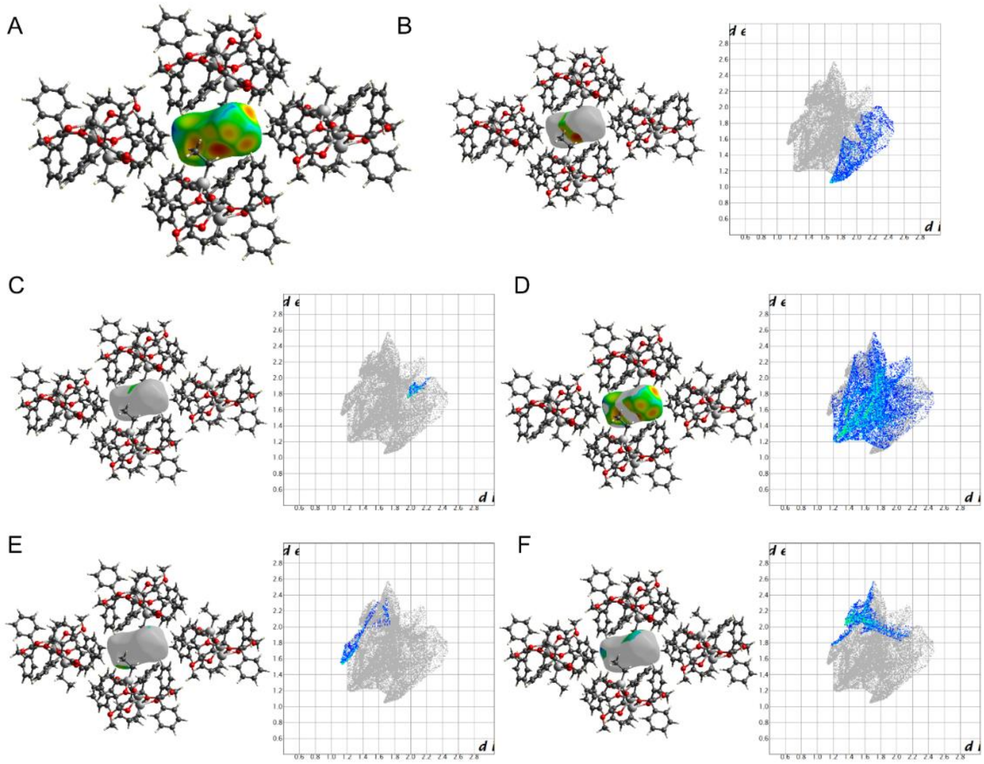

Fig. S28. Hirshfeld surface analysis of $ZnMe_2$⊂[3]$_2$. Hirshfeld surface for dimethylzinc molecule in $ZnMe_2$⊂[3]$_2$ (A); Fragments of HS and fingerprints plots for Zn-H (11.9% of the surface) (B), Zn-C (2.7% of the surface) (C), H-H (65.8% of the surface) (D), H-O (4.3% of the surface) (E), and H-C (15.3% of the surface) (F) contacts, respectively.

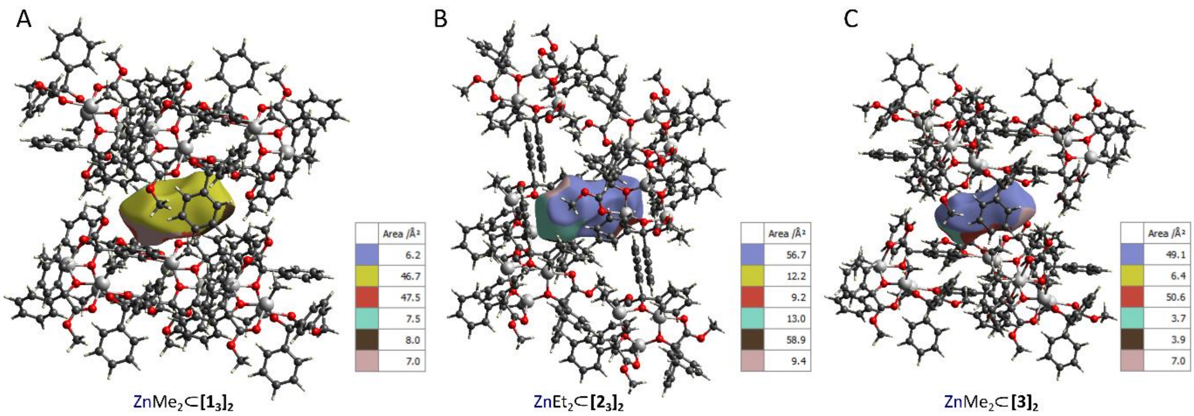

Fig. S29. Hirshfeld surface analysis of guest molecules surroundings. Hirshfeld surface of guest molecules in $ZnMe_2$⊂[**1**$_3$]$_2$ (A), $ZnEt_2$⊂[2$_3$]$_2$ (B), and $ZnMe_2$⊂[3]$_2$ (C) mapped with the fragment patch of neighbor molecules.

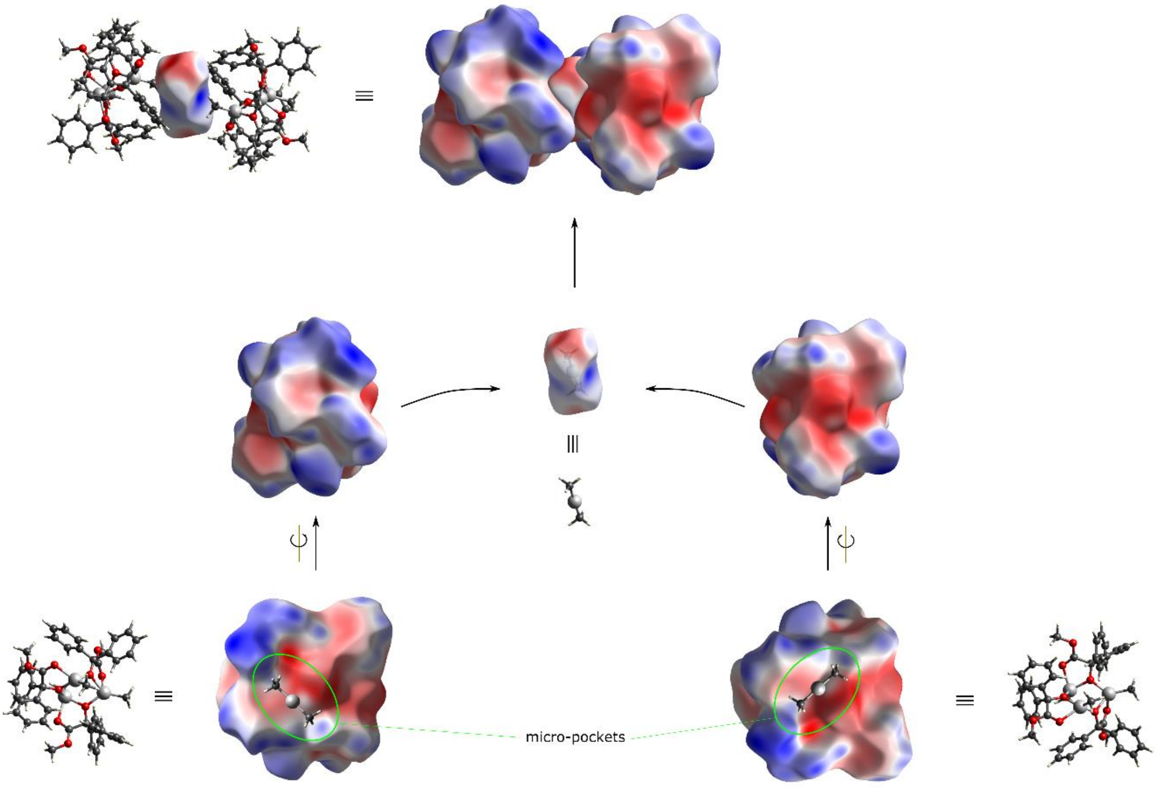

Fig. S30. Electrostatic complementarity during the self-assembly of $ZnMe_2\subset[\mathbf{1_3}]_2$ capsules. Schematic representation of the electrostatic complementarity in $ZnMe_2\subset[\mathbf{1_3}]_2$ host-guest system using Hirshfeld surfaces mapped with electrostatic potential [from -0.05 au (red) to +0.05 au (blue); the individual components were moved apart to show touching of the surfaces].

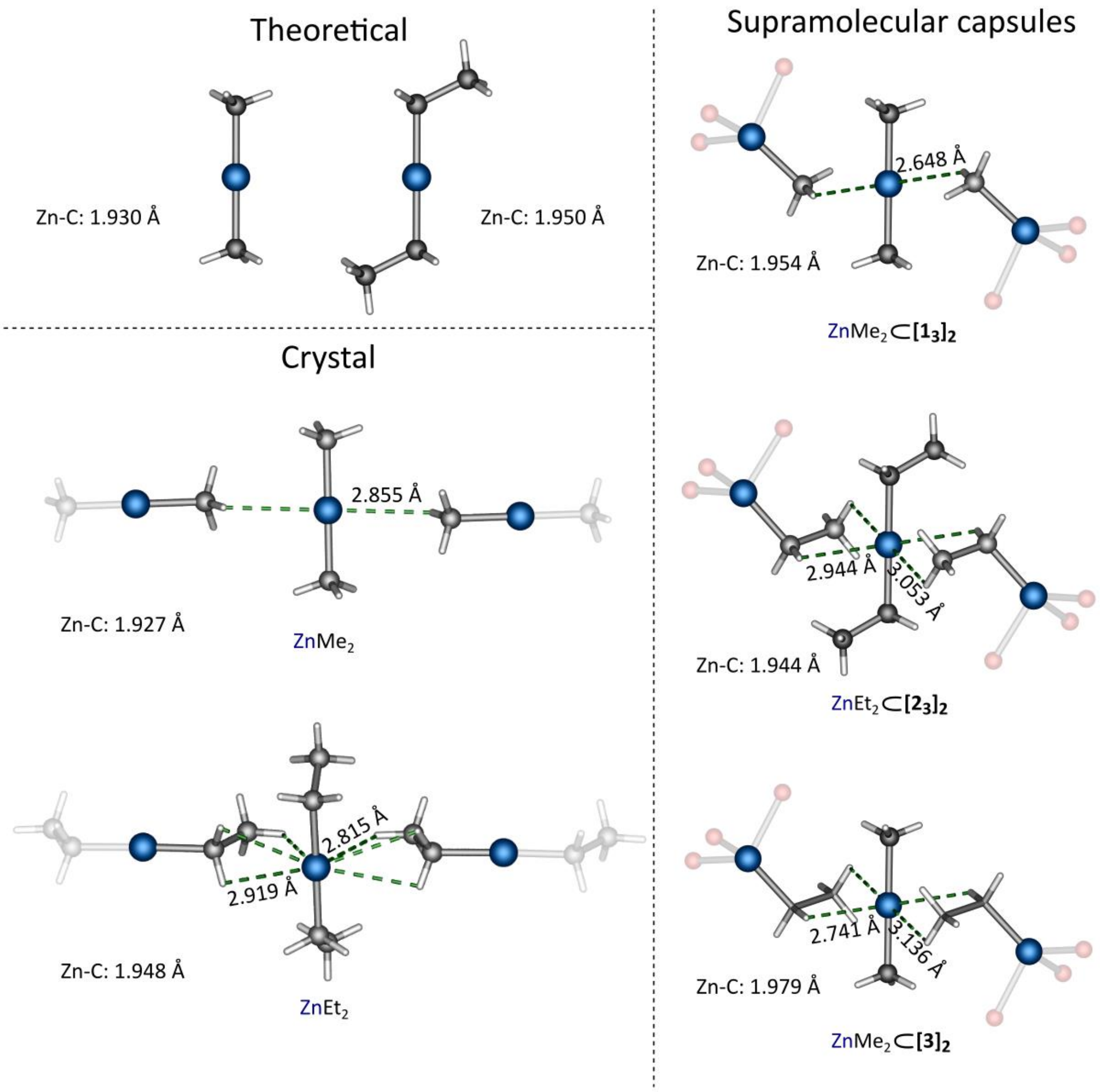

Fig. S31. Molecular structures of ZnR_2 in various environments. Comparison of the molecular structures of $ZnMe_2$ and $ZnEt_2$ from theoretical calculations, crystal structures of neat dialkylzincs, and supramolecular capsules $ZnMe_2$⊂[**1**$_3$]$_2$, $ZnEt_2$⊂[**2**$_3$]$_2$ and $ZnMe_2$⊂[**3**].

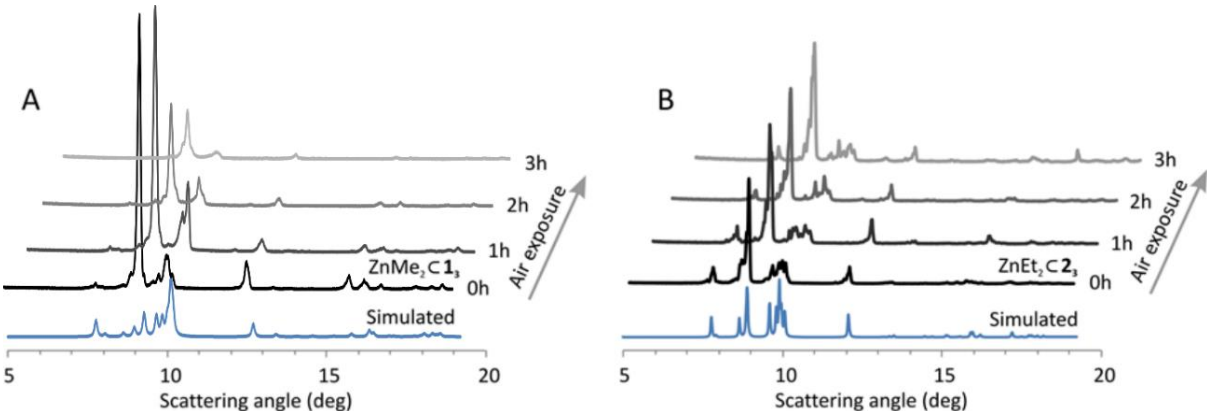

Fig. S32. Changes in the crystal structure of capsules during exposure to air. *In-situ* PXRD patterns for $ZnMe_2\subset[\mathbf{1}_3]_2$ (A) and $ZnEt_2\subset[\mathbf{2}_3]_2$ (B) during exposure to air.

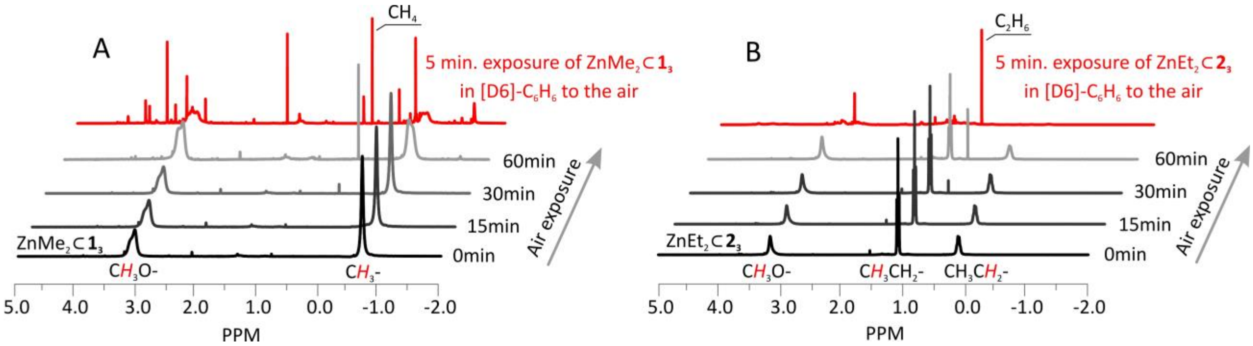

Fig. S33. Spectroscopic studies of the stability of capsules in air. ^{1}H NMR spectra (in C_6D_6) of $ZnMe_2\subset[\mathbf{1}_3]_2$ (A) and $ZnEt_2\subset[\mathbf{2}_3]_2$ (B) after exposition to air in solid state and in C_6D_6 solution.

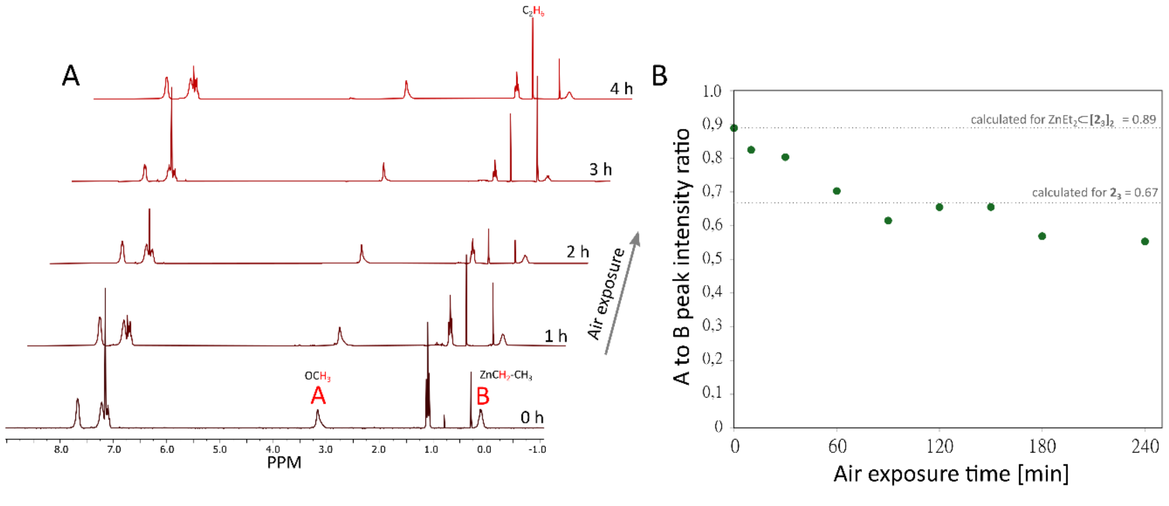

Fig. S34. Zn-Et group decomposition in time during the exposure of $ZnEt_2\subset[\mathbf{2}_3]_2$ to air. ^{1}H NMR spectra (in C_6D_6) collected after prolongated exposition to air of solid-state sample of $ZnEt_2\subset[\mathbf{2}_3]_2$ (A) and the change in the intensity ratio between OMe and Zn-CH_2 resonances over the exposure time (B).

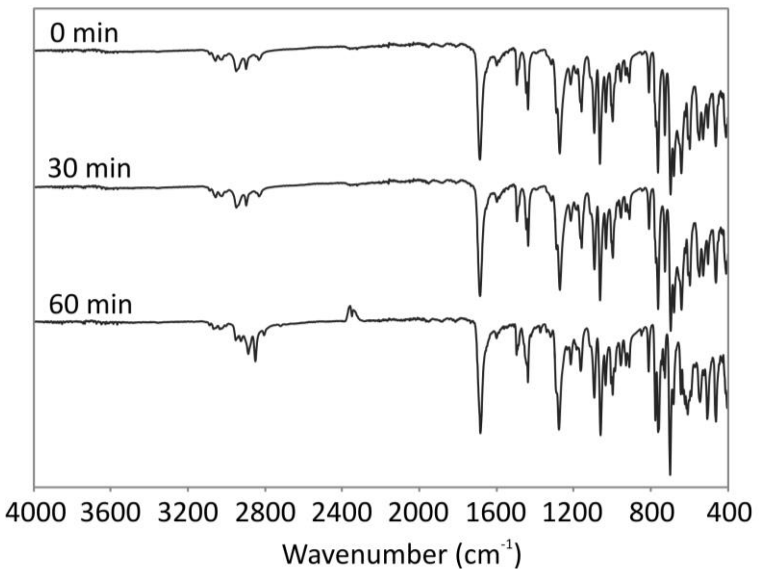

Fig. S35. Changes in IR spectra of $ZnMe_2$⊂[1_3]$_2$ during exposure to air. *In-situ* FTIR spectra for $ZnMe_2$⊂[**1_3**]$_2$ during exposure to air.

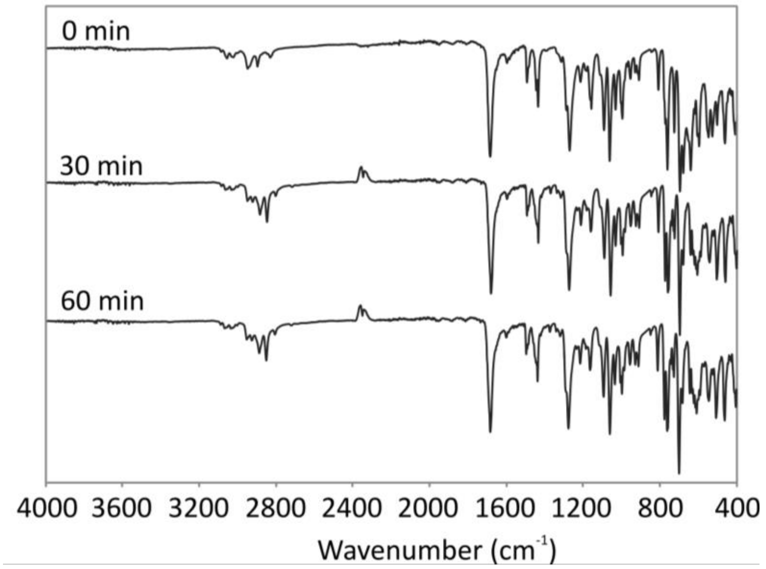

Fig. S36. Changes in IR spectra of $ZnEt_2$⊂[2_3]$_2$ during exposure to air. *In-situ* FTIR spectra for $ZnEt_2$⊂[**2_3**]$_2$ during exposure to air.

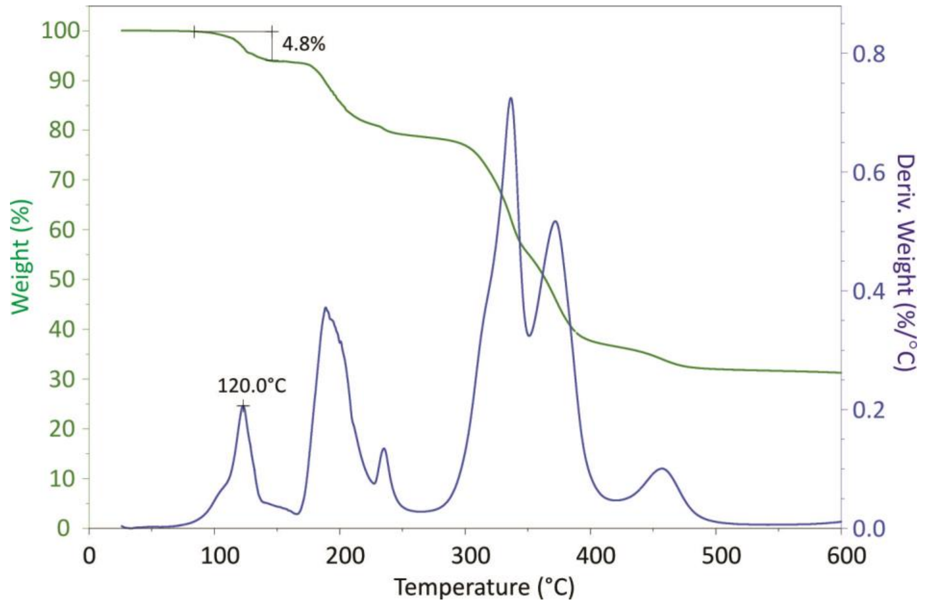

Fig. S37. Thermal stability of ZnMe$_2$⊂[1$_3$]$_2$. TGA profile of sample ZnMe$_2$⊂[**1**$_3$]$_2$.

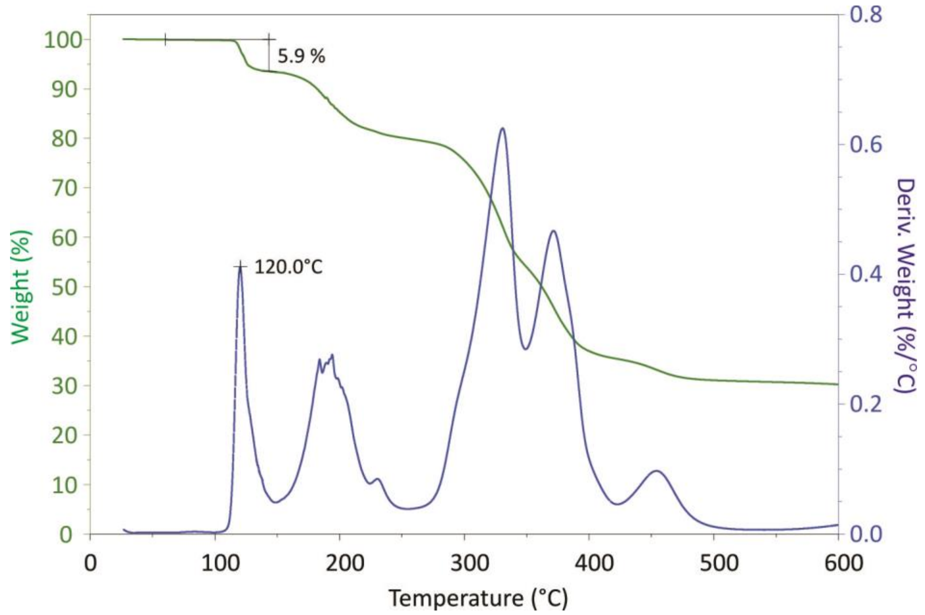

Fig. S38. Thermal stability of ZnEt$_2$⊂[2$_3$]$_2$. TGA profile of sample ZnEt$_2$⊂[**2**$_3$]$_2$.

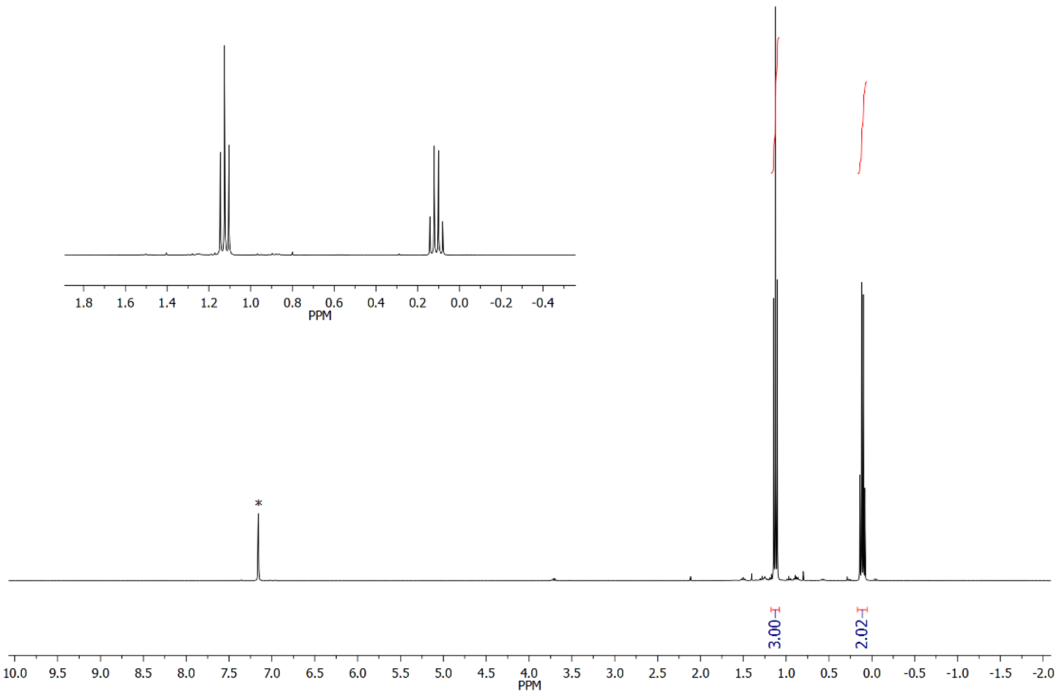

Fig. S39. Purity of $ZnEt_2$ released from $ZnEt_2$⊂[$\mathbf{2}_3$]. ^{1}H NMR spectrum in C_6D_6 of $ZnEt_2$ released from $ZnEt_2$⊂[$\mathbf{2}_3$]. The asterisk denotes the signal from C_6D_6.

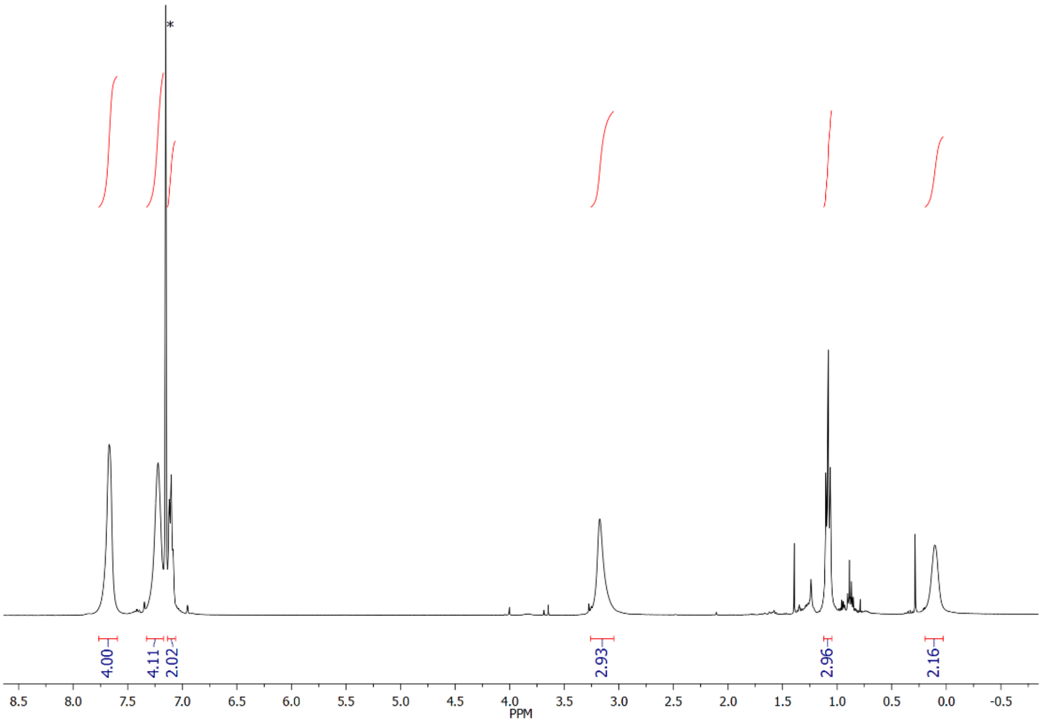

Fig. S40. Spectra of capsule components after guest removal from $ZnEt_2$⊂[$\mathbf{2}_3$]. ^{1}H NMR spectrum in C_6D_6 of $\mathbf{2}_3$ after release $ZnEt_2$ from $ZnEt_2$⊂[$\mathbf{2}_3$] capsules. The asterisk denotes the signal from C_6D_6.

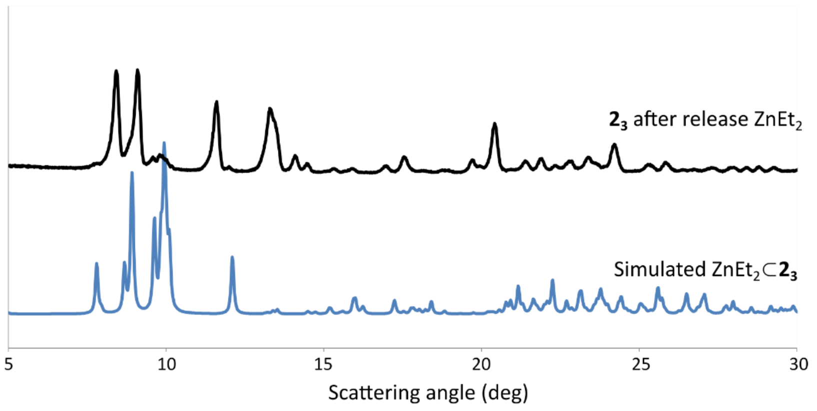

Fig. S41. Powder pattern of capsule after guest removal from $ZnEt_2\subset[2_3]$. PXRD pattern of $\mathbf{2_3}$ after release $ZnEt_2$ from $ZnEt_2\subset[\mathbf{2_3}]$ capsules.

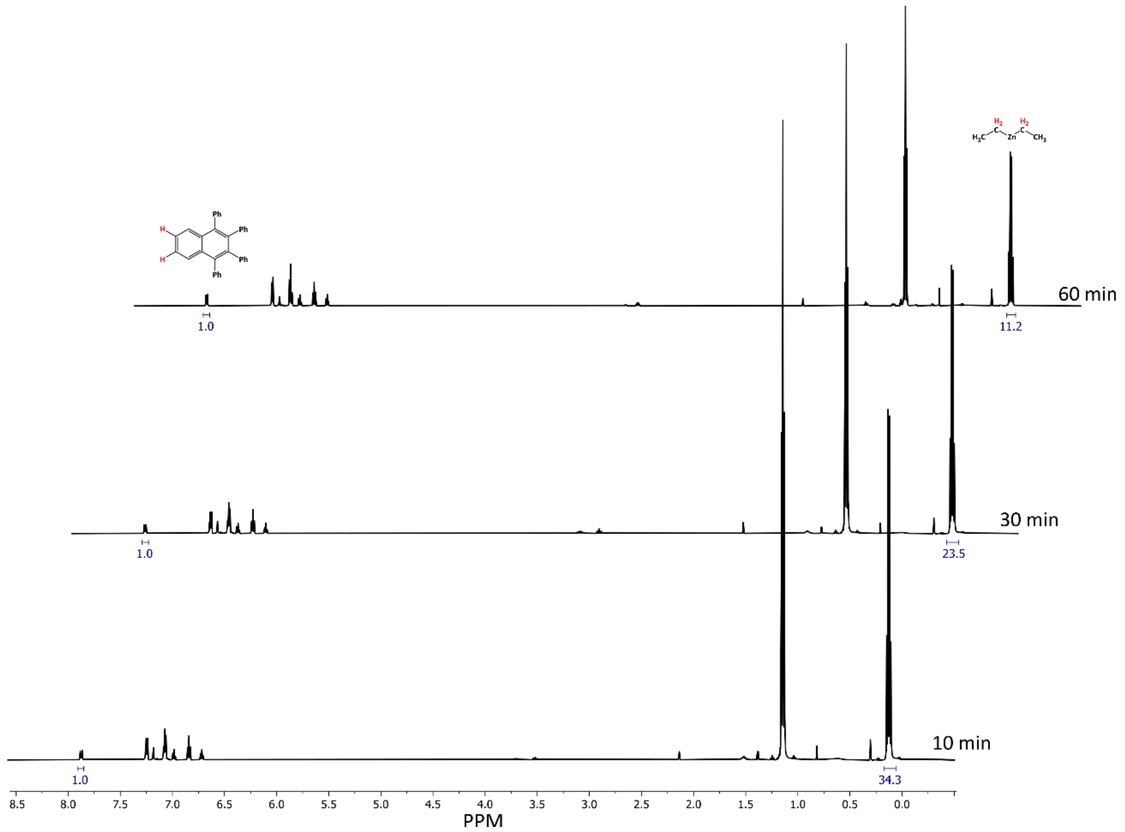

Fig. S42. $ZnEt_2$ guest recovery after exposure of $ZnEt_2\subset[2_3]$ to air. ^{1}H NMR spectra in C_6D_6 of released $ZnEt_2$ from $ZnEt_2\subset[\mathbf{2_3}]$ capsules exposed to air for 10, 30, and 60 min. TPhN was used as an internal standard for quantitative analysis.

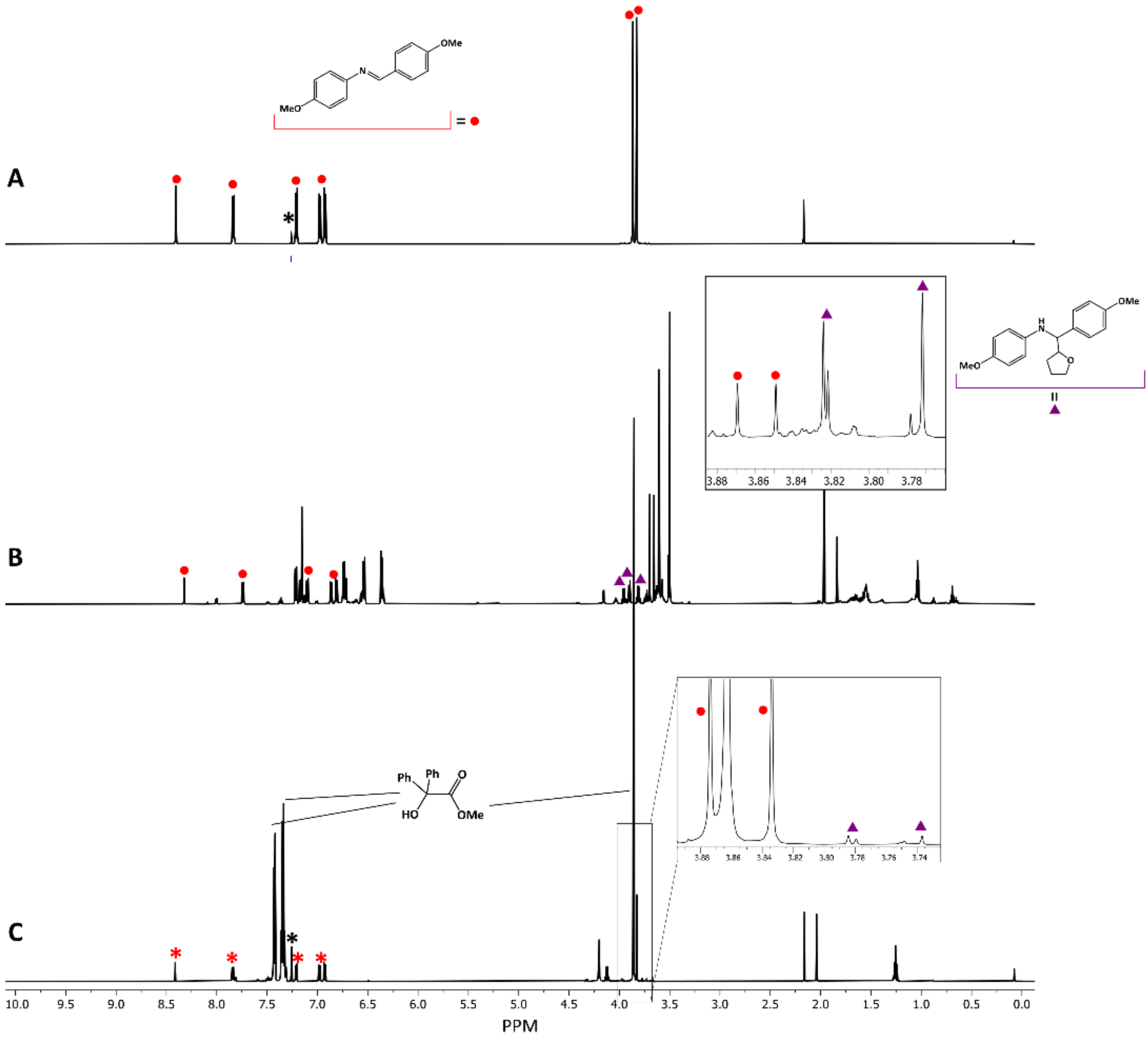

Fig. S43. Application of the $ZnEt_2$-loaded capsules as initiator for the radical addition of THF to imines. ^1H NMR spectra in $CDCl_3$ of the initial 4-methoxy-N-(4-methoxybenzylidene)aniline (A) and product of its THF addition initiated by $[PhCOOZnEt]_n$ (B) and $ZnEt_2 \subset [\mathbf{2}_3]$ (C). The black asterisk denotes the signal from $CDCl_3$. Selected signals from initial imine and products of THF addition are marked by red spheres and purple triangles, respectively.

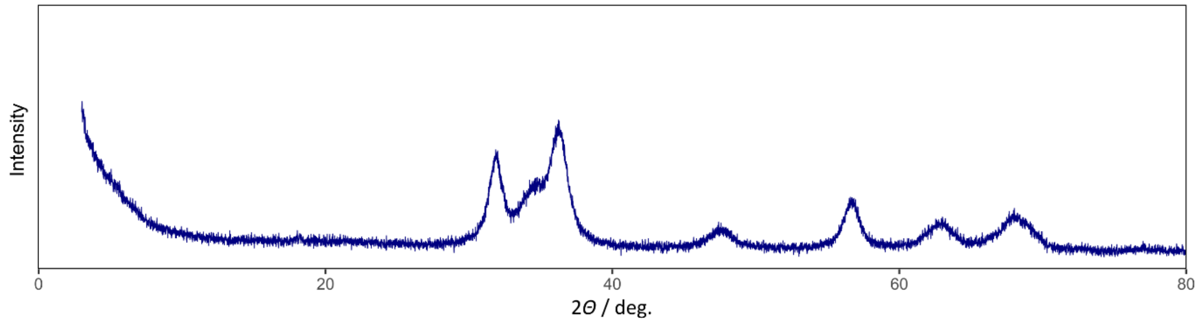

Fig. S44. Crystallinity of the $ZnEt_2 \subset [2_3]_2$-derived ZnO NCs. Powder pattern of ZnO-*Bnz* NCs.

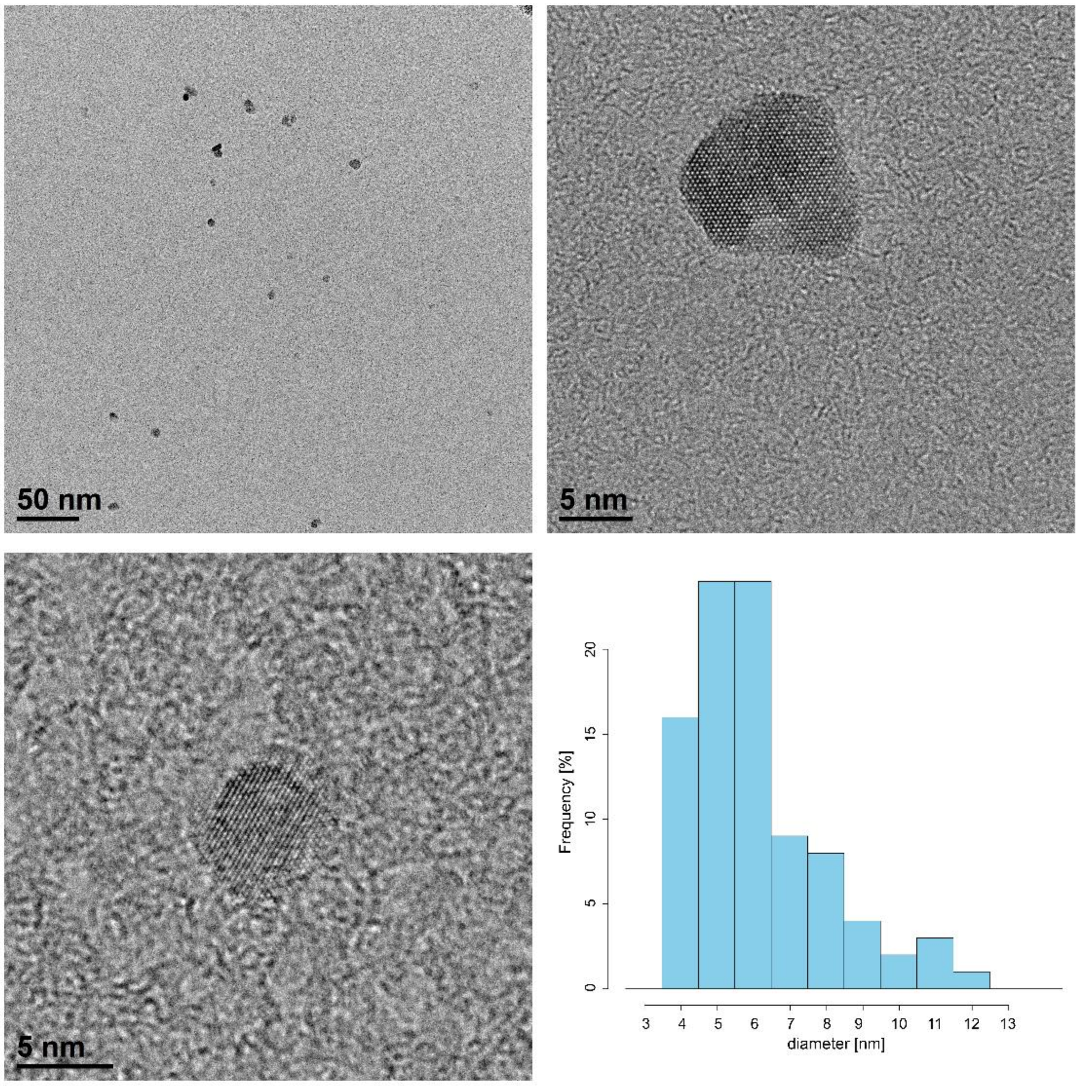

Fig. S45. TEM images of the $ZnEt_2 \subset [2_3]_2$-derived ZnO NCs. Representative TEM images of ZnO-*Bnz* and calculated NC size distribution.

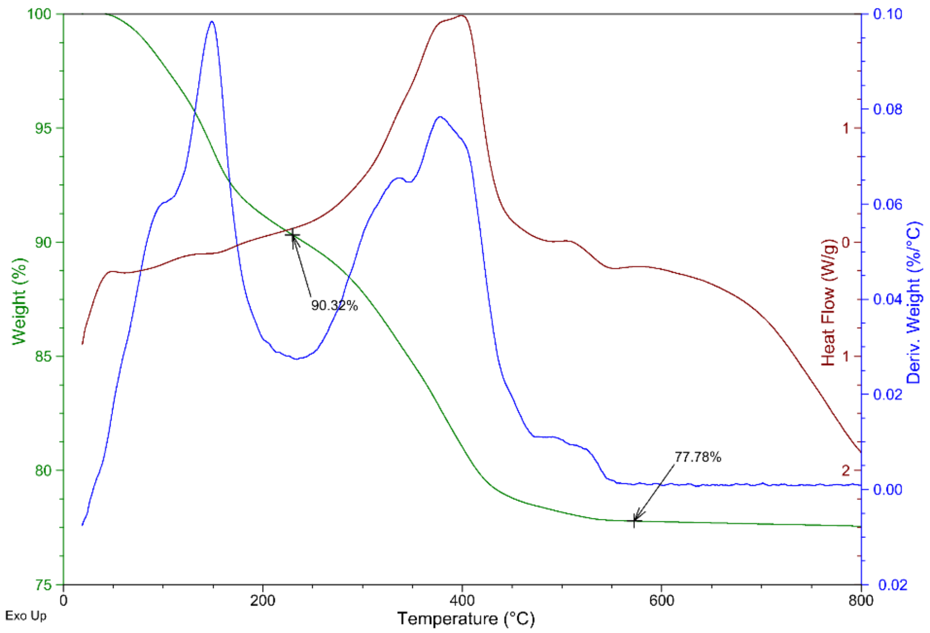

Fig. S46. Thermogravimetric analysis of the $ZnEt_2 \subset [2_3]_2$-derived ZnO NCs. TGA-DSC plot of ZnO-*Bnz* NCs under the air flow.

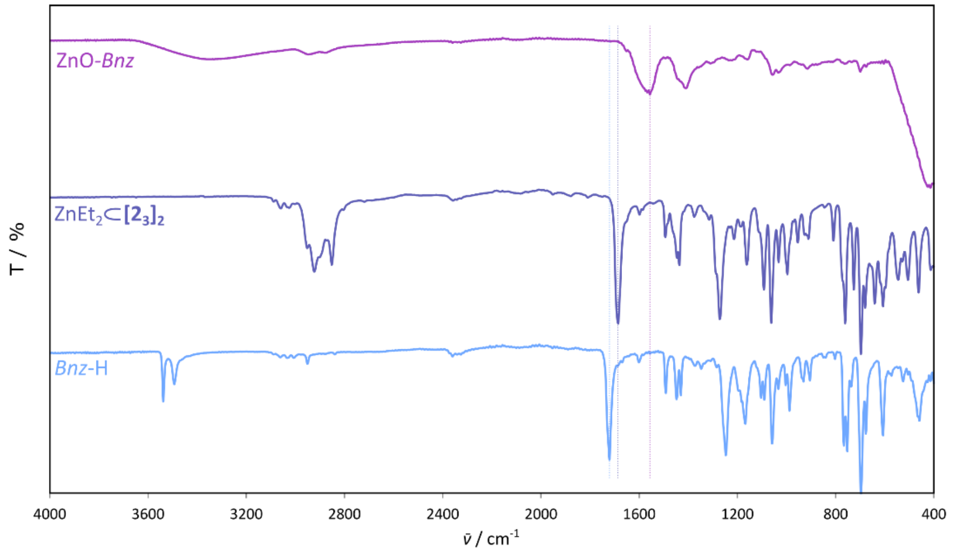

Fig. S47. IR spectroscopic analysis of the $ZnEt_2 \subset [2_3]_2$-derived ZnO NCs. FTIR spectra of ZnO-*Bnz*, $ZnEt_2 \subset [2_3]_2$, and *Bnz*-H.

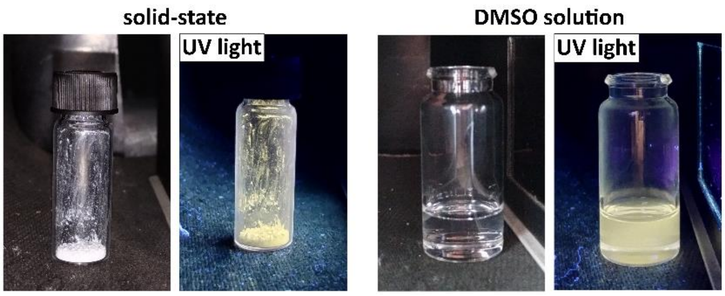

Fig. S48. Images of the $ZnEt_2 \subset [2_3]_2$-derived ZnO NCs. Images of ZnO-*Bnz* NCs in the solid state and a DMSO solution under daylight and UV irradiation.

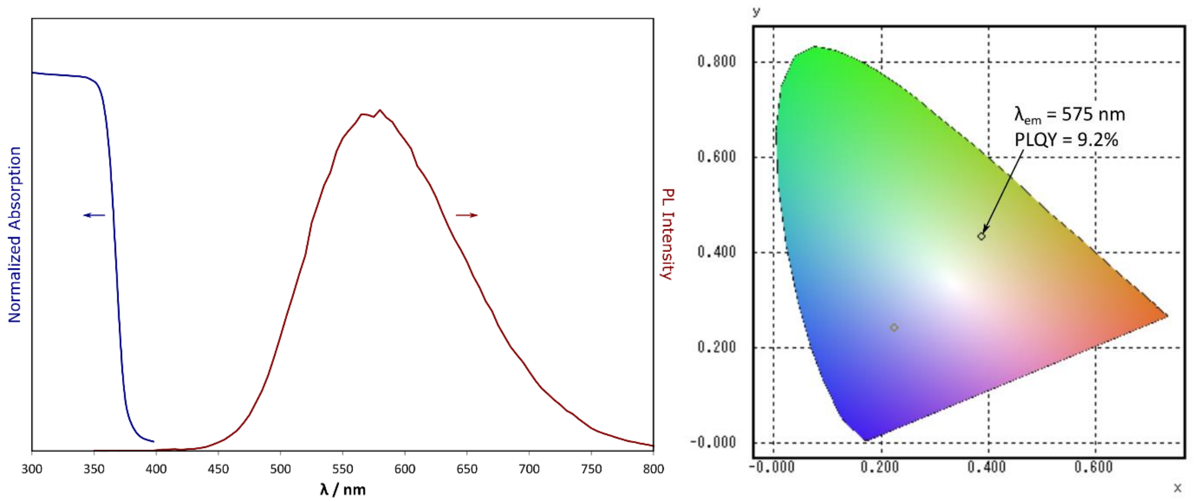

Fig. S49. Optical properties of the $ZnEt_2\subset[2_3]_2$-derived ZnO NCs. UV/Vis and PL spectra of ZnO-*Bnz* NCs and chromaticity diagram for samples excited at λ_{ex} = 310 nm.

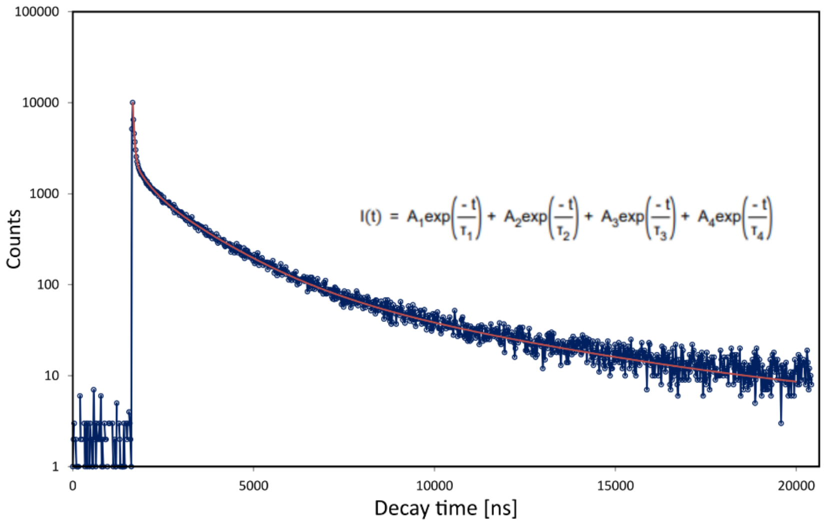

Fig. S50. PL charge recombination lifetime in the $ZnEt_2\subset[2_3]_2$-derived ZnO NCs. Photoluminescence decays taken for ZnO-*Bnz* NCs in the solid state.

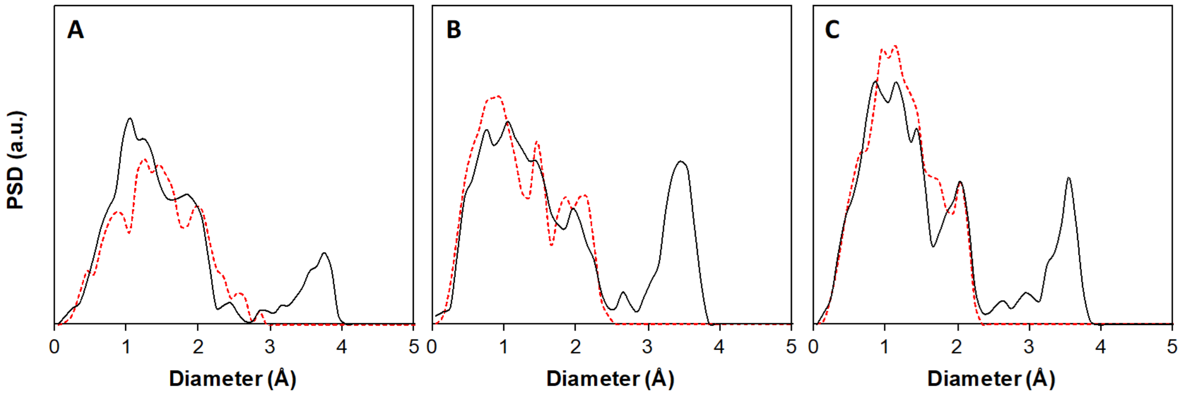

Fig. S51. Analysis of pore size distribution in capsules. Pore size distribution of containers $[\mathbf{1}_3]$ (A), $[\mathbf{2}_3]_2$ (B), and $[\mathbf{3}]_2$ (C), empty (black continuous line) and including the guest molecules (red dotted line).

Supplementary tables

Table S1. Calculated molecular weights of [MeZn(*Bnz*)]$_x$ aggregates. Calculated molecular weights (*MW*), van-der Waals densities (MD_W), and corrected molecular weights (MW_{cor}) of respective [MeZn(*Bnz*)]$_x$ aggregates.

Compound	*MW* [g·mol^{-1}]	MD_W [g·mol^{-1}·m^{-3}]	χ_{cor}	MW_{cor} [g·mol^{-1}]
[MeZn(*Bnz*)]$_2$	643	$6.43 \cdot 10^{29}$	1.39	462
[MeZn(*Bnz*)]$_3$	965	$6.43 \cdot 10^{29}$	1.39	693

Table S2. Estimated molecular weights of [MeZn(*Bnz*)]$_x$ aggregates at room temperature. Determined diffusion coefficients *D* and estimated molecular weights *MW* based on DOSY ^{1}H NMR spectrum of **1$_3$** in d$_8$-toluene at 25°C.

Internal reference (TPhN)		Sample		
[ppm]	*D* [m^2·s^{-1}]	*δ* [ppm]	*D* [m^2·s^{-1}]	*MW* [g·mol^{-1}]
7.77-7.85	$6.64 \cdot 10^{-10}$	Compound A		
		7.51-7.76	$5.56 \cdot 10^{-10}$	687
		3.02-3.32	$5.64 \cdot 10^{-10}$	672
		-0.99-(-0.67)	$5.85 \cdot 10^{-10}$	632
		Average Estimated *MW*		664
		Toluene		
		2.07-2.11	$2.01 \cdot 10^{-9}$	83

Table S3. Estimated molecular weights of [MeZn(*Bnz*)]$_x$ aggregates at low temperature. Determined diffusion coefficients *D* and estimated molecular weights *MW* based on DOSY ^{1}H NMR spectrum of **1$_3$** in [D$_8$]-toluene at -20°C.

Internal reference (TPhN)		Sample		
[ppm]	*D* [m^2·s^{-1}]	[ppm]	*D* [m^2·s^{-1}]	*MW* [g·mol^{-1}]
7.82-7.90	$2.87 \cdot 10^{-10}$	Compound A		
		7.67-7.79	$2.42 \cdot 10^{-10}$	666
		2.96-3.07	$2.29 \cdot 10^{-10}$	726
		-0.80-(-0.59)	$2.33 \cdot 10^{-10}$	708
		Average Estimated *MW*		700
		Compound B		
		7.57-7.64	$2.56 \cdot 10^{-10}$	604
		3.11-3.17	$2.63 \cdot 10^{-10}$	581
		-0.86-(-0.81)	$2.59 \cdot 10^{-10}$	593
		Average Estimated *MW*		592
		Toluene		
		2.05-2.15	$8.40 \cdot 10^{-10}$	86

Table S4. Calculated molecular weights of [EtZn(*Bnz*)]$_x$ aggregates. Calculated molecular weights (*MW*), van-der Waals densities (MD_W), and corrected molecular weights (MW_{cor}) of respective [EtZn(*Bnz*)]$_x$ aggregates.

Compound	*MW* [g·mol^{-1}]	MD_W [g·mol^{-1}·m^{-3}]	χ_{cor}	MW_{cor} [g·mol^{-1}]
[EtZn(*Bnz*)]$_2$	671	$6.27\cdot10^{29}$	1.37	492
[EtZn(*Bnz*)]$_3$	1007	$6.27\cdot10^{29}$	1.37	737

Table S5. Estimated molecular weights of [EtZn(*Bnz*)]$_x$ aggregates at room temperature. Determined diffusion coefficients *D* and estimated molecular weights *MW* based on DOSY ^{1}H NMR spectrum of **2$_3$** in d$_8$-toluene at 25°C.

Internal reference (TPhN)		Sample		
[ppm]	*D* [m^2·s^{-1}]	[ppm]	*D* [m^2·s^{-1}]	*MW* [g·mol^{-1}]
7.75-7.86	$6.72\cdot10^{-10}$	Compound A		
		7.53-7.69	$5.62\cdot10^{-10}$	671
		3.06-3.38	$5.58\cdot10^{-10}$	679
		0.91-1.08	$5.62\cdot10^{-10}$	670
		-0.11-0.19	$5.61\cdot10^{-10}$	673
		Average Estimated *MW*		673
		Toluene		
		2.07-2.11	$1.98\cdot10^{-9}$	85

Table S6. Estimated molecular weights of [EtZn(*Bnz*)]$_x$ aggregates at low temperature. Determined diffusion coefficients *D* and estimated molecular weights *MW* based on DOSY ^{1}H NMR spectrum of **2$_3$** in d$_8$-toluene at -20°C.

Internal reference (TPhN)		Sample		
[ppm]	*D* [m^2·s^{-1}]	[ppm]	*D* [m^2·s^{-1}]	*MW* [g·mol^{-1}]
7.81-7.91	$2.80\cdot10^{-10}$	Compound A		
		7.79-7.81	$2.11\cdot10^{-10}$	799
		3.02-3.12	$2.09\cdot10^{-10}$	809
		1.09-1.19	$2.09\cdot10^{-10}$	808
		0.07-0.28	*	*
		Average Estimated *MW*		805
		Compound B		
		7.60-7.66	$2.81\cdot10^{-10}$	499
		3.16-3.22	$2.82\cdot10^{-10}$	495
		1.01-1.09	$2.76\cdot10^{-10}$	513
		-0.04-0.07	$2.77\cdot10^{-10}$	510
		Average Estimated *MW*		505
		Toluene		
		2.06-2.11	$8.63\cdot10^{-10}$	80

Table S7. Crystal data for 1_3·3PhMe. Crystallographic data and structure refinement parameters for **1_3·3PhMe**.

Empirical formula	$C_{48}H_{48}O_9Zn_3$·3PhMe	
Formula weight	1241.37	
Temperature	100(2) K	
Wavelength	0.71073 Å	
Crystal system	Triclinic	
Space group	$P\bar{1}$	
Unit cell dimensions	a = 14.3900(6) Å	α = 98.248(2)°.
	b = 17.0540(7) Å	β = 101.505(2)°.
	c = 27.5390(11) Å	γ = 92.405(2)°.
Volume	6536.6(5) Å^3	
Z	4	
Density (calculated)	1.261 Mg/m^3	
Absorption coefficient	1.145 mm^{-1}	
F(000)	2592	
Crystal size	0.18 x 0.14 x 0.08 mm^3	
θ range for data collection	2.016 to 24.116°.	
Index ranges	$-16 \le h \le 16$, $-19 \le k \le 19$, $-31 \le l \le 31$	
Reflections collected	20365	
Independent reflections	20365 [R_{int} = 0.0398]	
Completeness to θ = 20.97°	97.8 %	
Refinement method	Full-matrix least-squares on F^2	
Data / restraints / parameters	20365 / 0 / 1477	
Goodness-of-fit on F^2	0.957	
Final R indices [$I > 2\sigma(I)$]	R = 0.0449, wR = 0.1110	
R indices (all data)	R = 0.0735, wR = 0.1203	
Largest diff. peak and hole	0.91 and -0.52 e.Å^{-3}	

Table S8. Crystal data for $\mathbf{2_3}$·0.5PhMe. Crystallographic data and structure refinement parameters for **$\mathbf{2_3}$·0.5PhMe**.

Empirical formula	$2(C_{51}H_{54}O_9Zn_3)$·PhMe	
Formula weight	2106.23	
Temperature	100(2) K	
Wavelength	0.71073 Å	
Crystal system	Triclinic	
Space group	$P\bar{1}$	
Unit cell dimensions	a = 12.7120(2) Å	α = 107.5780(10)°.
	b = 20.1160(4) Å	β = 95.4950(10)°.
	c = 20.8220(5) Å	γ = 95.1030(10)°.
Volume	5013.42(18) Å^3	
Z	2	
Density (calculated)	1.395 Mg/m^3	
Absorption coefficient	1.479 mm^{-1}	
F(000)	2188	
Crystal size	0.17 x 0.13 x 0.06 mm^3	
θ range for data collection	1.070 to 25.681°.	
Index ranges	$-15 \le h \le 15$, $-24 \le k \le 24$, $-25 \le l \le 25$	
Reflections collected	34749	
Independent reflections	18815 [R_{int} = 0.0496]	
Completeness to θ = 20.97°	98.7 %	
Refinement method	Full-matrix least-squares on F^2	
Data / restraints / parameters	18815 / 0 / 1211	
Goodness-of-fit on F^2	1.051	
Final R indices [$I > 2\sigma(I)$]	R_1 = 0.0559, wR_2 = 0.1387	
R indices (all data)	R_1 = 0.0738, wR_2 = 0.1482	
Largest diff. peak and hole	1.03 and -0.81 e.Å^{-3}	

Table S9. Crystal data for $ZnMe_2⊂[\mathbf{1}_3]_2$. Crystallographic data and structure refinement parameters for $ZnMe_2⊂[\mathbf{1}_3]_2$.

Empirical formula	$2(C_{48}H_{48}O_9Zn_3)·ZnMe_2$	
Formula weight	2025.38	
Temperature	100(2) K	
Wavelength	0.71073 Å	
Crystal system	Triclinic	
Space group	$P\bar{1}$	
Unit cell dimensions	a = 11.9373(3) Å	α = 93.3010(10)°.
	b = 12.3745(2) Å	β = 91.9740(10)°.
	c = 17.4381(4) Å	γ = 113.9870(10)°.
Volume	2344.82(9) $Å^3$	
Z	1	
Density (calculated)	1.434 Mg/m^3	
Absorption coefficient	1.825 mm^{-1}	
F(000)	1044	
Crystal size	0.16 x 0.13 x 0.07 mm^3	
θ range for data collection	1.807 to 25.680°.	
Index ranges	$-14 \leq h \leq 14$, $-14 \leq k \leq 15$, $-21 \leq l \leq 21$	
Reflections collected	16986	
Independent reflections	8876 [R_{int} = 0.0362]	
Completeness to θ = 20.97°	99.7 %	
Refinement method	Full-matrix least-squares on F^2	
Data / restraints / parameters	8876 / 0 / 563	
Goodness-of-fit on F^2	1.034	
Final R indices [$I > 2\sigma(I)$]	R = 0.0441, wR = 0.0928	
R indices (all data)	R = 0.0600, wR = 0.0986	
Largest diff. peak and hole	1.06 and -0.66 e.$Å^{-3}$	

Table S10. Crystal data for $ZnEt_2$⊂[$\mathbf{2}_3$]$_2$. Crystallographic data and structure refinement parameters for $ZnEt_2$⊂[$\mathbf{2}_3$]$_2$.

Empirical formula	$2(C_{51}H_{54}O_9Zn_3)\cdot ZnEt_2$	
Formula weight	2137.59	
Temperature	100(2) K	
Wavelength	0.71073 Å	
Crystal system	Triclinic	
Space group	$P\bar{1}$	
Unit cell dimensions	a = 12.12200(10) Å	α = 92.8320(10)°.
	b = 12.3240(2) Å	β = 94.5480(10)°.
	c = 17.7940(3) Å	γ = 112.7690(10)°.
Volume	2434.11(6) $Å^3$	
Z	1	
Density (calculated)	1.458 Mg/m^3	
Absorption coefficient	1.762 mm^{-1}	
F(000)	1108	
Crystal size	0.15 x 0.12 x 0.05 mm^3	
θ range for data collection	1.807 to 25.680°.	
Index ranges	$-15 \le h \le 15$, $-16 \le k \le 16$, $-23 \le l \le 23$	
Reflections collected	19779	
Independent reflections	11026 [R_{int} = 0.0293]	
Completeness to θ = 20.97°	99.0 %	
Refinement method	Full-matrix least-squares on F^2	
Data / restraints / parameters	11026 / 0 / 599	
Goodness-of-fit on F^2	1.034	
Final R indices [$I > 2\sigma(I)$]	R = 0.0405, wR = 0.0874	
R indices (all data)	R = 0.0493, wR = 0.0913	
Largest diff. peak and hole	0.88 and -1.58 e.$Å^{-3}$	

Table S11. Crystal data for $ZnMe_2$⊂[3]$_2$. Crystallographic data and structure refinement parameters for $ZnMe_2$⊂**[3]**$_2$.

Empirical formula	$2(C_{50}H_{52}O_9Zn_3)\cdot(ZnEt_2)$	
Formula weight	2081.48	
Temperature	100(2) K	
Wavelength	0.71073 Å	
Crystal system	Triclinic	
Space group	$P\bar{1}$	
Unit cell dimensions	a = 12.0110(2) Å	α = 93.6200(10)°.
	b = 12.4290(2) Å	β = 91.8680(10)°.
	c = 17.4410(3) Å	γ = 114.1780(10)°.
Volume	2365.64(7)Å^3	
Z	1	
Density (calculated)	1.461 Mg/m^3	
Absorption coefficient	1.811 mm^{-1}	
F(000)	1076	
Crystal size	0.16 x 0.11 x 0.06 mm^3	
θ range for data collection	1.802 to 26.372°.	
Index ranges	$-15 \le h \le 15$, $-15 \le k \le 15$, $-21 \le l \le 21$	
Reflections collected	17805	
Independent reflections	9544 [R_{int} = 0.0277]	
Completeness to θ = 20.97°	98.7%	
Refinement method	Full-matrix least-squares on F^2	
Data / restraints / parameters	9544 / 46 / 600	
Goodness-of-fit on F^2	1.044	
Final R indices [$I > 2\sigma(I)$]	R = 0.0499, wR = 0.1203	
R indices (all data)	R = 0.0581, wR = 0.1244	
Largest diff. peak and hole	1.44 and -1.21 e.Å^{-3}	

Table S12. Calculated energy of free and encapsulated $ZnEt_2$ molecules. Valence, non-bond and total energy in kJ/mol for pure $ZnEt_2$ phase, and $ZnEt_2$ encapsulated in **2**$_3$.

		Crystalline $ZnEt_2$ cis	$ZnEt_2$⊂$[2_3]_2$ trans
Valence energy		331.6	329.9
	Bond	221.6	217.6
	Angle	110.0	112.3
	Torsion	0.0	0.0
	Inversion	0.0	0.0
Non-bond energy		122.6	129.8
	van der Waals	1.8	3.5
	Electrostatic	120.8	126.3
Total Energy		454.2	459.7

Table S13. Calculated binding energy of ZnR_2 guests within the capsules. Valence, non-bond, and total energy in kJ/mol for the different species, and binding energies (BE) for $Me_2Zn\subset[\mathbf{1}_3]_2$, $Et_2Zn\subset[\mathbf{2}_3]_2$ and $Me_2Zn\subset[\mathbf{3}]_2$.

	Me_2Zn	$\mathbf{1}_3$	$Me_2Zn\subset\mathbf{1}_3$	Et_2Zn	$\mathbf{2}_3$	$Et_2Zn\subset\mathbf{2}_3$
Valence energy	399.5	7372.7	7772.3	329.9	6744.0	7073.8
Bond	283.9	4803.7	5087.6	217.6	4225.2	4442.7
Angle	115.6	2508.0	2623.6	112.3	2456.6	2568.9
Torsion	0.0	60.6	60.6	0.0	61.8	61.9
Inversion	0.0	0.4	0.4	0.0	0.3	0.3
Non-bond energy	-61.0	-725.4	-884.0	129.8	105.9	121.1
van der Waals	-0.9	120.0	42.1	3.5	11.1	-109.8
Electrostatic	-60.1	-845.4	-926.1	126.3	94.8	230.8
Total Energy	338.6	6647.4	6888.3	459.7	6849.9	7194.9
BE			-97.6			-114.7

	Me_2Zn	**3**	$Me_2Zn\subset\mathbf{3}$
Valence energy	245.2	11845.9	12091.2
Bond	140.3	6639.6	6780.0
Angle	104.9	4802.6	4907.5
Torsion	0.0	403.7	403.7
Inversion	0.0	0.0	0.0
Non-bond energy	-57.6	-130.3	-287.1
van der Waals	-0.8	90.1	6.9
Electrostatic	-56.8	-220.4	-294.0
Total Energy	187.6	11715.6	11804.1
BE			-99.1

REFERENCES AND NOTES

1. A. G. Slater, A. I. Cooper, Function-led design of new porous materials. *Science* **348**, aaa8075 (2015).

2. S. Zarra, D. M. Wood, D. A. Roberts, J. R. Nitschke, Molecular containers in complex chemical systems. *Chem. Soc. Rev.* **44**, 419–432 (2015).

3. F. Dean Toste, Beyond the molecule. *Acc. Chem. Res.* **51**, 2980–2981 (2018).

4. W. Liu, J. F. Stoddart, Emergent behavior in nanoconfined molecular containers. *Chem* **7**, 919–947 (2021).

5. D. S. Kim, J. L. Sessler, Calix[4]pyrroles: Versatile molecular containers with ion transport, recognition, and molecular switching functions. *Chem. Soc. Rev.* **44**, 532–546 (2015).

6. S. J. Barrow, S. Kasera, M. J. Rowland, J. Del Barrio, O. A. Scherman, Cucurbituril-based molecular recognition. *Chem. Rev.* **115**, 12320–12406 (2015).

7. D. Prochowicz, A. Kornowicz, J. Lewiński, Interactions of native cyclodextrins with metal ions and inorganic nanoparticles: Fertile landscape for chemistry and materials science. *Chem. Rev.* **117**, 13461–13501 (2017).

8. T. Hasell, A. I. Cooper, Porous organic cages: Soluble, modular and molecular pores. *Nat. Rev. Mater.* **1**, 16053 (2016).

9. X. Yang, Z. Ullah, J. F. Stoddart, C. T. Yavuz, Porous organic cages. *Chem. Rev.* **123**, 4602–4634 (2023).

10. A. Llamosí, M. P. Szymański, A. Szumna, Molecular vessels from preorganised natural building blocks. *Chem. Soc. Rev.* **53**, 4434–4462 (2024).

11. T. R. Cook, P. J. Stang, Recent developments in the preparation and chemistry of metallacycles and metallacages via coordination. *Chem. Rev.* **115**, 7001–7045 (2015).

12. F. J. Rizzuto, L. K. S. von Krbek, J. R. Nitschke, Strategies for binding multiple guests in metal–organic cages. *Nat. Rev. Chem.* **3**, 204–222 (2019).

13. L. Shao, X. Hu, K. Sikligar, G. A. Baker, J. L. Atwood, Coordination polymers constructed from pyrogallol[4]arene-assembled metal–organic nanocapsules. *Acc. Chem. Res.* **54**, 3191–3203 (2021).

14. C. T. McTernan, J. A. Davies, J. R. Nitschke, Beyond platonic: How to build metal-organic polyhedra capable of binding low-symmetry, information-rich molecular cargoes. *Chem. Rev.* **122**, 10393–10437 (2022).

15. I. Hisaki, C. Xin, K. Takahashi, T. Nakamura, Designing hydrogen-bonded organic frameworks (HOFs) with permanent porosity. *Angew. Chem. Int. Ed. Engl.* **58**, 11160–11170 (2019).

16. R. B. Lin, B. Chen, Hydrogen-bonded organic frameworks: Chemistry and functions. *Chem* **8**, 2114–2135 (2022).

17. Z. Zhang, Y. Ye, S. Xiang, B. Chen, Exploring multifunctional hydrogen-bonded organic framework materials. *Acc. Chem. Res.* **55**, 3752–3766 (2022).

18. H. Furukawa, K. E. Cordova, M. O'Keeffe, O. M. Yaghi, The chemistry and applications of metal-organic frameworks. *Science* **341**, 1230444 (2013).

19. R. B. Lin, Z. Zhang, B. Chen, Achieving high performance metal-organic framework materials through pore engineering. *Acc. Chem. Res.* **54**, 3362–3376 (2021).

20. T. Kaczorowski, I. Justyniak, T. Lipińska, J. Lipkowski, J. Lewiński, Metal complexes of cinchonine as chiral building blocks: A strategy for the construction of nanotubular architectures and helical coordination polymers. *J. Am. Chem. Soc.* **131**, 5393–5395 (2009).

21. G. Couderc, J. Hulliger, Channel forming organic crystals: Guest alignment and properties. *Chem. Soc. Rev.* **39**, 1545–1554 (2010).

22. Y.-M. Legrand, A. van der Lee, M. Barboiu, Single-crystal x-ray structure of 1,3-dimethylcyclobutadiene by confinement in a crystalline matrix. *Science* **329**, 299–302 (2010).

23. A. Schwenger, W. Frey, C. Richert, Reagents with a crystalline coat. *Angew. Chem. Int. Ed. Engl.* **55**, 13706–13709 (2016).

24. L. Liu, J. R. Wang, X. Mei, Enhancing the stability of active pharmaceutical ingredients by the cocrystal strategy. *CrystEngComm* **24**, 2002–2022 (2022).

25. G. Olivo, G. Capocasa, D. Del Giudice, O. Lanzalunga, S. Di Stefano, New horizons for catalysis disclosed by supramolecular chemistry. *Chem. Soc. Rev.* **50**, 7681–7724 (2021).

26. J. Liu, T. A. Goetjen, Q. Wang, J. G. Knapp, M. C. Wasson, Y. Yang, Z. H. Syed, M. Delferro, J. M. Notestein, O. K. Farha, J. T. Hupp, MOF-enabled confinement and related effects for chemical catalyst presentation and utilization. *Chem. Soc. Rev.* **51**, 1045–1097 (2022).

27. Z. Sun, J. Hou, L. Li, Z. Tang, Nanoporous materials for chiral resolution. *Coord. Chem. Rev.* **425**, 213481 (2020).

28. J. Dong, Y. Liu, Y. Cui, Supramolecular chirality in metal-organic complexes. *Acc. Chem. Res.* **54**, 194–206 (2021).

29. J. Liu, S. Mukherjee, F. Wang, R. A. Fischer, J. Zhang, Homochiral metal-organic frameworks for enantioseparation. *Chem. Soc. Rev.* **50**, 5706–5745 (2021).

30. P. Peluso, B. Chankvetadze, Recognition in the domain of molecular chirality: From noncovalent interactions to separation of enantiomers. *Chem. Rev.* **122**, 13235–13400 (2022).

31. T. Iwasawa, R. J. Hooley, J. Rebek Jr., Stabilization of labile carbonyl addition intermediates by a synthetic receptor. *Science* **317**, 493–496 (2007).

32. A. B. Grommet, M. Feller, R. Klajn, Chemical reactivity under nanoconfinement. *Nat. Nanotechnol.* **15**, 256–271 (2020).

33. Y. Inokuma, S. Yoshioka, J. Ariyoshi, T. Arai, Y. Hitora, K. Takada, S. Matsunaga, K. Rissanen, M. Fujita, X-ray analysis on the nanogram to microgram scale using porous complexes. *Nature* **495**, 461–466 (2013).

34. D. Fiedler, R. G. Bergman, K. N. Raymond, Stabilization of reactive organometallic intermediates inside a self-assembled nanoscale host. *Angew. Chem. Int. Ed. Engl.* **45**, 745–748 (2006).

35. P. Mal, B. Breiner, K. Rissanen, J. R. Nitschke, White phosphorus is air-stable within a self-assembled tetrahedral capsule. *Science* **324**, 1697–1699 (2009).

36. A. Galan, P. Ballester, Stabilization of reactive species by supramolecular encapsulation. *Chem. Soc. Rev.* **45**, 1720–1737 (2016).

37. A. Widera, D. Thöny, M. Aebli, J. J. Oppenheim, J. L. Andrews, F. Eiler, M. Wörle, H. Schönberg, N. Weferling, M. Dincă, H. Grützmacher, Solid-state investigation, storage, and separation of pyrophoric PH_3 and P_2H_4 with α-Mg formate. *Angew. Chem. Int. Ed. Engl.* **62**, e202217534 (2023).

38. J. E. Mondloch, M. J. Katz, W. C. Isley, P. Ghosh, P. Liao, W. Bury, G. W. Wagner, M. G. Hall, J. B. Decoste, G. W. Peterson, R. Q. Snurr, C. J. Cramer, J. T. Hupp, O. K. Farha, Destruction of chemical warfare agents using metal-organic frameworks. *Nat. Mater.* **14**, 512–516 (2015).

39. N. S. Bobbitt, M. L. Mendonca, A. J. Howarth, T. Islamoglu, J. T. Hupp, O. K. Farha, R. Q. Snurr, Metal–organic frameworks for the removal of toxic industrial chemicals and chemical warfare agents. *Chem. Soc. Rev.* **46**, 3357–3385 (2017).

40. A. C. Sather, H. G. Lee, J. R. Colombe, A. Zhang, S. L. Buchwald, Dosage delivery of sensitive reagents enables glove-box-free synthesis. *Nature* **524**, 208–211 (2015).

41. P. Slavík, B. R. Trowse, P. O'Brien, D. K. Smith, Organogel delivery vehicles for the stabilization of organolithium reagents. *Nat. Chem.* **15**, 319–325 (2023).

42. A. Tortajada, E. Hevia, Stable organolithium gels. *Nat. Chem.* **15**, 299–300 (2023).

43. D. Seyferth, Zinc alkyls, Edward Frankland, and the beginnings of main-group organometallic chemistry. *Organometallics* **20**, 2940–2955 (2001).

44. A. Boudier, L. O. Bromm, M. Lotz, P. Knochel, New applications of polyfunctional organometallic compounds in organic synthesis. *Angew. Chem. Int. Ed. Engl.* **39**, 4414–4435 (2000).

45. L. Pu, H. B. Yu, Catalytic asymmetric organozinc additions to carbonyl compounds. *Chem. Rev.* **101**, 757–824 (2001).

46. T. Akindele, K. Yamada, K. Tomioka, Dimethylzinc-initiated radical reactions. *Acc. Chem. Res.* **42**, 345–355 (2009).

47. M. Kubisiak, K. Zelga, W. Bury, I. Justyniak, K. Budny-Godlewski, Z. Ochal, J. Lewiński, Development of zinc alkyl/air systems as radical initiators for organic reactions. *Chem. Sci.* **6**, 3102–3108 (2015).

48. S. V. Athavale, A. Simon, K. N. Houk, S. E. Denmark, Demystifying the asymmetry-amplifying, autocatalytic behaviour of the Soai reaction through structural, mechanistic and computational studies. *Nat. Chem.* **12**, 412–423 (2020).

49. M. Monge, M. L. Kahn, A. Maisonnat, B. Chaudret, Room-temperature organometallic synthesis of soluble and crystalline ZnO nanoparticles of controlled size and shape. *Angew. Chem. Int. Ed. Engl.* **42**, 5321–5324 (2003).

50. T. Weckman, K. Laasonen, Atomic layer deposition of zinc oxide: Study on the water pulse reactions from first-principles. *J. Phys. Chem. C Nanomater. Interfaces* **122**, 7685–7694 (2018).

51. R. D. Chavan, M. Wolska-Pietkiewicz, D. Prochowicz, M. Jędrzejewska, M. M. Tavakoli, P. Yadav, C. K. Hong, J. Lewiński, Organic ligand-free ZnO quantum dots for efficient and stable perovskite solar cells. *Adv. Funct. Mater.* **32**, 2205909 (2022).

52. M. Jędrzejewska, M. Wolska-Pietkiewicz, Z. Drużyński, J. Lewiński, Organometallic one-pot synthesis of ZnO quantum dots coated by sulfoxides as L-type ligands. *J. Mater. Chem. C* **11**, 15016–15029 (2023).

53. S. Bernhardt, G. Manolikakes, T. Kunz, P. Knochel, Preparation of solid salt-stabilized functionalized organozinc compounds and their application to cross-coupling and carbonyl addition reactions. *Angew. Chem. Int. Ed. Engl.* **50**, 9205–9209 (2011).

54. V. Dhayalan, V. S. Dodke, D. Sharma, R. Dandela, Recent advances in the preparation of air stable organozinc pivalates and their applications in organic synthesis. *Eur. J. Org. Chem.* **27**, e202301263 (2024).

55. K. Sokołowski, I. Justyniak, W. Śliwiński, K. Sołtys, A. Tulewicz, A. Kornowicz, R. Moszyński, J. Lipkowski, J. Lewiński, Towards a new family of photoluminescent organozinc 8-hydroxyquinolinates with a high propensity to form noncovalent porous materials. *Chem. A Eur. J.* **18**, 5637–5645 (2012).

56. A. Grala, M. Wolska-Pietkiewicz, A. Wojewódzka, M. Dabergut, I. Justyniak, J. Lewiński, Structural diversity of ethylzinc carboxylates. *Organometallics* **34**, 4959–4964 (2015).

57. M. Terlecki, I. Justyniak, D. Prochowicz, J. Lewiński, A new look on octet-compliant macrocyclic organoaluminum carboxylates as dormant poly-Lewis acids. *Eur. J. Inorg. Chem.* **2020**, 119–127 (2020).

58. J. Lewiński, T. Kaczorowski, D. Prochowicz, T. Lipińska, I. Justyniak, Z. Kaszkur, J. Lipkowski, Cinchona alkaloid-metal complexes: Noncovalent porous materials with unique gas separation properties. *Angew. Chem. Int. Ed. Engl.* **49**, 7035–7039 (2010).

59. T. Kaczorowski, I. Justyniak, D. Prochowicz, K. Zelga, A. Kornowicz, J. Lewiński, New insights into cinchonine-aluminium complexes and their application as chiral building blocks: Unprecedented ligand-exchange processes in the presence of ZnR_2 compounds. *Chem. A Eur. J.* **18**, 13460–13465 (2012).

60. C. Girard, H. B. Kagan, Nonlinear effects in asymmetric synthesis and stereoselective reactions: Ten years of investigation. *Angew. Chem. Int. Ed. Engl.* **37**, 2922–2959 (1998).

61. J. Lewiński, W. Marciniak, Z. Ochal, J. Lipkowski, I. Justyniak, A novel tetranuclear $[MeZn(\mu_3\text{-}OCH_2CH_2SMe)Zn(\mu\text{-}Cl)Me]_2$ adduct derived from the interaction of CH_2Cl_2 with an alkylzinc complex. *Eur. J. Inorg. Chem.* **2003**, 2753–2755 (2003).

62. M. Braun, The "Magic" diarylhydroxymethyl group. *Angew. Chem. Int. Ed. Engl.* **35**, 519–522 (1996).

63. M. Braun, The diaryl(oxy)methyl group: More than an innocent bystander in chiral auxiliaries, catalysts, and dopants. *Angew. Chem. Int. Ed. Engl.* **51**, 2550–2562 (2012).

64. J. Bacsa, F. Hanke, S. Hindley, R. Odedra, G. R. Darling, A. C. Jones, A. Steiner, The solid-state structures of dimethylzinc and diethylzinc. *Angew. Chem. Int. Ed. Engl.* **50**, 11685–11687 (2011).

65. J. Auld, D. J. Houlton, A. C. Jones, S. A. Rushworth, M. A. Malik, P. O'Brien, G. W. Critchlow, Growth of ZnO by MOCVD using alkylzinc alkoxides as single-source precursors. *J. Mater. Chem.* **4**, 1249 (1994).

66. E. Chwojnowska, M. Wolska-Pietkiewicz, J. Grzonka, J. Lewiński, An organometallic route to chiroptically active ZnO nanocrystals. *Nanoscale* **9**, 14782–14786 (2017).

67. D. Lee, M. Wolska-Pietkiewicz, S. Badoni, A. Grala, J. Lewiński, G. De Paëpe, Disclosing interfaces of ZnO nanocrystals using dynamic nuclear polarization: Sol-Gel versus organometallic approach. *Angew. Chem. Int. Ed. Engl.* **58**, 17163–17168 (2019).

68. M. Terlecki, S. Badoni, M. K. Leszczyński, S. Gierlotka, I. Justyniak, H. Okuno, M. Wolska-Pietkiewicz, D. Lee, G. De Paëpe, J. Lewiński, ZnO nanoplatelets with controlled thickness: Atomic insight into facet-specific bimodal ligand binding using DNP NMR. *Adv. Funct. Mater.* **31**, 2105318 (2021).

69. A. M. Cieślak, M. V. Pavliuk, L. D'Amario, M. Abdellah, K. Sokołowski, U. Rybinska, D. L. A. Fernandes, M. K. Leszczyński, F. Mamedov, A. M. El-Zhory, J. Föhlinger, A. Budinská, M. Wolska-Pietkiewicz, L. Hammarström, J. Lewiński, J. Sá, Ultra long-lived electron-hole separation within water-soluble colloidal ZnO nanocrystals: Prospective applications for solar energy production. *Nano Energy* **30**, 187–192 (2016).

70. J. Lewiński, W. Marciniak, J. Lipkowski, I. Justyniak, New insights into the reaction of zinc alkyls with dioxygen. *J. Am. Chem. Soc.* **125**, 12698–12699 (2003).

71.. Mąkolski, K. Zelga, R. Petrus, D. Kubicki, P. Zarzycki, P. Sobota, J. Lewiński, Probing the role of π interactions in the reactivity of oxygen species: A case of ethylzinc aryloxides with different dispositions of aromatic rings toward the metal center. *Chem. Eur. J.* **20**, 14790–14799 (2014).

72. KappaCCD Software; Nonius B.V.: Delft, The Netherlands (1998).

73. Z. Otwinowski, W. Minor, "[20] Processing of x-ray diffraction data collected in oscillation mode" in *Methods in Enzymology* (Academic Press, 1997) vol. 276, pp. 307–326. https://linkinghub.elsevier.com/retrieve/pii/S007668799776066X.

74. G. M. Sheldrick, Phase annealing in SHELX-90: Direct methods for larger structures. *Acta Cryst.* **46**, 467–473 (1990).

75. G. M. Sheldrick, *SHELX-97* (*Program for the Refinement of Crystal Structures*) (University of Göttingen, 1997).

76. R. Neufeld, D. Stalke, Accurate molecular weight determination of small molecules via DOSY-NMR by using external calibration curves with normalized diffusion coefficients. *Chem. Sci.* **6**, 3354–3364 (2015).

77. A. Kreyenschmidt, S. Bachmann, T. Niklas, D. Stalke, Molecular weight estimation of molecules incorporating heavier elements from van-der-Waals corrected ECC-DOSY. *ChemistrySelect* **2**, 6957–6960 (2017).

78. P. R. Spackman, M. J. Turner, J. J. McKinnon, S. K. Wolff, D. J. Grimwood, D. Jayatilaka, M. A. Spackman, *CrystalExplorer*: A program for Hirshfeld surface analysis, visualization and quantitative analysis of molecular crystals. *J. Appl. Cryst.* **54** (Pt. 3), 1006–1011 (2021).

79. M. A. Spackman, D. Jayatilaka, Hirshfeld surface analysis. *CrystEngComm* **11**, 19–32 (2009).

80. M. A. Spackman, J. J. McKinnon, D. Jayatilaka, Electrostatic potentials mapped on Hirshfeld surfaces provide direct insight into intermolecular interactions in crystals. *CrystEngComm* **10**, 377–388 (2008).

81. L. D. Gelb, K. E. Gubbins, Pore size distributions in porous glasses: A computer simulation study. *Langmuir* **15**, 305–308 (1999).

82. Materials Studio v 6.0. Accelrys Software Inc., San Diego, CA 92121, USA.

83. A. K. Rappe, C. J. Casewit, K. S. Colwell, W. A. Goddard, W. M. Skiff, UFF, a full periodic table force field for molecular mechanics and molecular dynamics simulations. *J. Am. Chem. Soc.* **114**, 10024–10035 (1992).
